# A Longitudinal Case-Based Global Health Curriculum for the Medical Student Clerkship Year

**DOI:** 10.15766/mep_2374-8265.11038

**Published:** 2020-12-08

**Authors:** Parisa N. Fallah, Gavin G. Ovsak, Jennifer Kasper, Robert Riviello, Yves Nezerwa, Bram Wispelwey, Nora Y. Osman, Navin L. Kumar

**Affiliations:** 1 Resident, Obstetrics and Gynecology, Brigham and Women's Hospital, Massachusetts General Hospital, and Harvard Medical School; 2 Resident, Anesthesiology, Brigham and Women's Hospital and Harvard Medical School; 3 Assistant Professor, Pediatrics and Global Health and Social Medicine, Brigham and Women's Hospital and Harvard Medical School; 4 Associate Professor, Surgery and Global Health and Social Medicine, Brigham and Women's Hospital and Harvard Medical School; 5 Plastic Surgery Resident, Rwanda Military Hospital and College of Surgeons of East, Central and Southern Africa (COSECSA); 6 Instructor, Internal Medicine and Global Health Equity, Brigham and Women's Hospital and Harvard Medical School; 7 Assistant Professor, Internal Medicine, Brigham and Women's Hospital and Harvard Medical School; 8 Instructor, Internal Medicine, Brigham and Women's Hospital and Harvard Medical School

**Keywords:** Global Health, Social Determinants of Health, Core Clerkships, Clinical Clerkship, Case-Based Learning, Clinical Education, Qualitative Study, Diversity, Inclusion, Health Equity, Editor's Choice

## Abstract

**Introduction:**

Over 20% of U.S. medical students express interest in global health (GH) and are searching for opportunities within the field. In addition, domestic practice increasingly requires an understanding of the social factors affecting patients’ health. Unfortunately, only 39% of medical schools offer formal GH education, and there is a need to incorporate more GH into medical school curricula.

**Methods:**

We designed a longitudinal case-based curriculum for the core clerkships. We conducted an institution-wide survey to determine baseline GH interest and developed three case-based sessions to incorporate into medicine, surgery, and pediatrics clerkships. The cases included clinical learning while exploring fundamental GH concepts. Cases were developed with GH faculty, and the pilot was implemented from October to December 2019 with 55 students. We used pre- and postdidactic surveys to assess interest in GH and elicit qualitative feedback. A follow-up survey assessed students’ identification of barriers faced by their patients domestically.

**Results:**

Students felt that clinical management, physical exam skills, epidemiology, and social determinants of health were strengths of the sessions and that they were able to apply more critical thinking skills and cultural humility to their patients afterwards. Students felt that simulation would be a great addition to the curriculum and wanted both more time per session and more sessions overall.

**Discussion:**

Integrating GH didactics into the core clerkships has potential to address gaps in GH education and to help students make connections between clinical learning and GH, enhancing their care of patients both domestically and in future GH work.

## Educational Objectives

By the end of this activity, learners will be able to:

1. Discuss social, economic, and cultural challenges faced by patients in low-resource settings and disparities in access to care globally.
2. Describe social determinants of health and their effects on patients domestically and internationally.
3. Apply new skills in history, physical exam, diagnosis, and treatment to patient care throughout the clerkships.

## Introduction

It is becoming increasingly important for medical schools to incorporate global health (GH) into their curricula. A significant percentage of students who enter medical school in the United States have an interest in GH, increasing from 6% in 1984 to 23% in 2007.^1^ Additionally, students are engaging in international experiences prior to medical school, and 20%–30% of medical students will take part in an international clinical elective during their training.^2–4^ However, it is not just students with an interest in GH who should be exposed to GH education. Domestic physician practice increasingly requires an understanding of the many social, cultural, and economic factors that affect patient outcomes,^5,6^ and there are a number of benefits to formally incorporating these concepts into medical education.^7^ GH education can help increase awareness of the role of public health in medicine, as well as the structural barriers to health care.^8,9^ Exposure to GH in medical school can also increase the chance of future practice in underserved areas of the U.S. and careers in primary care.^10–13^ Given the rapid rate of globalization, it is becoming increasingly important to foster deeper learning amongst medical students regarding the social determinants of health (SDH) and the historical determinants of health inequities, both major contributors to disparities in health care globally.

A consensus statement in *Academic Medicine* concluded that medical school GH curricula vary significantly and that the majority of GH experiences for medical students are international rotations.^3^ While medical schools offer preclerkship GH courses and postclerkship international electives, this leaves a significant gap during the core clinical year and is a missed opportunity to connect the SDH to students’ foundational clinical education. Existing GH curricular offerings are also often optional, which deepens learning for those who are interested but leaves many medical students without exposure to GH. To address the gap in GH coursework during the clerkship year, we developed a clinically focused case-based GH curriculum spread longitudinally throughout the medical student core clerkships.^14,15^ Cases were chosen from different World Health Organization regions internationally, while also addressing domestic concepts (e.g., refugee care, homeless populations, rural health care, indigenous health, etc.). The cases were intended to encourage students to think creatively, draw upon fundamental clinical knowledge and skills, connect historical processes with current health realities, and recognize the significant structural barriers for patients in underserved regions internationally and domestically.^16,17^ We also hoped to increase interest in GH and to expose students to GH faculty mentors throughout the clerkship year.

Our work builds on prior work published in the general medical education and GH literature, including in *MedEdPORTAL.* For example, a course entitled Clinical Topics in Global Health also took an integrated case-based approach^18^; however, our course is unique in that it is required for all students and spans the entire clerkship year, allowing students to directly engage in critical thinking and to apply their growing body of clinical knowledge to resource-constrained scenarios. Also in *MedEdPORTAL,* Fredrick discussed implementation of a single GH case addressing health disparities, health systems, and SDH.^19^ We built on this case model, expanding to several cases across the core clerkships, with a greater focus on clinical learning, and highlighting not only international but also domestic issues. With this curriculum, we hope to facilitate the development of a socially conscious generation of physicians who will look beyond their patients’ physical symptoms and work to understand health and well-being in the greater context of their patients’ lives.

## Methods

### Curricular Context

We implemented this curriculum in the didactic portion of three of the core clerkships for Harvard Medical School (HMS) students based at Brigham and Women's Hospital (BWH) and Boston Children's Hospital (BCH). The curriculum consisted of 1-hour sessions in medicine, surgery, and pediatrics, and attendance was required for clerkship students. At our institution, the medicine clerkship was 12 weeks long, with didactics included throughout. The surgery clerkship was 12 weeks long, with didactics scheduled in blocks every Wednesday morning. The pediatrics clerkship was 6 weeks long, with didactics scheduled throughout.

To advocate for the implementation of this curriculum, we sent out an interest survey to all Harvard medical students from May 12 to June 15, 2019, and then conducted a review of the GH course offerings at HMS. At the end of June 2019, this information was presented to the clerkship directors at BWH and BCH (Appendices A and B). We then met with the medicine, surgery, pediatrics, and OB/GYN clerkship directors throughout July 2019 to discuss moving forward with piloting the curriculum. We received approval from medicine, surgery, and pediatrics. Due to time constraints in the OB/GYN clerkship, we did not receive approval, and this was deferred for the purposes of the pilot study. We then contacted GH faculty in medicine, surgery, and pediatrics to begin developing the cases (Appendix B). This included a series of independent meetings throughout August and September 2019 to understand gaps in the current clerkship didactics, to discuss potential topics for the cases, and to begin outlining each session.

For pediatrics, there were no didactics on diarrheal disease and fluid management, so we elected to move forward with the topic of diarrhea, dehydration, and malnutrition, based in a Syrian refugee camp (Appendix C). For surgery, we saw that there were no didactics on burn care and decided on the topic of burns, plastic surgery, and vulnerable populations, based in rural Rwanda (Appendix D). For internal medicine, we engaged residents and faculty in the BWH GH equity residency, who chose a case on pneumonia, air pollution, and forced displacement, based in a Palestinian community in Israel (Appendix E). As the cases were being developed, we worked with the clerkship directors and coordinators to schedule the sessions. For the study pilot period from October through December 2019, we scheduled one session for medicine, one session for surgery, and two sessions for pediatrics.

### Implementation of the Curriculum

After the didactic sessions were developed, there was minimal preparation time for faculty facilitators prior to the session itself. Preparation first involved confirming the session's date, time, and location with the clerkship coordinator. For the location, we asked that the coordinators reserve rooms with chairs at a table or arranged in a circle, rather than in rows, to better facilitate a conversational atmosphere. The rooms each had a projector and screen to display the PowerPoint slides; however, if that is not possible for future implementation, the facilitator could provide a laptop or use a whiteboard to talk through the case instead. Typically, the faculty facilitator reviewed the case notes (under each slide in Appendices C–E and also listed in Appendix F) the evening prior to delivering the session. For the pediatrics session (Appendix C), the faculty facilitator prepared multiple pieces of string 11 cm long (one per student) for the mid-upper arm circumference slide. The surgery and internal medicine sessions did not require any special materials. Each session was scheduled for 1 hour, though it could easily be extended an additional 15–20 minutes, time permitting. The faculty facilitator notes under each slide in Appendices C–E, as well as in Appendix F, provide detailed explanation of prepared questions for students throughout the session and points to emphasize. They also indicate slides that can be quickly skipped should there be time constraints during the session.

### Survey Design and Administration

To assess the impact of this educational innovation, we administered pre- and postintervention surveys to explore student interest in GH, desire for increased GH education opportunities, likelihood to seek out GH experiences in medical school and residency, and likelihood to pursue a career in GH. We used 5-point Likert scales (1 = *least,* 5 = *most*) to assess these student perspective items. We also gathered demographic data and feedback on each session. The preintervention survey was administered during orientation (first day) for each clerkship (Appendix G). The postintervention survey was administered to students immediately after each didactic session (Appendix H). We also administered a brief follow-up survey with free-form questions 2 weeks after each session to assess how the cases impacted students’ views of their patients, whether they noticed any similarities or differences in their clinical experiences, and any additional reflections (Appendix I).

Per agreement with the clerkship directors, the surveys were administered only during the pilot period from October to December 2019. The total potential sample size during that time was 55 (14 students on internal medicine, 14 students on surgery, and a total of 27 students divided between two pediatrics blocks). The HMS/School of Public Health Institutional Review Board determined that the study was exempt from review.

### Data Analysis

We used counts and percentages to describe categorical variables, as well as means and standard deviations to describe continuous variables. Likert responses were analyzed as continuous variables. Qualitative data were analyzed via a grounded theory approach to identify themes on the educational experience, methods for improving implementation in the future, and impact of the curriculum on students’ views of patient care and the challenges their patients faced here in Boston. Student data were analyzed collectively in the preintervention and postintervention responses rather than being paired for direct comparison. It was determined that pairing would be challenging with limited time, would create issues with student confidentiality, and would result in lost data for students who may not have filled out one survey or the other. All analyses were conducted using Microsoft Excel.

## Results

Ninety-eight out of 98 of 660 students (15%) participated in the school-wide GH interest survey. Students from each class responded to the survey (28% MS 1s, 27% MS 2s, 31% MS 3s, and 15% MS 4s). Most students had an interest in internal medicine (36%), surgery (28%), or pediatrics (16%). A majority of respondents (69%) had prior experience in GH. Most students were extremely or very interested in GH (53%), were extremely or somewhat dissatisfied with the current GH curriculum at HMS (55%), and were extremely or very interested in having a GH curriculum during the clerkships (57%). Student comments mentioned not feeling prepared for GH work, the desire to gain clinical knowledge applicable to resource-limited settings, and the desire to interact with GH faculty.

For piloting the curriculum, preintervention surveys were administered to 55 students in the medicine, surgery, or pediatrics clerkships at BWH and BCH. Fifty-one of the 55 students (93%) filled out this survey. The majority of students in the pilot were in their MS 2 year (73%), were MD only (78%), and had an interest in internal medicine (63%), followed by pediatrics (49%), surgery (39%), and OB/GYN (29%). Additional demographic data from the predidactic survey are included in Table 1. In the predidactic survey, 45% of students were extremely or very interested in GH, 53% were extremely or somewhat likely to pursue GH experiences in medical school and 41% during residency, and 51% said they were extremely or somewhat likely to incorporate GH into their future career.

**Table 1.**
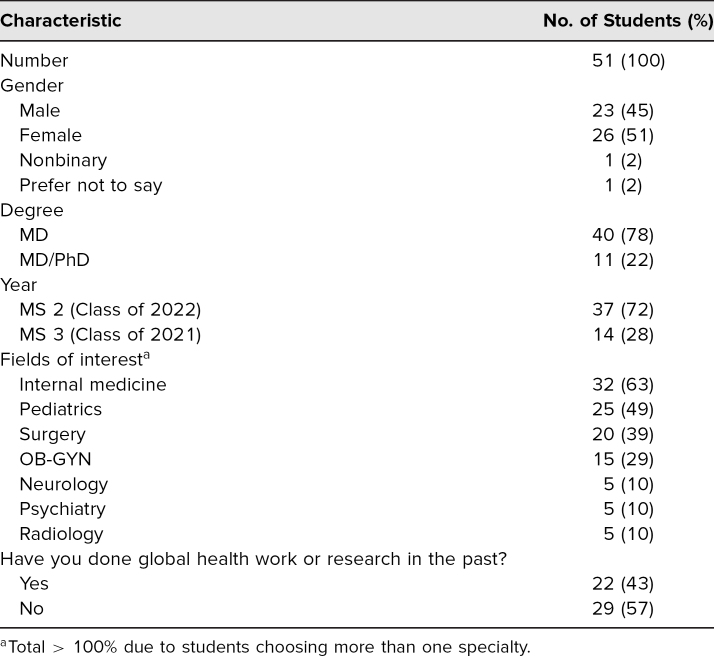
Predidactic Survey Demographics (*N* = 51)

The postdidactic survey was administered directly after each GH didactic session. Forty of the 55 students (73%) filled it out. In the postdidactic survey, 50% of respondents were extremely or very interested in GH, 53% were extremely or somewhat likely to pursue GH experiences in medical school and 50% during residency, and 60% were extremely or somewhat likely to incorporate GH into their future career. The majority of students were extremely or very interested in having more GH didactics during the clerkships (55%) and were extremely or very interested in having more GH education during medical school overall (55%). Averages and standard deviations from the pre- and postdidactic surveys are shown in Table 2.

**Table 2.**
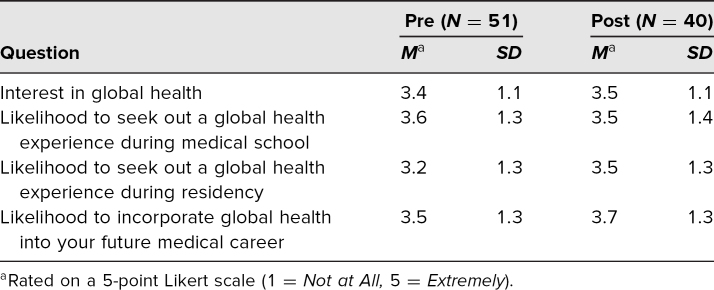
Pre- and Postdidactic Survey Quantitative Results

The postdidactic survey also included several qualitative questions to allow students to review the session and provide specific feedback. Students found clinical management, physical exam skills, epidemiology, creative approaches to care, and SDH to be key educational strengths of the sessions. Similarly, students wanted more clinical knowledge and epidemiology, more time to go through the cases in depth, and an even broader focus on GH concepts. Additional feedback indicated that students wanted more didactics such as these embedded throughout the clerkship year. These results are summarized in Table 3.

**Table 3.**
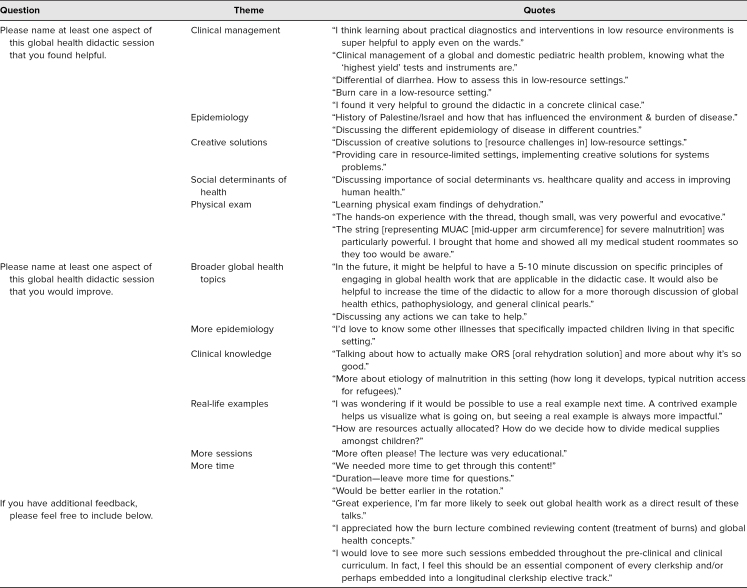
Postdidactic Survey Qualitative Results (*N* = 40)

The final follow-up survey was administered 2 weeks after each GH didactic session. Seventeen of the 55 students (31%) filled out the follow-up survey. When discussing influence of the sessions, students mentioned increased critical thinking skills, cultural humility, interest in GH, and understanding of SDH. They noticed similarities between health care access challenges abroad and with vulnerable populations here in the U.S. Many students provided feedback that simulation would be a great addition to the curriculum, that rural health should be further incorporated, and that they wanted more GH in the overall medical school curriculum. These results are summarized in Table 4.

**Table 4.**
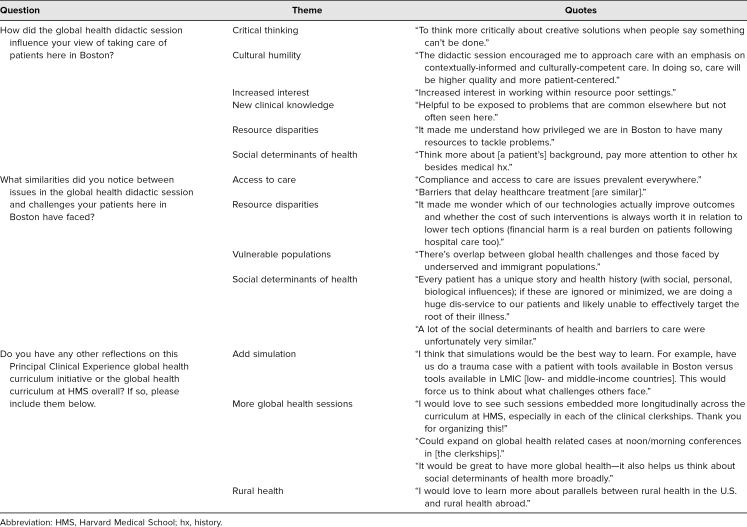
Follow-up Survey Qualitative Results (*N* = 17)

## Discussion

Increasing numbers of students enter medical school with an interest in GH, but not enough schools offer formal didactic GH education, and those that do often have a gap in GH education during the clerkship year. As globalization progresses, it is important for medical students to understand issues faced by patients both in their own locality and around the world. There are many demands on medical students’ time, and we see our GH curriculum as a way to incorporate GH into didactic sessions that address universally relevant clinical topics for the core clerkships. It is important for medical students to be exposed to this material during their clinical training, to demonstrate that GH concepts can enhance medical skills and professionalism for all students.

Each case in our curriculum uniquely intertwines clinical topics that students need for patient care (history, physical exam, diagnostics, treatment, etc.) with the challenges faced by patients in low- and middle-income countries, while also addressing SDH and barriers for patients in the U.S. As these sessions use a case-based approach, students are able to creatively engage with the issues presented in the case and draw upon their growing repertoire of clinical knowledge while learning about GH. This was clearly stated in the postdidactic survey responses by students who felt they gained clinical diagnosis and management skills as well as knowledge of epidemiology and SDH.

While there were encouraging results after implementation of this curriculum, there were challenges to implementation. One difficulty was the time constraints placed on any didactic offering within the core clerkships, as any time students spend in a didactic is less time spent clinically working with patients. Thus, there must be significant reason for adding to the clerkship didactic curriculum, and the curricular materials must be broadly applicable, relevant to patient care, and concise. It is also important to get buy-in from the clerkship leadership, which we initially found challenging and which led to us being unable to implement similar curricular materials in some of the other core clerkships. Please refer to Appendices A and B for materials to help support advocacy of inclusion of this curriculum.

We learned lessons from the didactic sessions themselves that we felt important to share with those who implement the sessions at their institutions. We found that while completing the session in 1 hour was possible, extending it by an additional 15 minutes allowed for more discussion and provided ample time for questions throughout. We also received direct verbal feedback from students indicating that the didactic sessions that engaged them the most in answering questions and critically thinking through the material were both more effective and more enjoyable. Additionally, students wanted the opportunity to hear from speakers about their GH experiences at the end of the session, which we encouraged our facilitators to do for subsequent iterations of the curriculum.

There are several limitations to both the curriculum and our approach to evaluation. Given the limited time allotted for piloting the curriculum, the sample size was small, representing only a subset of all students in their clerkship year at only two of the Harvard-affiliated hospitals. In addition, because of the 3-month time frame, students were evaluated after only one didactic session, while the curriculum is intended to be longitudinal. Ideally, in future iterations, the curriculum could be reevaluated over the course of a year, after students have been exposed to all of the GH didactic sessions across the different clerkships. Another challenge we faced was the survey response rate. While almost all students filled out the predidactic survey, the response rate decreased for the postdidactic survey and dropped off significantly for the follow-up survey. This made it challenging to conduct a true quantitative pre/post comparison of interest in pursuing GH and incorporating GH experiences into future training. However, we were able to gain significant insight from the qualitative data that showed meaningful gains for students who experienced the case-based GH didactic. We were unable to include the OB/GYN didactic session due to time constraints in the OB/GYN clerkship schedule, and we were unable to actively pursue the other core clerkships (neurology, psychiatry, and radiology) for a similar reason. Given the positive student feedback we received for the pilot, we are equipped to reevaluate the current clinical didactic offerings in those clerkships and to incorporate similar GH cases and content.

An additional limitation is that the survey tool does not align directly with the curriculum objectives. The objectives were developed to reflect the goals of curriculum implementation, whereas the survey tool was intended to collect feedback on the curriculum and assess student interest in GH. Given that students already face multiple evaluations throughout their clerkship year, including supervised history and physical exams, mock oral board exams, and shelf exams, it was difficult to implement an additional evaluative component of this curriculum based on the objectives. However, at institutions with additional space to incorporate an evaluative component of this curriculum, we suggest a supervised history and physical exam of a patient for whom SDH play an important factor in presentation, care, and outcomes. This provides an opportunity not only to evaluate students’ clinical skills but also to evaluate their growth in understanding and applying concepts outlined by the curriculum.

There are a number of exciting opportunities ahead based on this work. Expanding the case series to include the other core clerkships is an important first step. We may also consider developing subspecialty cases, for use in third- and fourth-year medical student electives or with residents. To address generalizability and to support institutions with fewer GH faculty, we have included detailed notes for each case and slide deck so that they can be directly taught by faculty or residents at other institutions. However, we also hope that the general structure we have created allows other institutions to adapt and expand the cases to cover topics that fit better into their current curricular offerings and address more locally relevant content.

Overall, we believe that this unique case-based approach makes GH education more accessible, interesting, and relevant to clerkship students, whether or not they plan to pursue GH during their careers. This currululum meaningfully includes GH education in medical school in a way that integrates both clinical learning and a greater understanding of the multidimensional factors affecting health outcomes. Ultimately, we envision that such cases will facilitate the development of a thoughtful and socially conscious generation of physicians who go beyond their immediate vicinity and engage with the broader context of the patients both in front of them and around the world.

## Appendices

Clerkship Director Proposal.pptxProject Description.docxPediatrics GH Didactic.pptxSurgery GH Didactic.pptxMedicine GH Didactic.pptxFacilitator Notes.docxPredidactic Survey.docxPostdidactic Survey.docxFollow-up Survey.docx
All appendices are peer reviewed as integral parts of the Original Publication.
