# Supplementary material for: A Longitudinal Case-Based Global Health Curriculum for the Medical Student Clerkship Year
Source: MedEdPORTAL. 2020 Dec 8;16:11038. doi: 10.15766/mep_2374-8265.11038 (PMC7732136; doi:10.15766/mep_2374-8265.11038)
Supplement: Supplementary file 1 — Clerkship Director Proposal.pptxProject Description.docxPediatrics GH Didactic.pptxSurgery GH Didactic.pptxMedicine GH Didactic.pptxFacilitator Notes.docxPredidactic Survey.docxPostdidactic Survey.docxFollow-up Survey.docx [file mep_2374-8265.11038-s001.zip › A. Clerkship Director Proposal.pptx]

## Slide 1
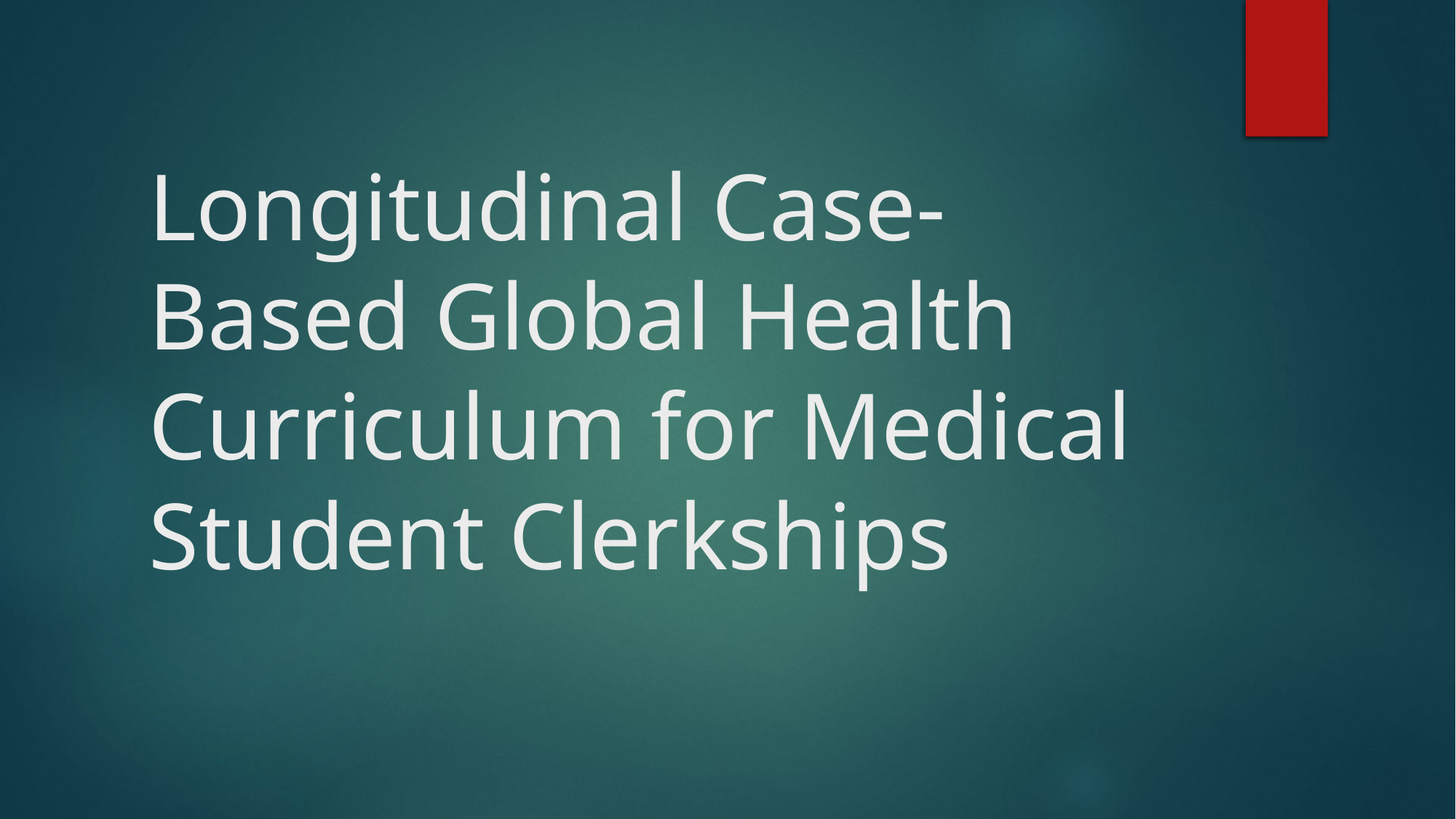

# Longitudinal Case-Based Global Health Curriculum for Medical Student Clerkships

## Slide 2
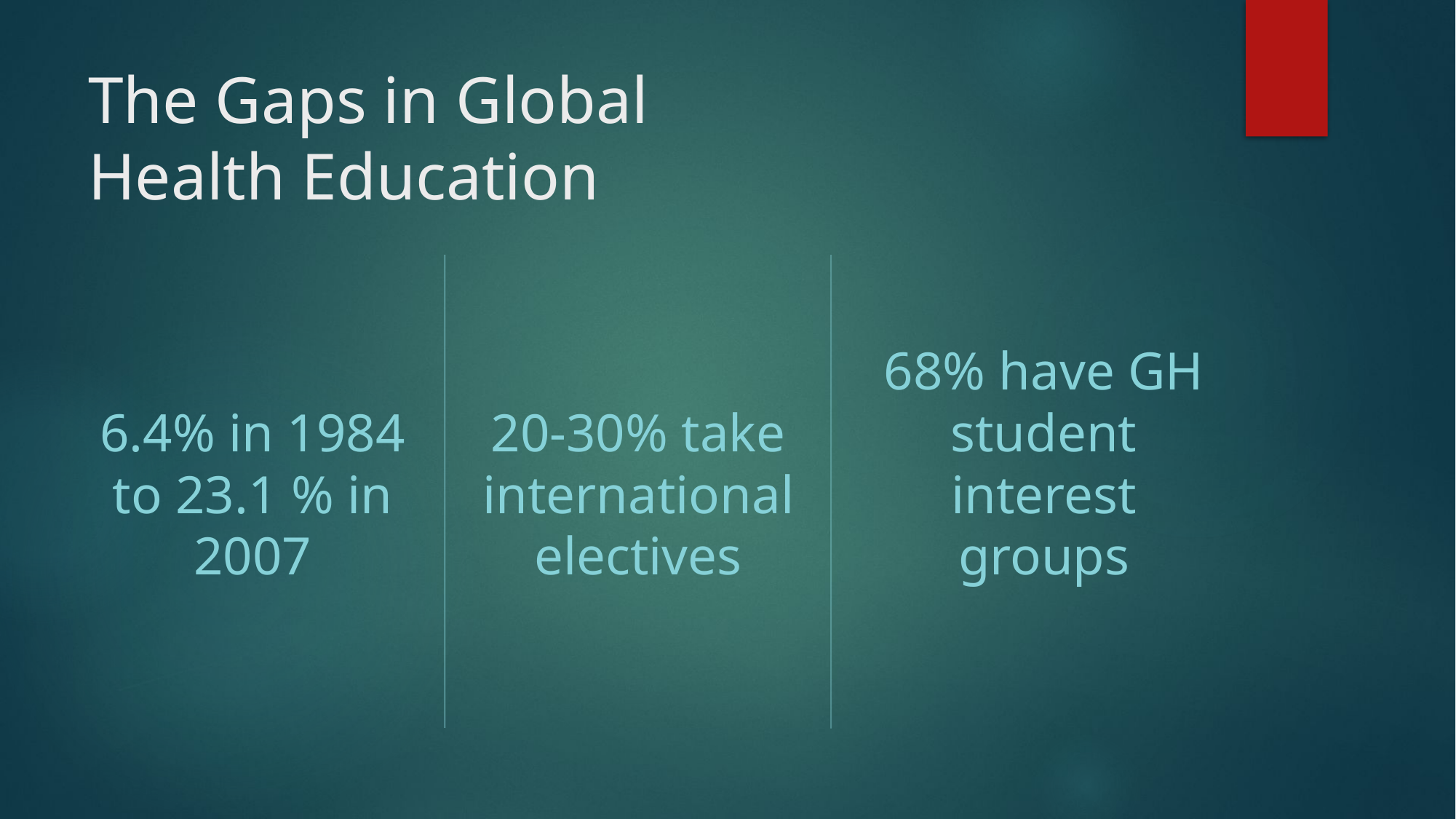

# The Gaps in Global Health Education
6.4% in 1984 to 23.1 % in 2007
20-30% take international electives
68% have GH student interest groups

## Slide 3
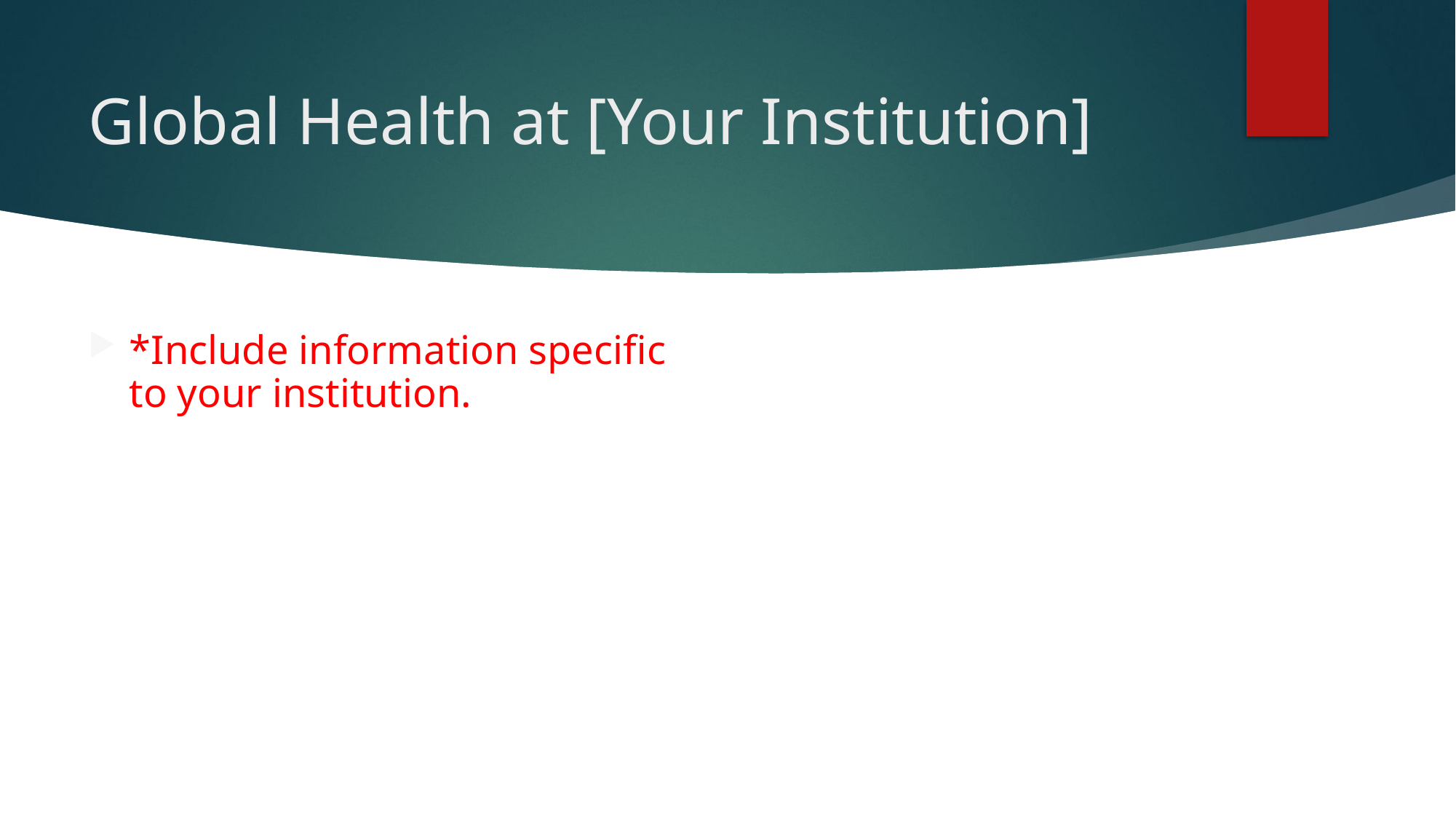

# Global Health at [Your Institution]
*Include information specific to your institution.

## Slide 4
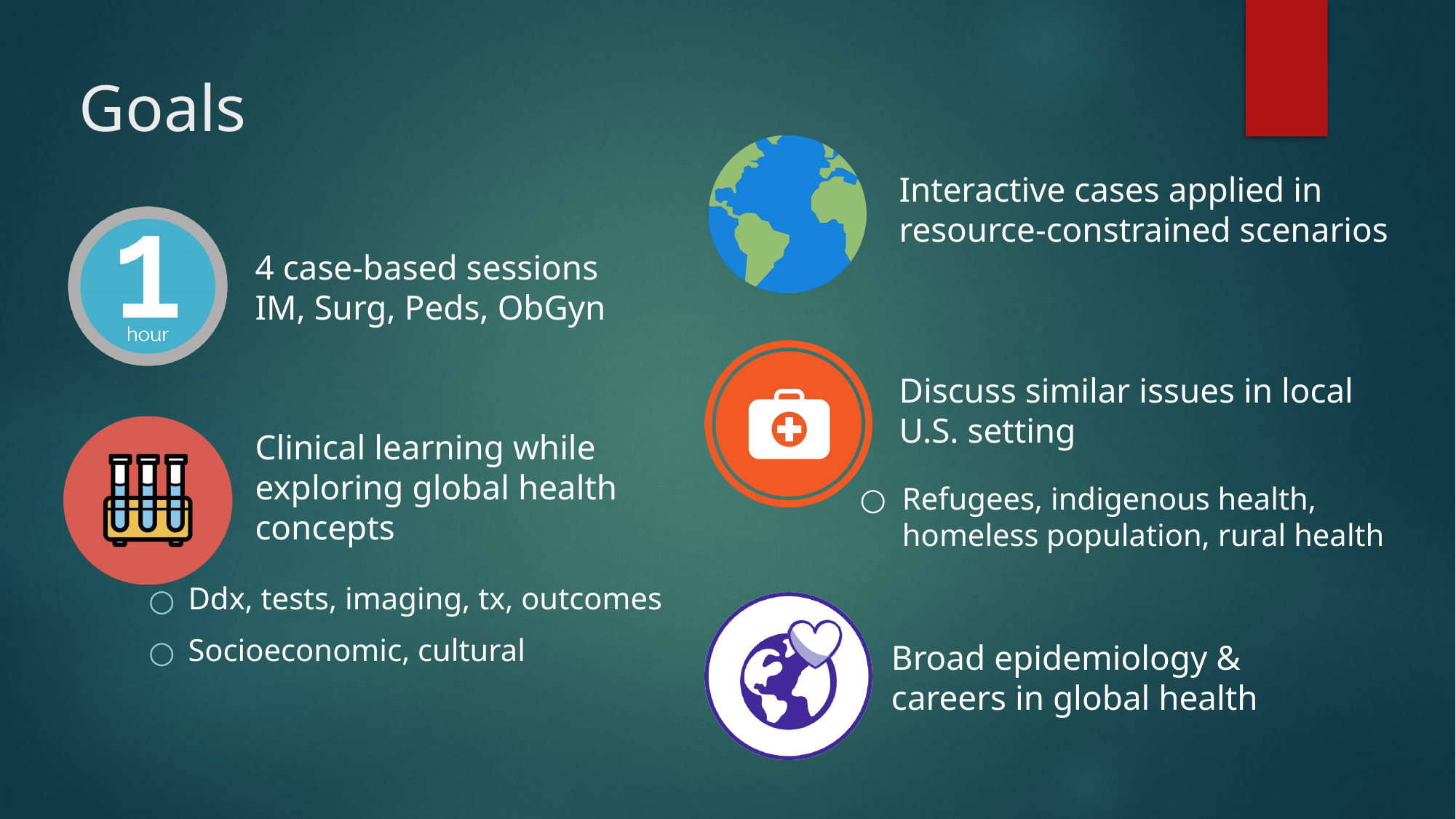

# Goals
Interactive cases applied in resource-constrained scenarios
4 case-based sessions
IM, Surg, Peds, ObGyn
Discuss similar issues in local U.S. setting
Refugees, indigenous health, homeless population, rural health
Clinical learning while exploring global health concepts
Ddx, tests, imaging, tx, outcomes
Socioeconomic, cultural
Broad epidemiology & careers in global health

## Slide 5
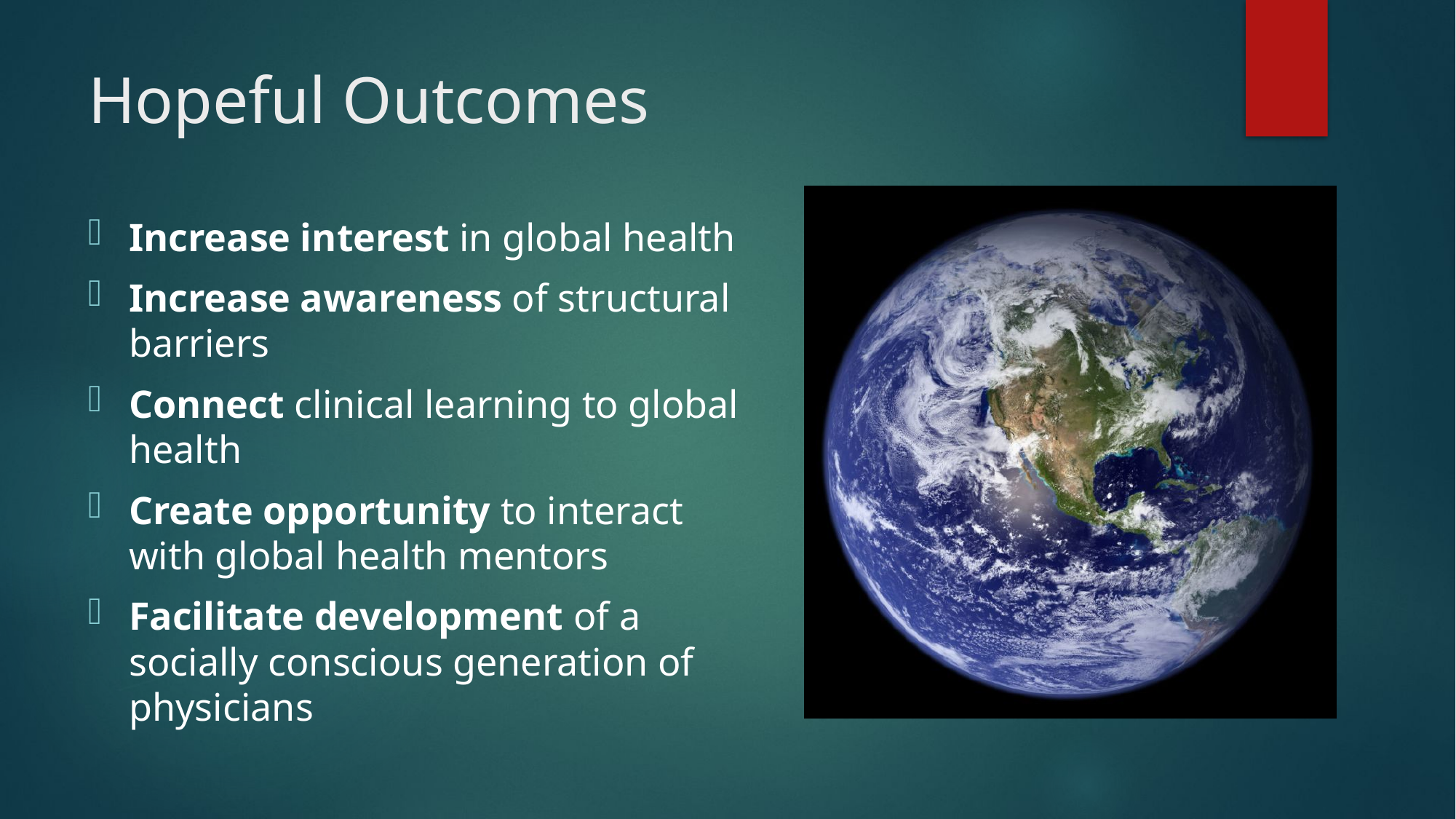

# Hopeful Outcomes
Increase interest in global health
Increase awareness of structural barriers
Connect clinical learning to global health
Create opportunity to interact with global health mentors
Facilitate development of a socially conscious generation of physicians

## Slide 6
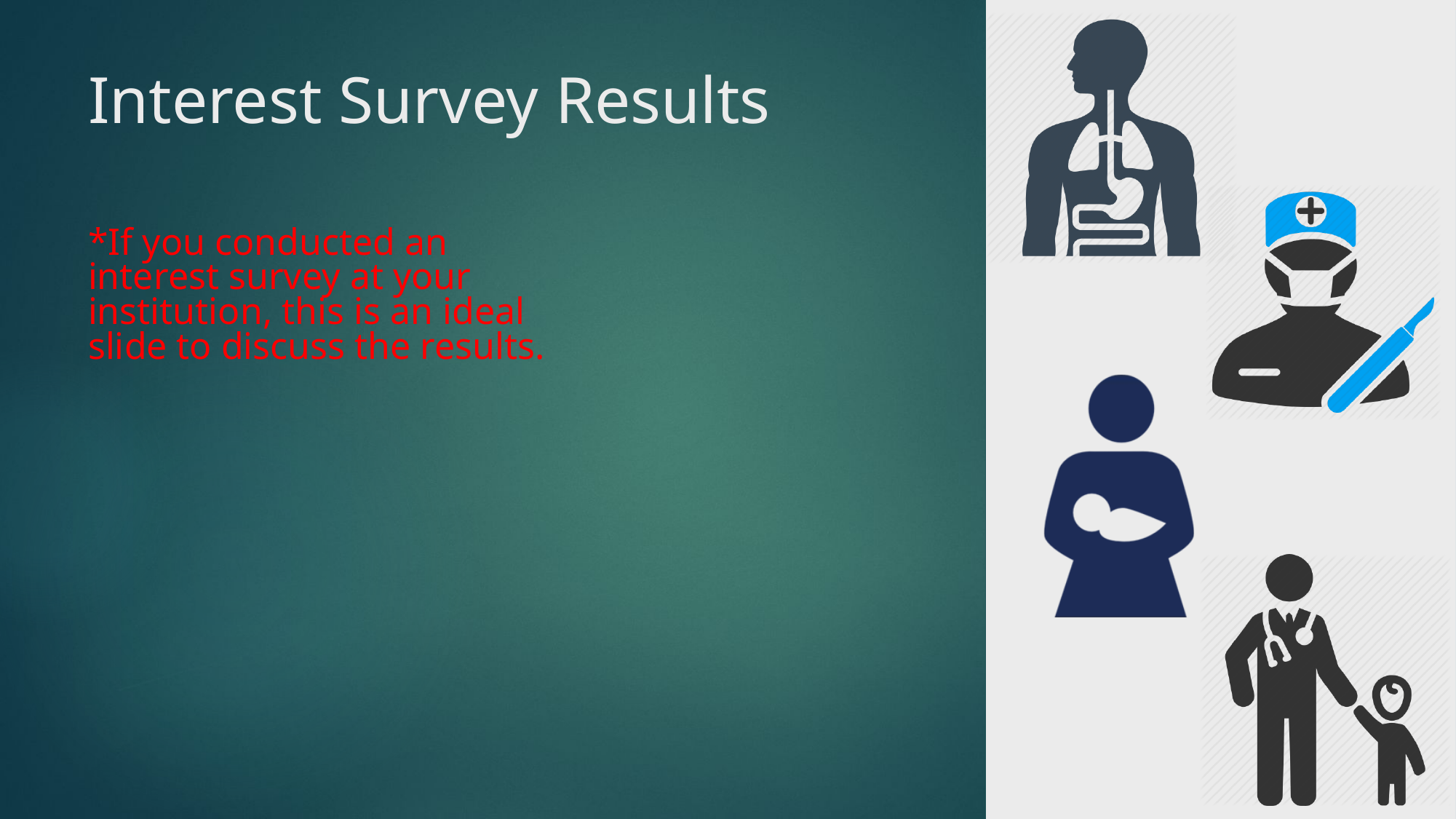

# Interest Survey Results
*If you conducted an interest survey at your institution, this is an ideal slide to discuss the results.

## Slide 7
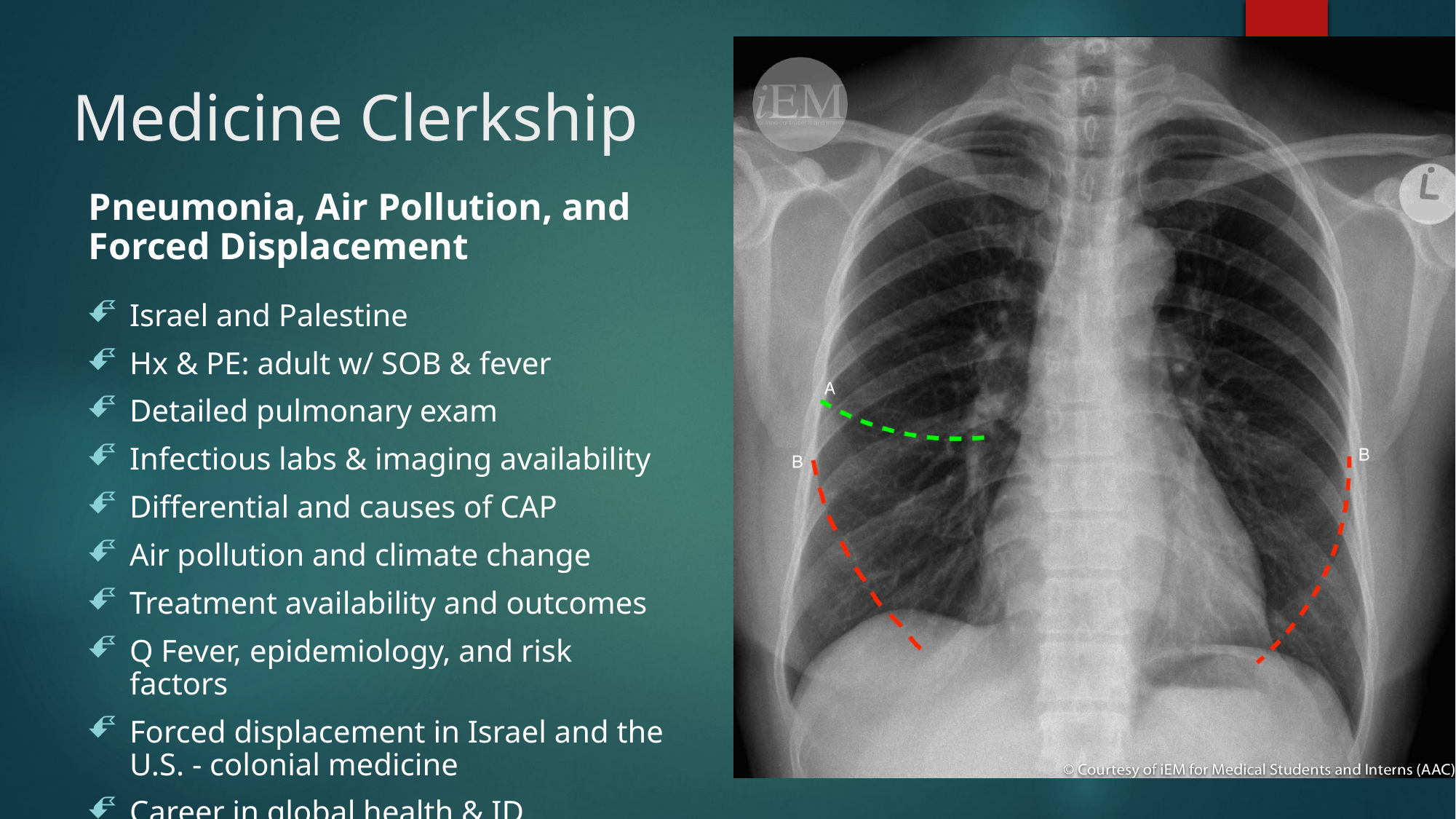

# Medicine Clerkship
Pneumonia, Air Pollution, and Forced Displacement
Israel and Palestine
Hx & PE: adult w/ SOB & fever
Detailed pulmonary exam
Infectious labs & imaging availability
Differential and causes of CAP
Air pollution and climate change
Treatment availability and outcomes
Q Fever, epidemiology, and risk factors
Forced displacement in Israel and the U.S. - colonial medicine
Career in global health & ID

## Slide 8
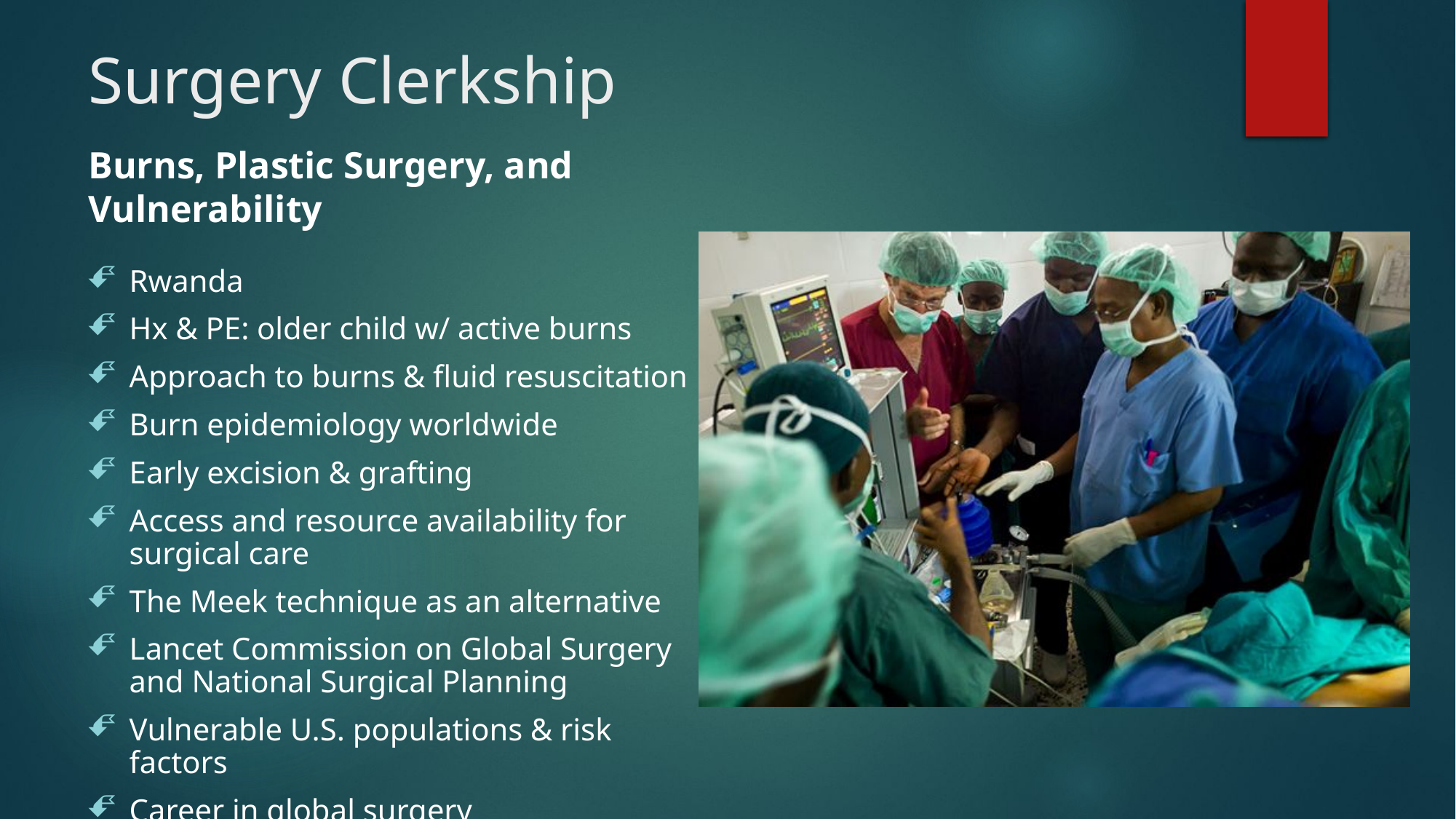

# Surgery Clerkship
Burns, Plastic Surgery, and Vulnerability
Rwanda
Hx & PE: older child w/ active burns
Approach to burns & fluid resuscitation
Burn epidemiology worldwide
Early excision & grafting
Access and resource availability for surgical care
The Meek technique as an alternative
Lancet Commission on Global Surgery and National Surgical Planning
Vulnerable U.S. populations & risk factors
Career in global surgery

## Slide 9
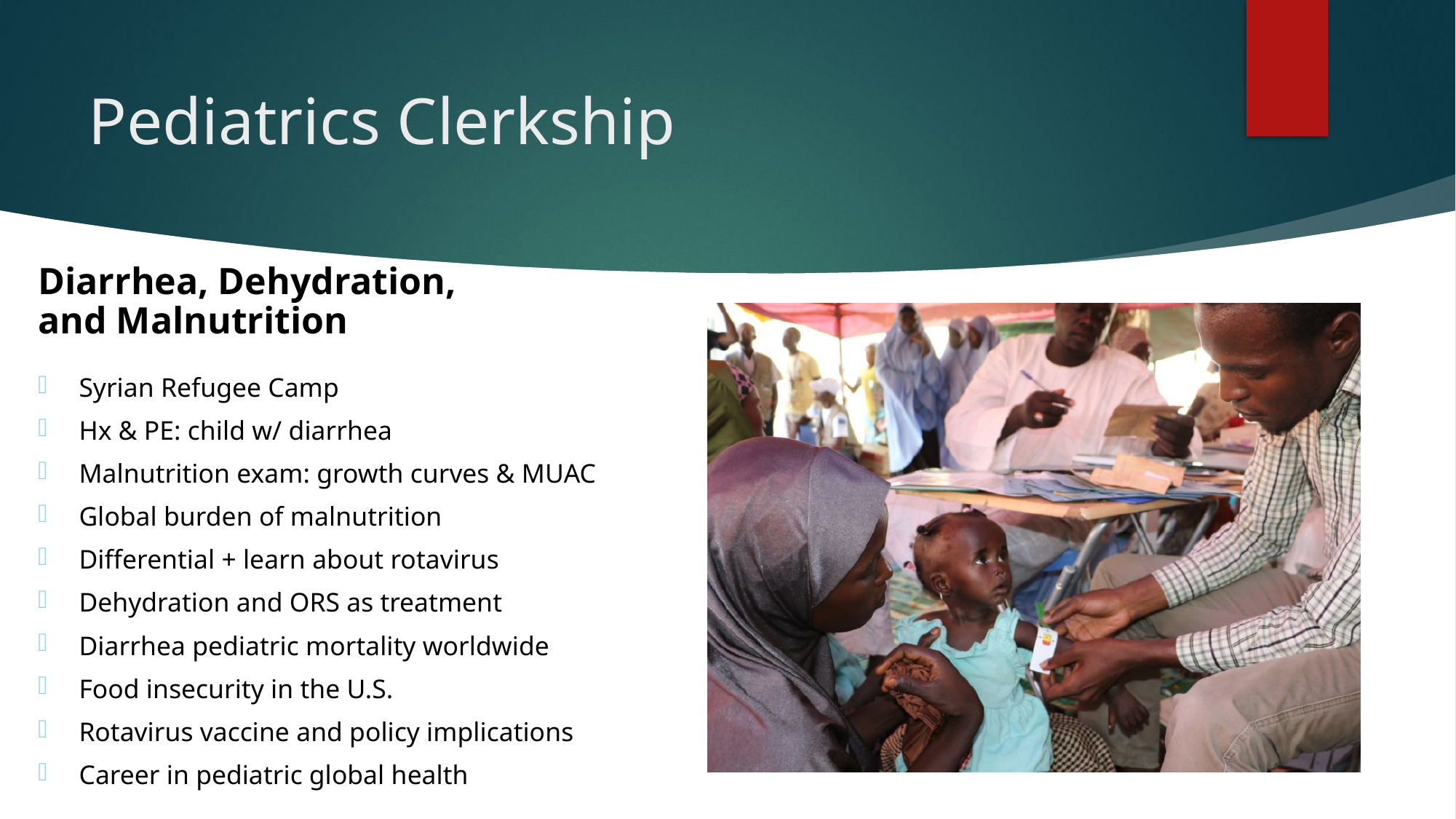

# Pediatrics Clerkship
Diarrhea, Dehydration,
and Malnutrition
Syrian Refugee Camp
Hx & PE: child w/ diarrhea
Malnutrition exam: growth curves & MUAC
Global burden of malnutrition
Differential + learn about rotavirus
Dehydration and ORS as treatment
Diarrhea pediatric mortality worldwide
Food insecurity in the U.S.
Rotavirus vaccine and policy implications
Career in pediatric global health

## Slide 10
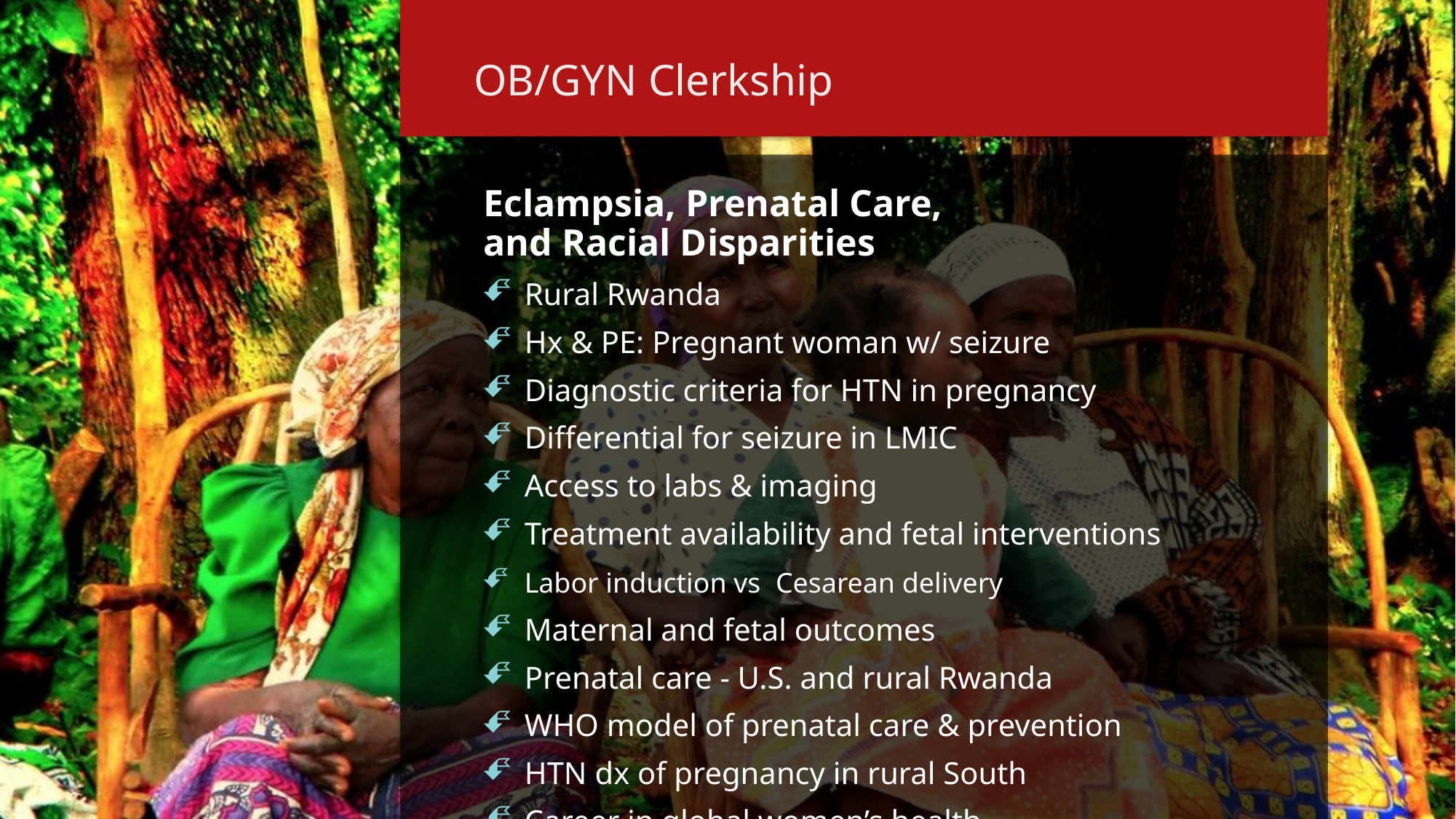

# OB/GYN Clerkship
Eclampsia, Prenatal Care,
and Racial Disparities
Rural Rwanda
Hx & PE: Pregnant woman w/ seizure
Diagnostic criteria for HTN in pregnancy
Differential for seizure in LMIC
Access to labs & imaging
Treatment availability and fetal interventions
Labor induction vs Cesarean delivery
Maternal and fetal outcomes
Prenatal care - U.S. and rural Rwanda
WHO model of prenatal care & prevention
HTN dx of pregnancy in rural South
Career in global women’s health

## Slide 11
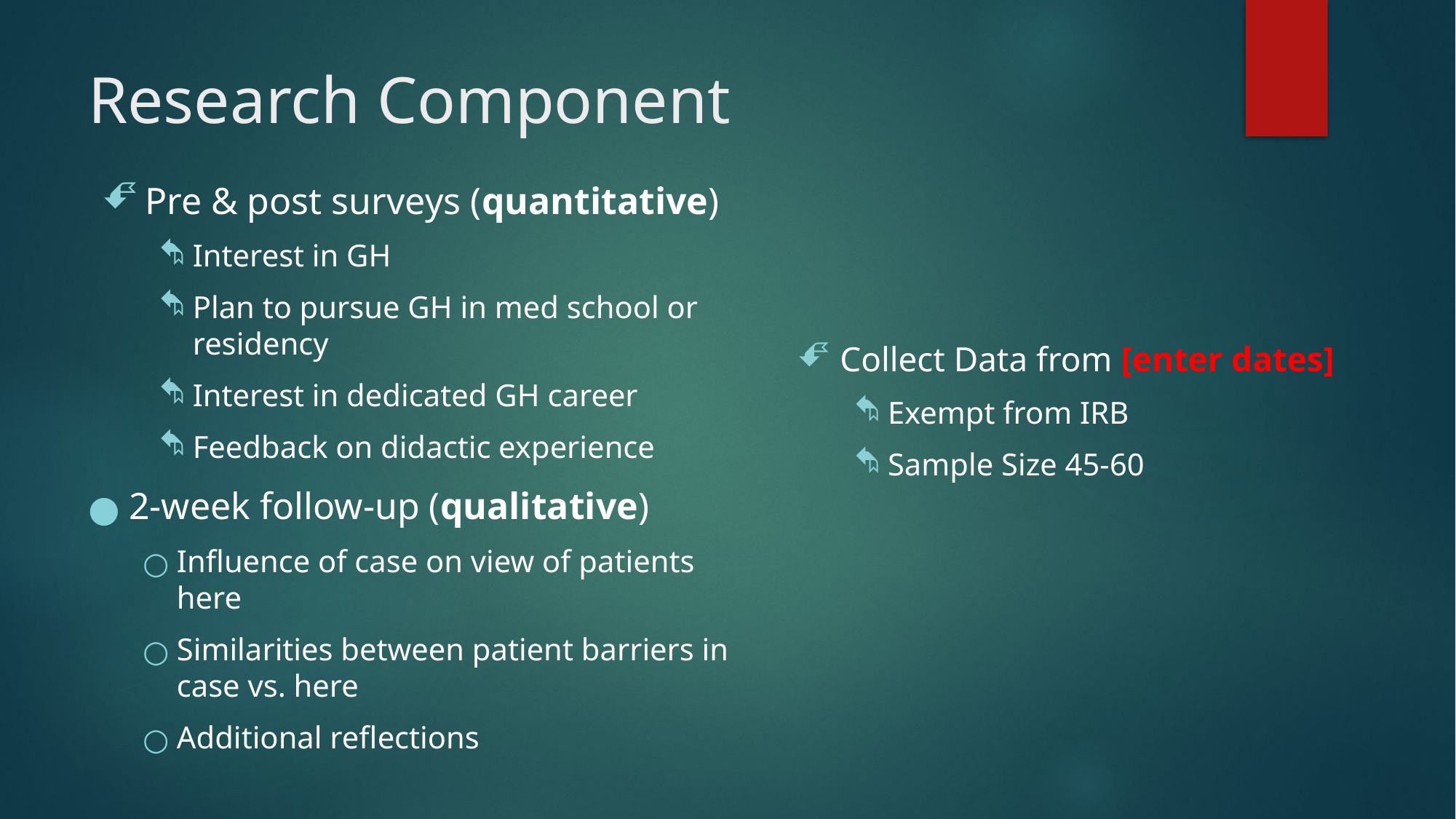

# Research Component
Pre & post surveys (quantitative)
Interest in GH
Plan to pursue GH in med school or residency
Interest in dedicated GH career
Feedback on didactic experience
Collect Data from [enter dates]
Exempt from IRB
Sample Size 45-60
2-week follow-up (qualitative)
Influence of case on view of patients here
Similarities between patient barriers in case vs. here
Additional reflections

## Slide 12
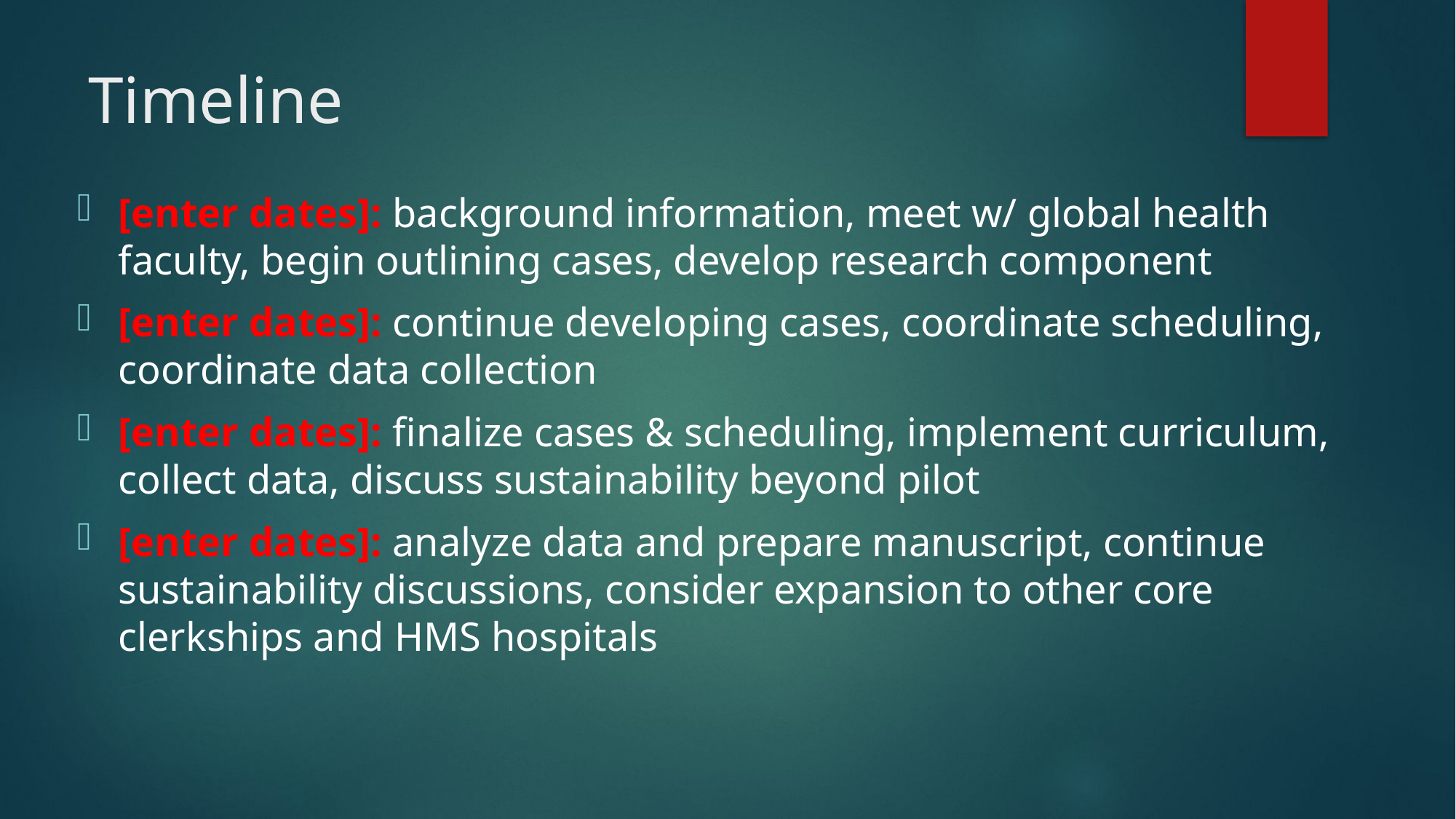

# Timeline
[enter dates]: background information, meet w/ global health faculty, begin outlining cases, develop research component
[enter dates]: continue developing cases, coordinate scheduling, coordinate data collection
[enter dates]: finalize cases & scheduling, implement curriculum, collect data, discuss sustainability beyond pilot
[enter dates]: analyze data and prepare manuscript, continue sustainability discussions, consider expansion to other core clerkships and HMS hospitals

## Slide 13
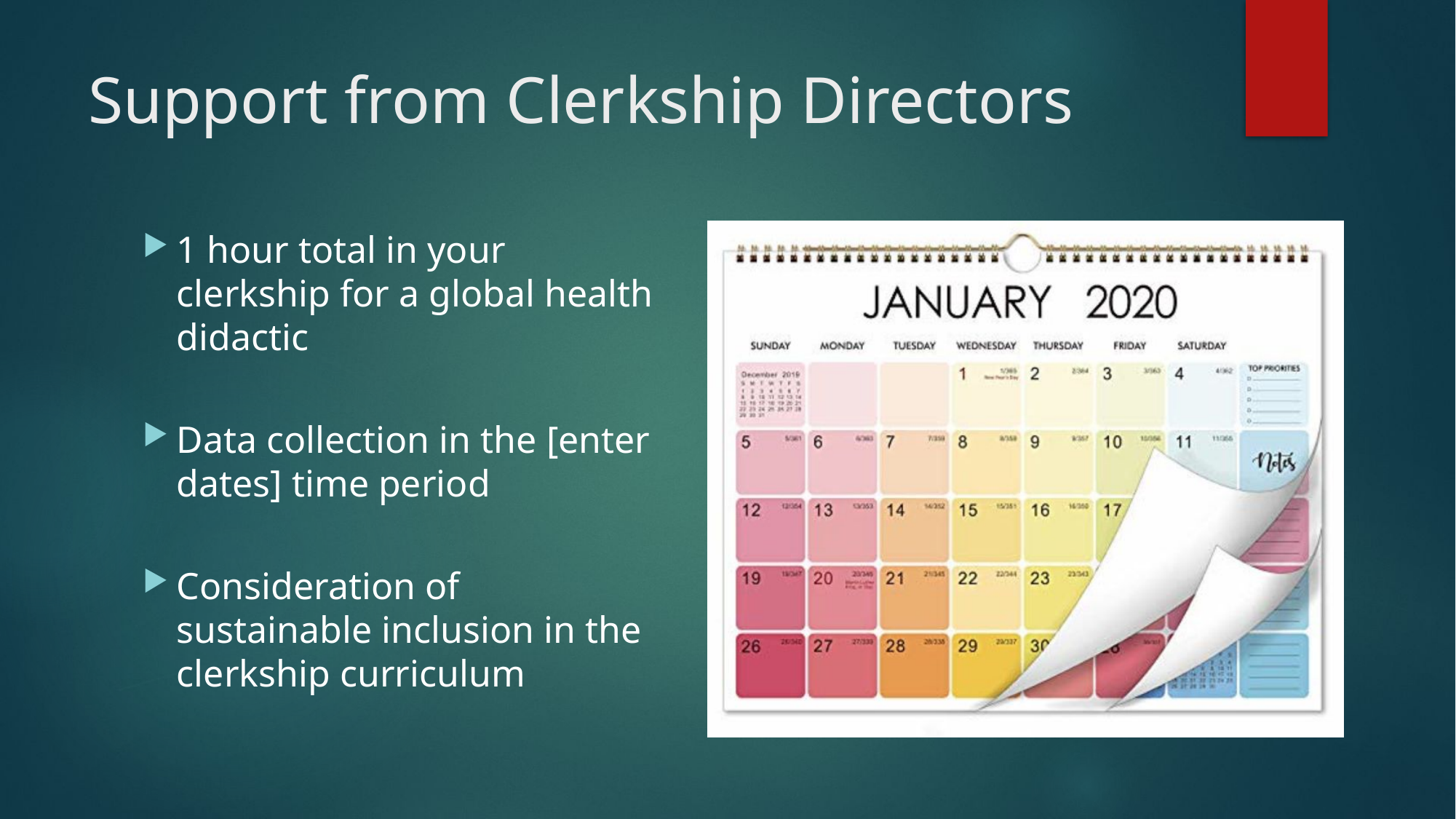

# Support from Clerkship Directors
1 hour total in your clerkship for a global health didactic
Data collection in the [enter dates] time period
Consideration of sustainable inclusion in the clerkship curriculum

## Slide 14
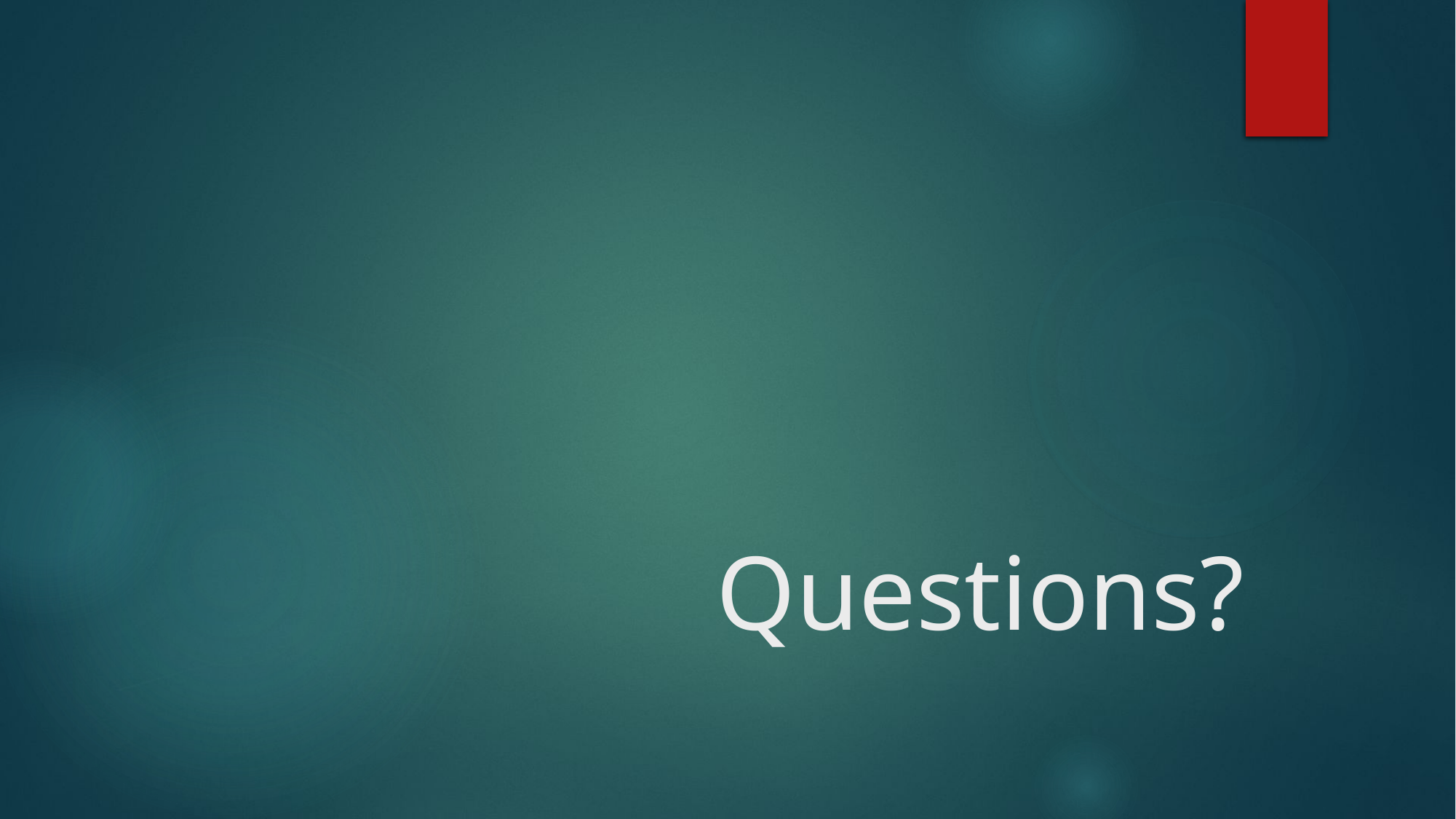

# Questions?
