# Supplementary material for: A Longitudinal Case-Based Global Health Curriculum for the Medical Student Clerkship Year
Source: MedEdPORTAL. 2020 Dec 8;16:11038. doi: 10.15766/mep_2374-8265.11038 (PMC7732136; doi:10.15766/mep_2374-8265.11038)
Supplement: Supplementary file 1 — Clerkship Director Proposal.pptxProject Description.docxPediatrics GH Didactic.pptxSurgery GH Didactic.pptxMedicine GH Didactic.pptxFacilitator Notes.docxPredidactic Survey.docxPostdidactic Survey.docxFollow-up Survey.docx [file mep_2374-8265.11038-s001.zip › B. Project Description.docx]

**Longitudinal Case-Based Global Health Curriculum for the Clerkships**

We plan to implement a clinically focused case-based global health curriculum that is spread longitudinally throughout the core clerkships. This curriculum would be required for all students, such that those interested in global health could learn more and those who are not could gain increased understanding of what is happening to underserved populations both domestically and internationally. The curriculum will be interactive, such that students could apply knowledge from their clinical learning to situations where they may not have access to as many specialists, medical equipment, facilities, and more. The hope is that this would encourage students to think outside the box, draw on fundamental clinical knowledge and skills, and also recognize the significant barriers faced by patients in underserved areas worldwide when trying to access healthcare.

There will be multiple layers within this curriculum, which will be specifically geared towards the Internal Medicine, Surgery, Pediatrics, and Ob/Gyn clerkships for the purposes of this pilot phase. We aim to include cases from different WHO regions internationally, while tying in concepts to local issues (i.e. refugee care, homeless population, indigenous health, etc.). This would emphasize clinical education and thoughtfulness around differential diagnoses, tests, imaging, criteria for a formal diagnosis, and treatment options, as well as how these aspects of care differ between the two settings. We will tie in social, cultural, and structural issues as the patients in each case face barriers to accessing healthcare. A final component is broadly discussing global health within that specialty and a career in the field so that students can also gain an understanding of the general landscape and epidemiology. The goal is for each case to take an hour, such that it can be incorporated into clerkship didactics, while also being rich in content and interactive for students.

The overall aims of the project include the following:

- Increase interest in global health amongst students, and potentially orient more students towards careers in the field
- Increase awareness of social, cultural, financial, and structural barriers in access to healthcare amongst their patients
- Connect clinical learning during clerkships to global health issues
- Create an opportunity for students to interact with residents/faculty involved in global health and get guidance on careers in the field
- Facilitate the development of a thoughtful and socially conscious generation of physicians that are aware of global health issues, both domestically and internationally
