# Supplementary material for: A Longitudinal Case-Based Global Health Curriculum for the Medical Student Clerkship Year
Source: MedEdPORTAL. 2020 Dec 8;16:11038. doi: 10.15766/mep_2374-8265.11038 (PMC7732136; doi:10.15766/mep_2374-8265.11038)
Supplement: Supplementary file 1 — Clerkship Director Proposal.pptxProject Description.docxPediatrics GH Didactic.pptxSurgery GH Didactic.pptxMedicine GH Didactic.pptxFacilitator Notes.docxPredidactic Survey.docxPostdidactic Survey.docxFollow-up Survey.docx [file mep_2374-8265.11038-s001.zip › C. Pediatrics GH Didactic.pptx]

## Slide 1
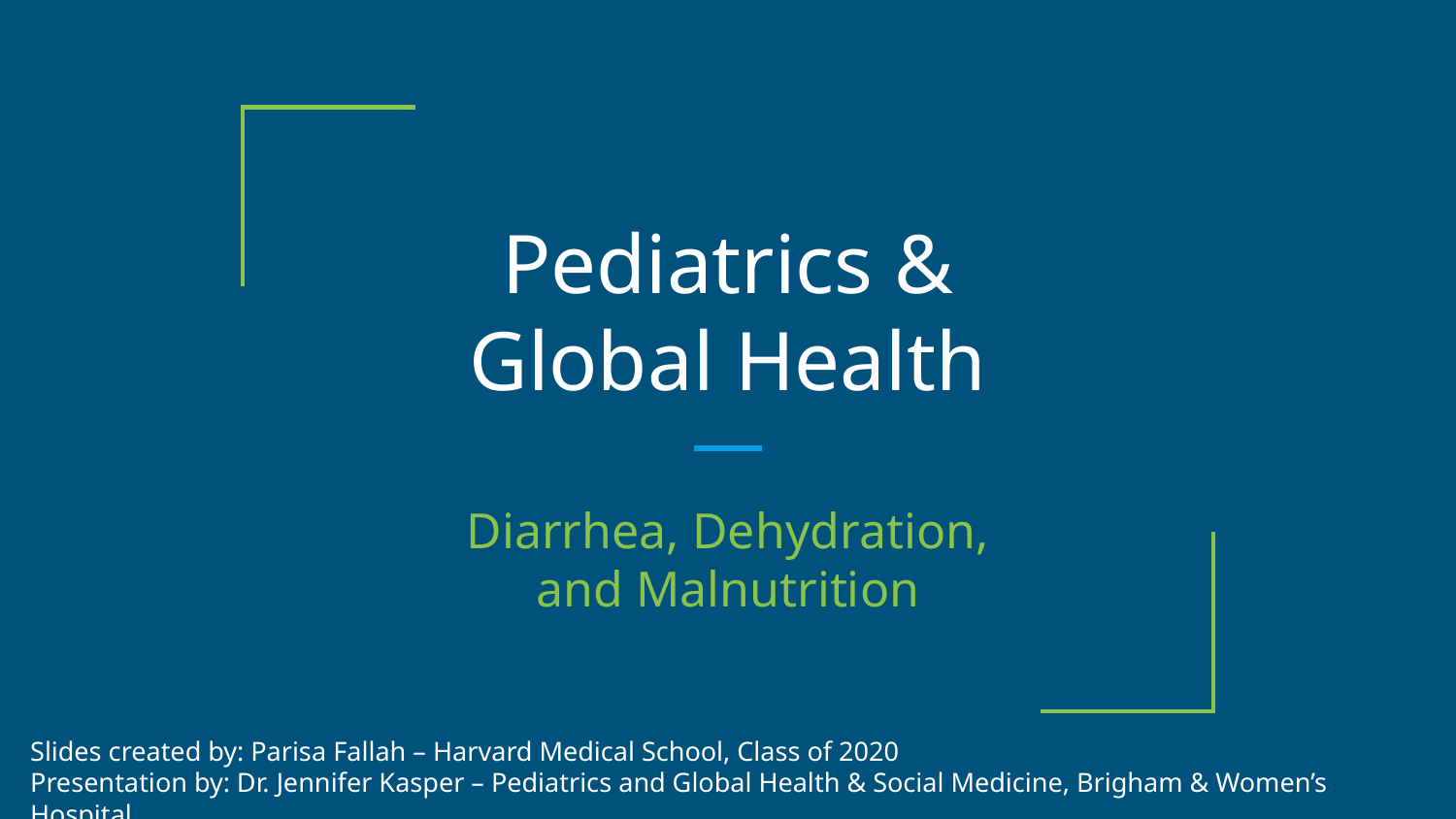

# Pediatrics &
Global Health
Diarrhea, Dehydration,
and Malnutrition
Slides created by: Parisa Fallah – Harvard Medical School, Class of 2020
Presentation by: Dr. Jennifer Kasper – Pediatrics and Global Health & Social Medicine, Brigham & Women’s Hospital

## Slide 2
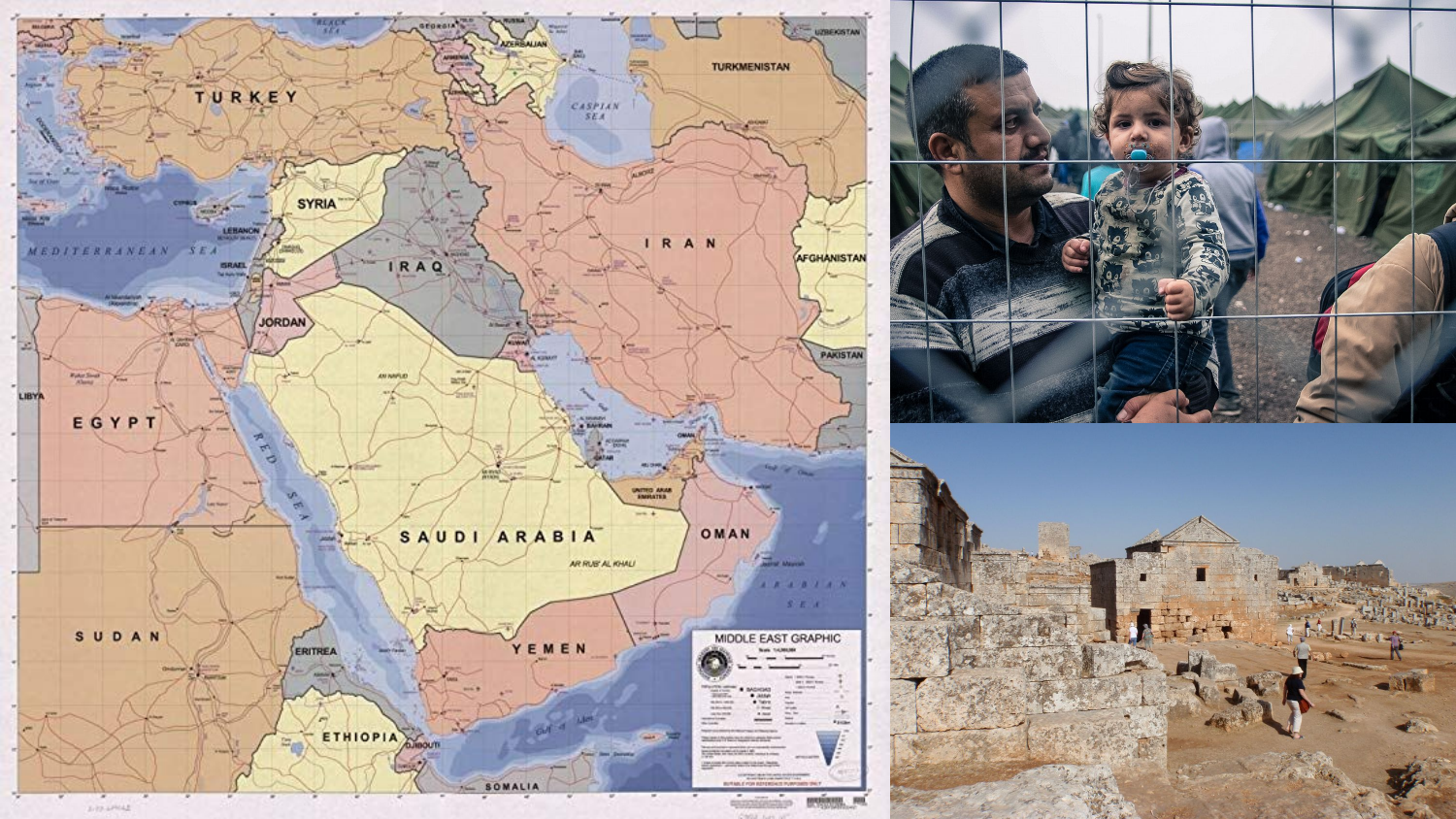

# Syrian Refugee Camp
Working at a clinic on-site at the camp
People living in tents w/ cots
ORS
Portable water
Chlorine, iodine
Limited IV fluids
Unable to draw labs
Thermometers
One adult-sized blood pressure cuff

## Slide 3
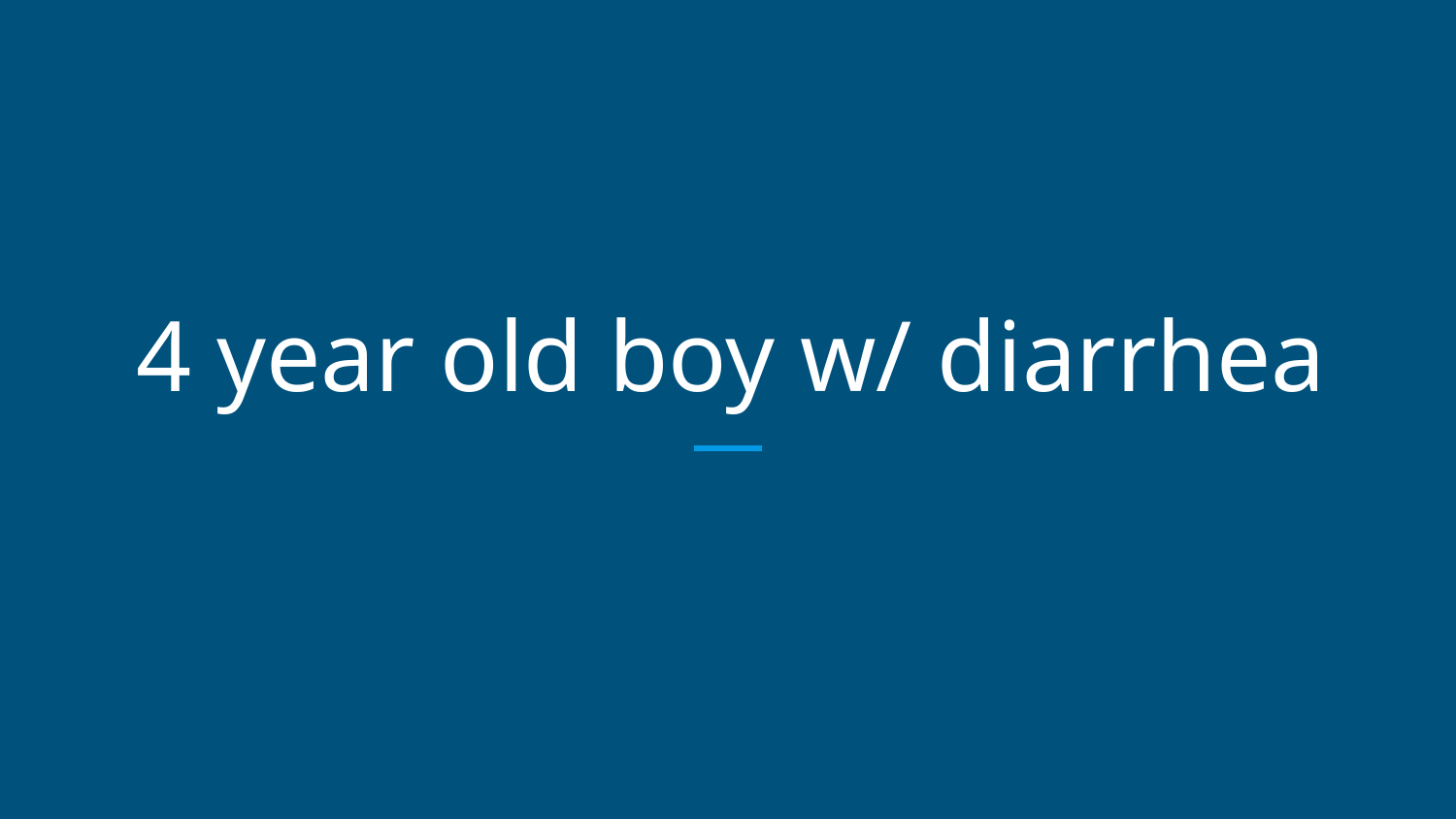

# 4 year old boy w/ diarrhea

## Slide 4
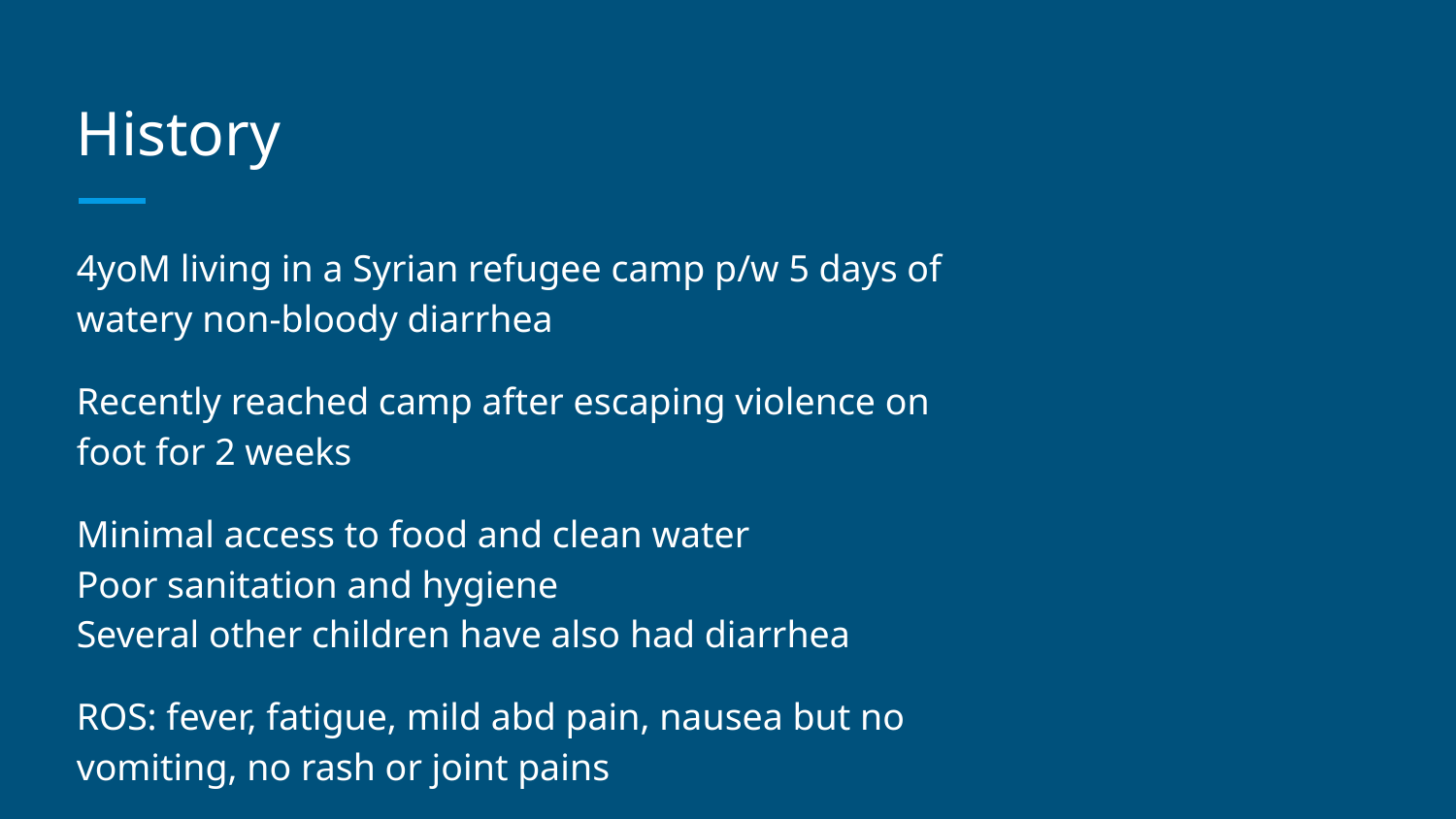

# History
4yoM living in a Syrian refugee camp p/w 5 days of watery non-bloody diarrhea
Recently reached camp after escaping violence on foot for 2 weeks
Minimal access to food and clean water
Poor sanitation and hygiene
Several other children have also had diarrhea
ROS: fever, fatigue, mild abd pain, nausea but no vomiting, no rash or joint pains

## Slide 5
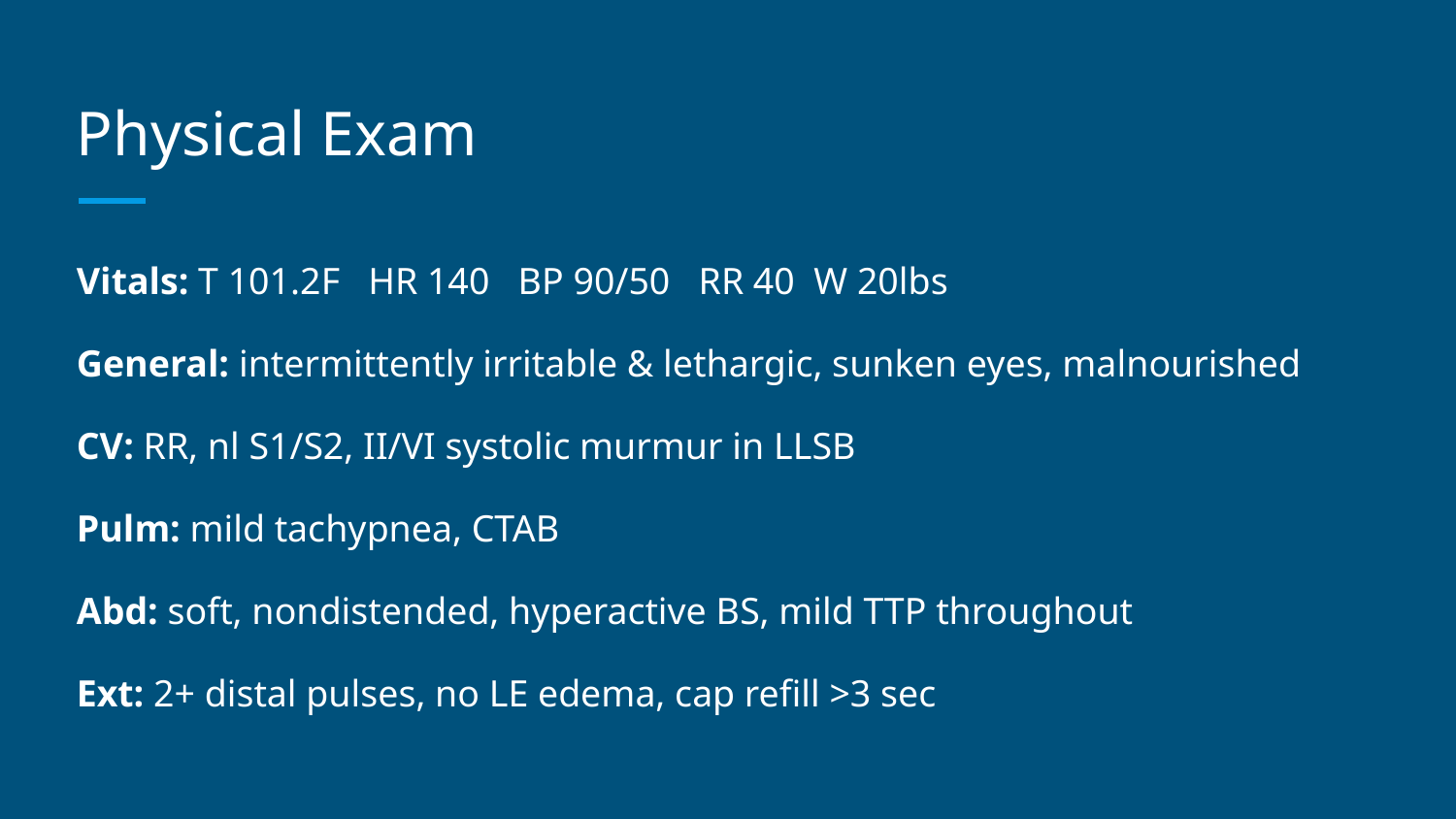

# Physical Exam
Vitals: T 101.2F HR 140 BP 90/50 RR 40 W 20lbs
General: intermittently irritable & lethargic, sunken eyes, malnourished
CV: RR, nl S1/S2, II/VI systolic murmur in LLSB
Pulm: mild tachypnea, CTAB
Abd: soft, nondistended, hyperactive BS, mild TTP throughout
Ext: 2+ distal pulses, no LE edema, cap refill >3 sec

## Slide 6
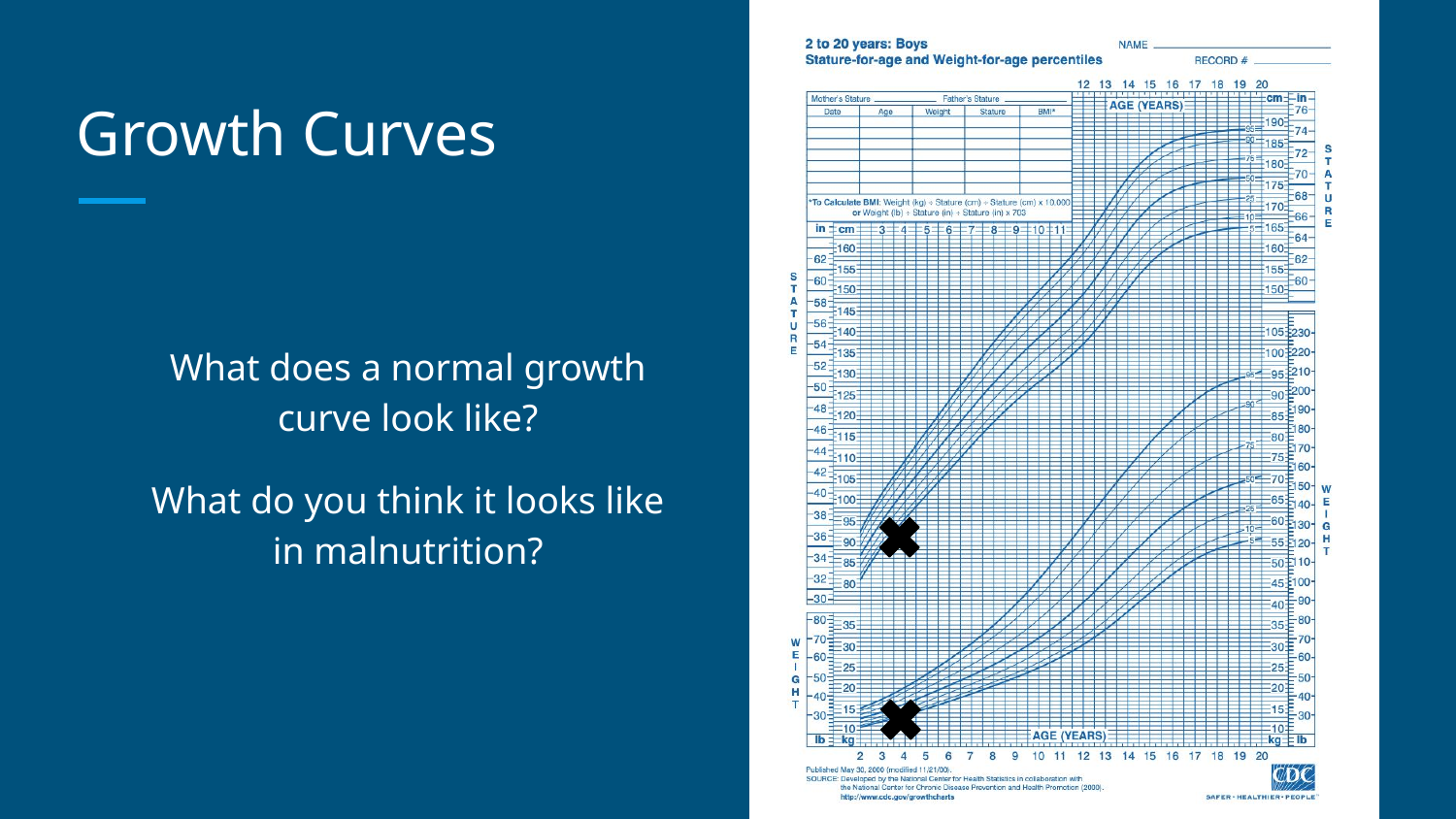

# Growth Curves
What does a normal growth curve look like?
What do you think it looks like in malnutrition?

## Slide 7
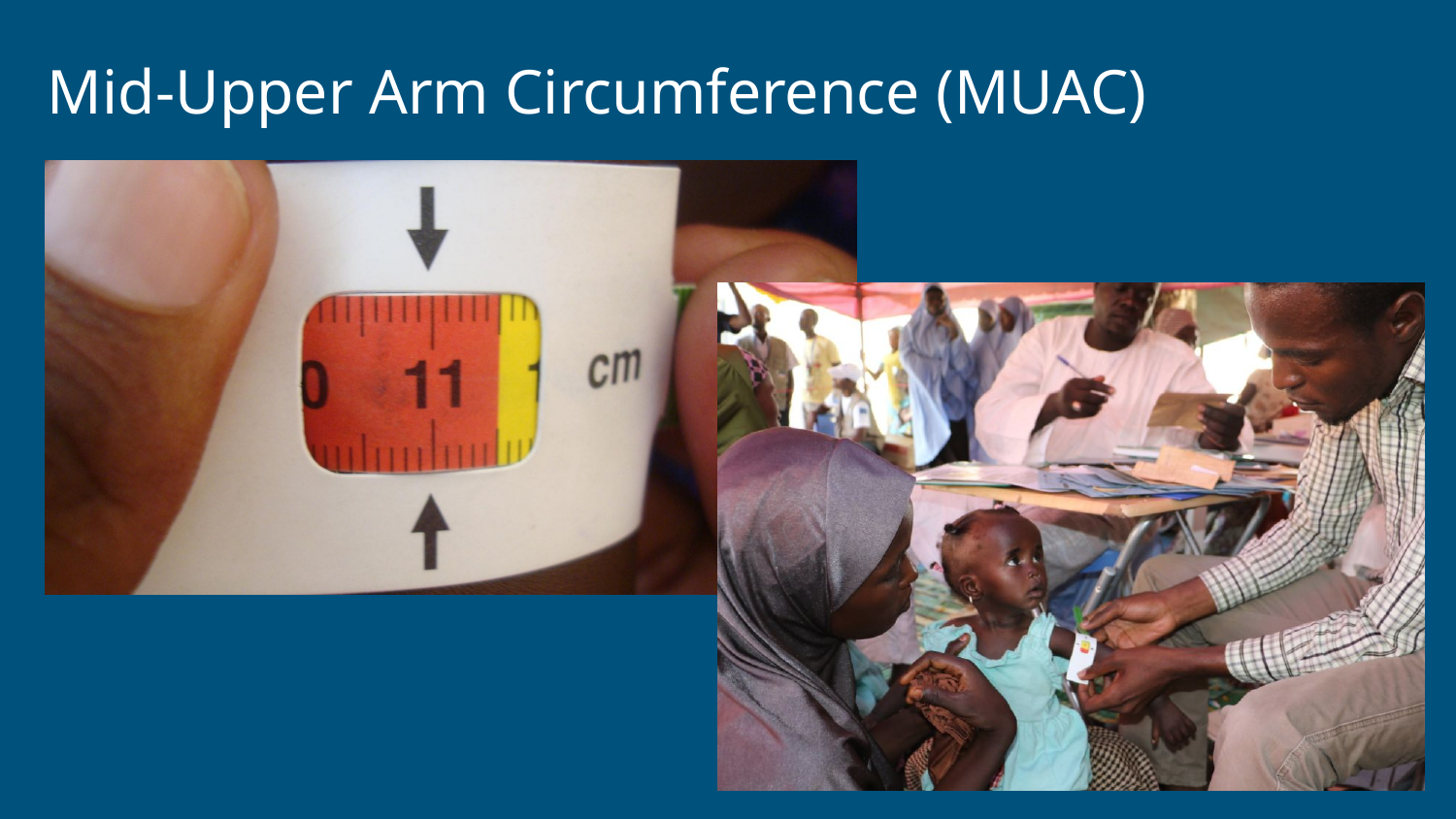

# Mid-Upper Arm Circumference (MUAC)

## Slide 8
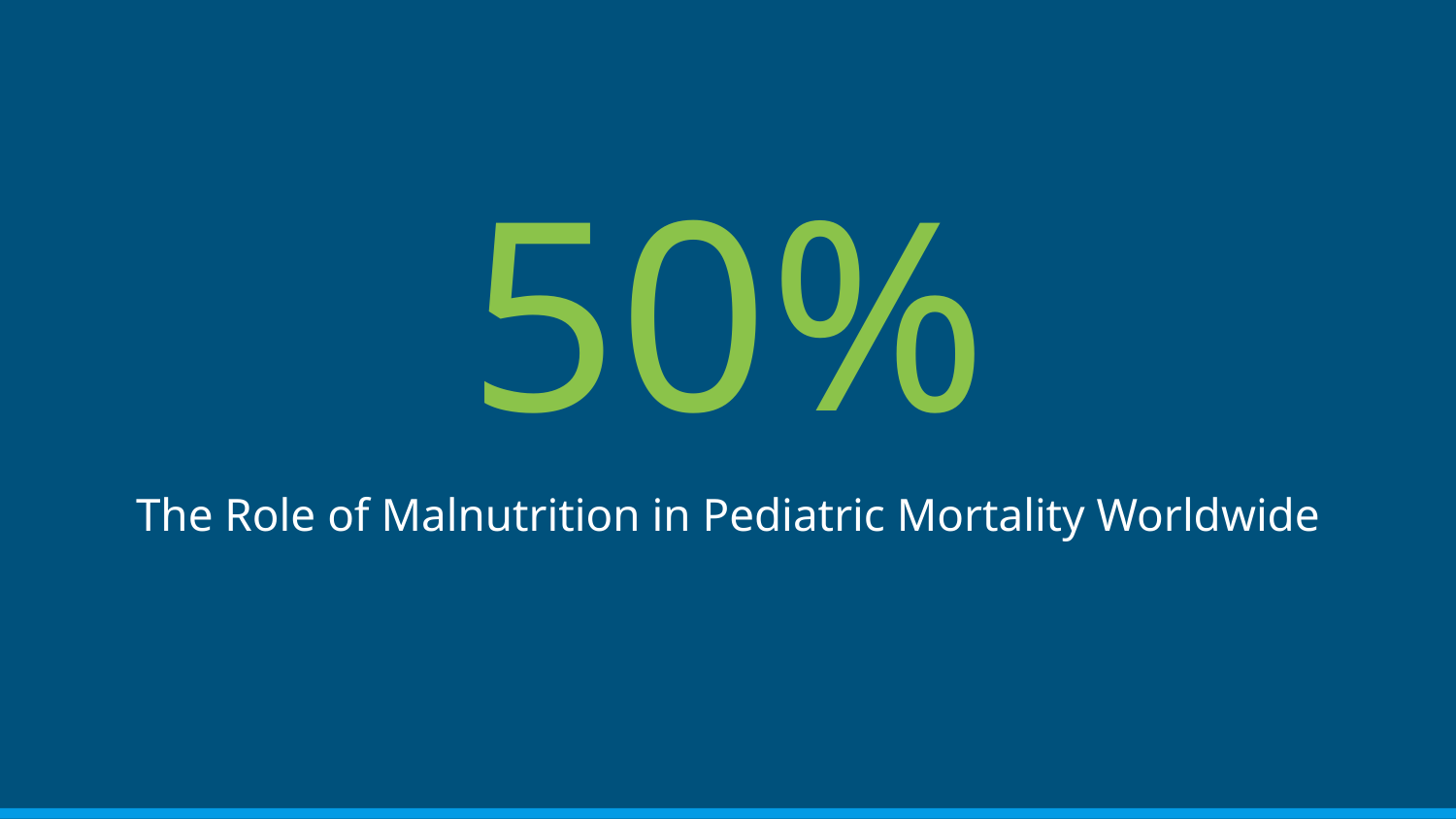

# 50%
The Role of Malnutrition in Pediatric Mortality Worldwide

## Slide 9
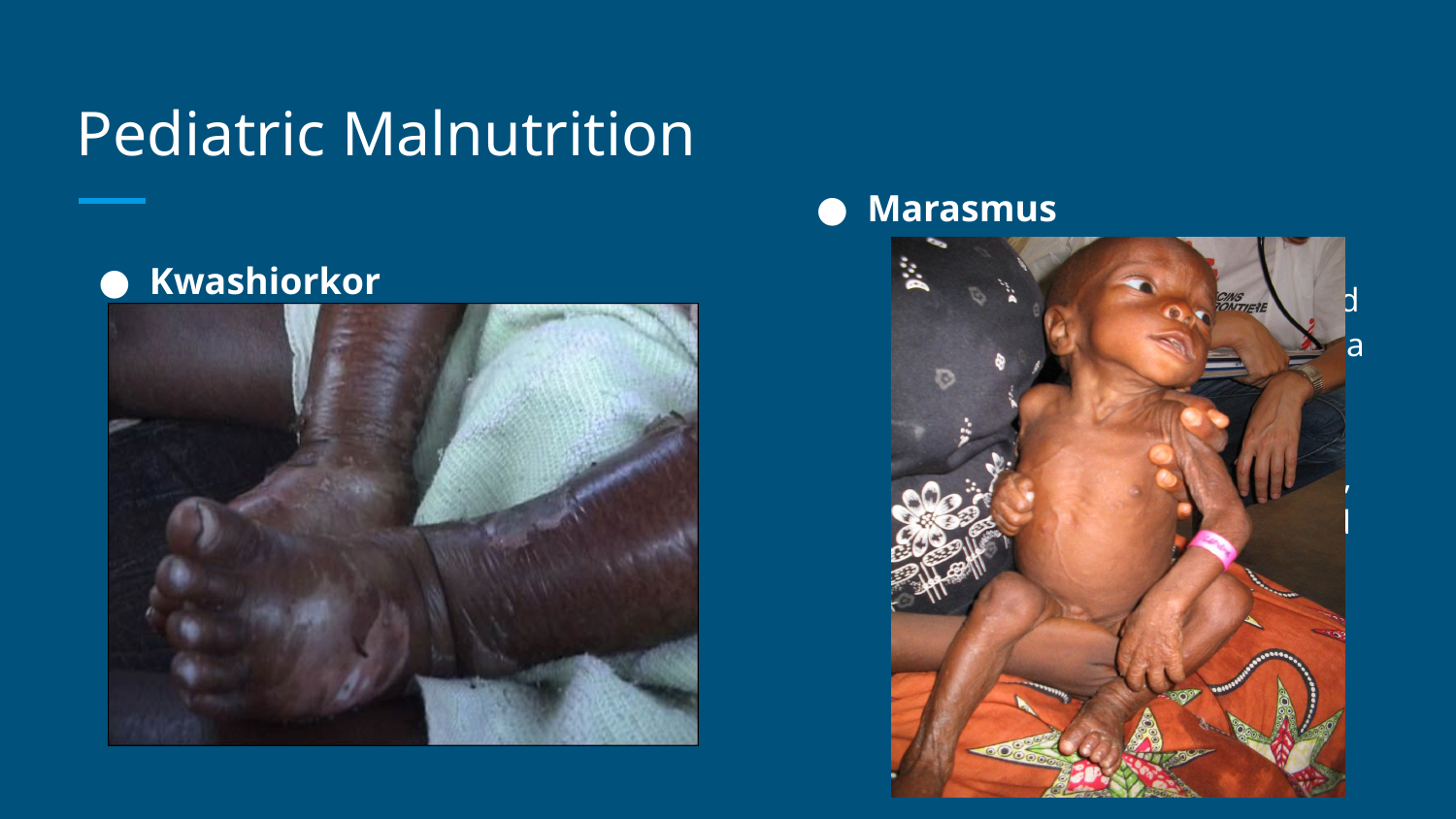

# Pediatric Malnutrition
Marasmus
Low energy diets, relative protein sufficiency, adapted protein metabolism, plasma proteins maintained
Adaptive state with fat and muscle loss, easier to treat, and low mortality if treated
Kwashiorkor
Very low protein diets, relative energy sufficiency, poorly adapted protein metabolism, low plasma protein, amino acid deficiency, liver damage
Maladaptive state characterized by edema, apathy, skin lesions, hepatic dysfunction
Hard to treat with high mortality rates

## Slide 10
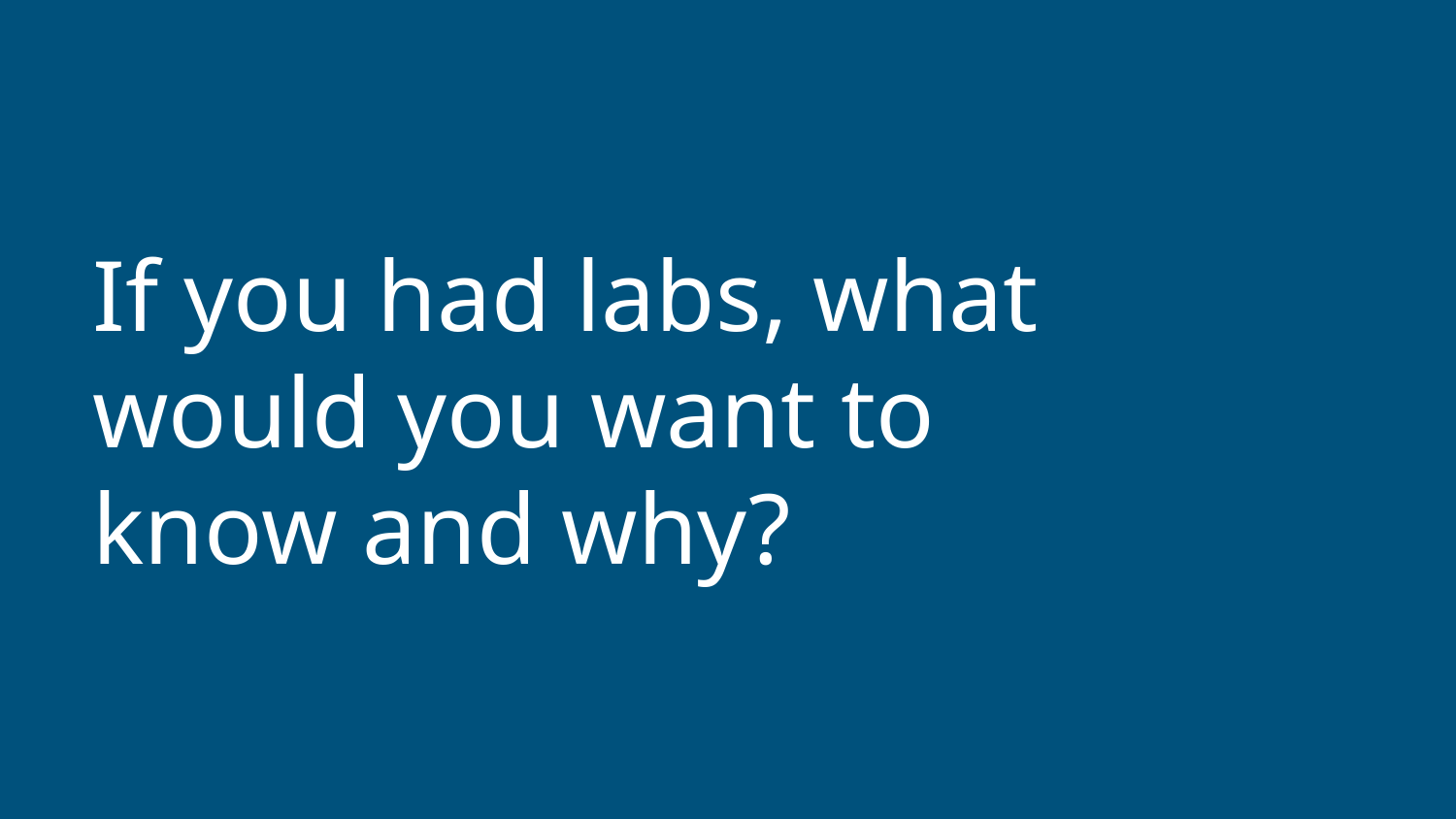

# If you had labs, what would you want to know and why?

## Slide 11
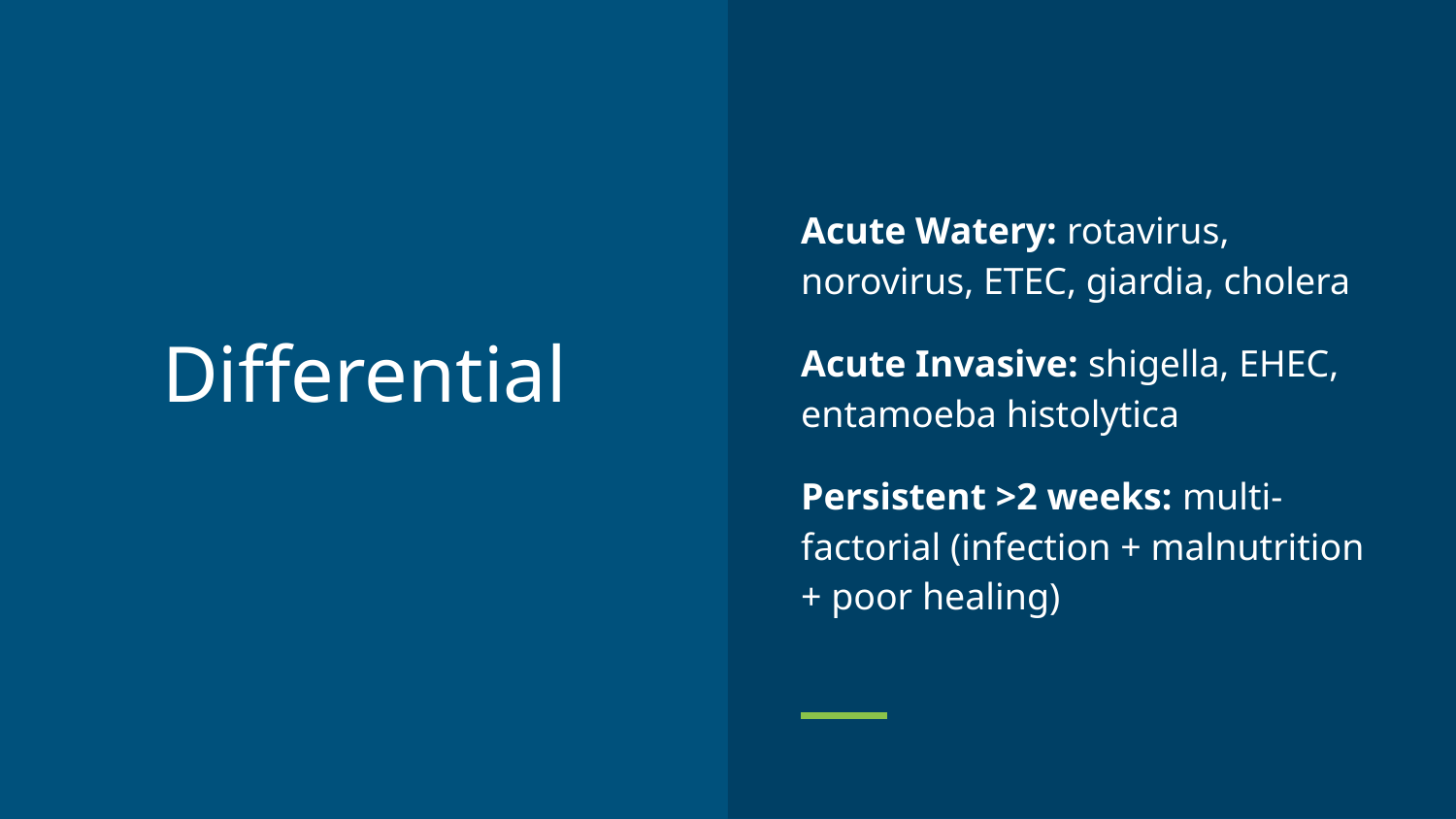

Acute Watery: rotavirus, norovirus, ETEC, giardia, cholera
Acute Invasive: shigella, EHEC, entamoeba histolytica
Persistent >2 weeks: multi-factorial (infection + malnutrition + poor healing)
# Differential

## Slide 12
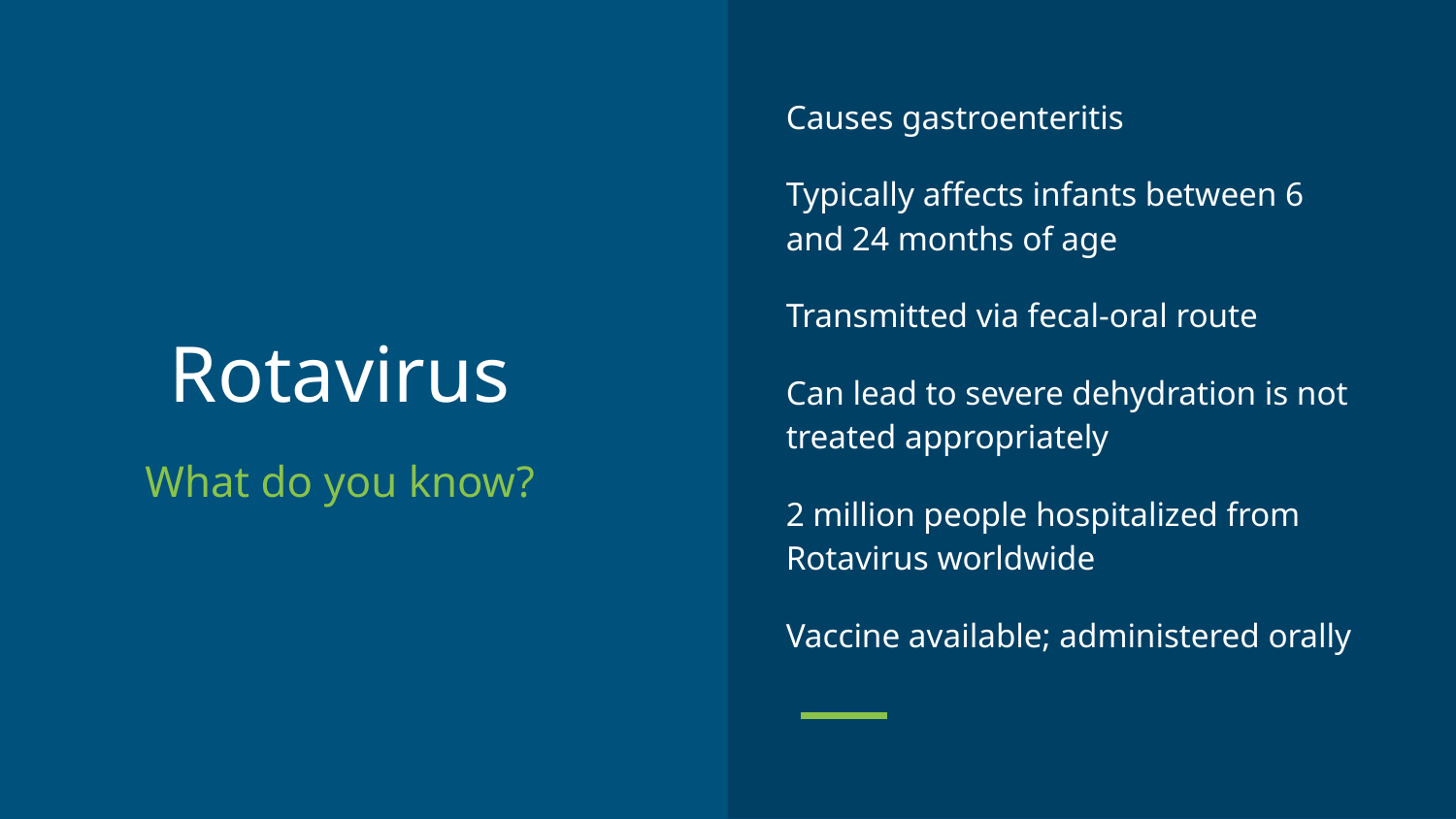

Causes gastroenteritis
Typically affects infants between 6 and 24 months of age
Transmitted via fecal-oral route
Can lead to severe dehydration is not treated appropriately
2 million people hospitalized from Rotavirus worldwide
Vaccine available; administered orally
# Rotavirus
What do you know?

## Slide 13
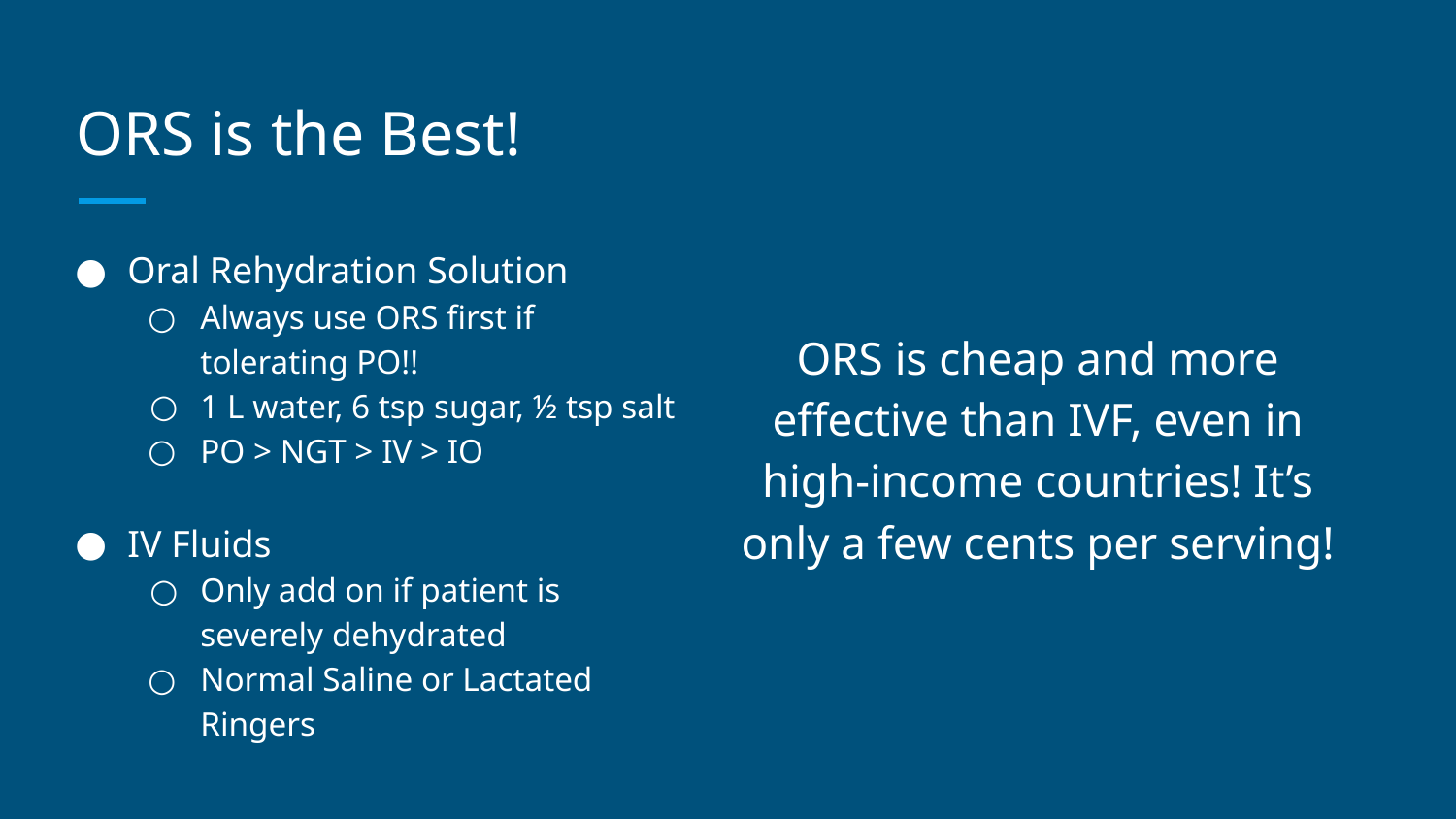

# ORS is the Best!
Oral Rehydration Solution
Always use ORS first if tolerating PO!!
1 L water, 6 tsp sugar, ½ tsp salt
PO > NGT > IV > IO
IV Fluids
Only add on if patient is severely dehydrated
Normal Saline or Lactated Ringers
ORS is cheap and more effective than IVF, even in high-income countries! It’s only a few cents per serving!

## Slide 14
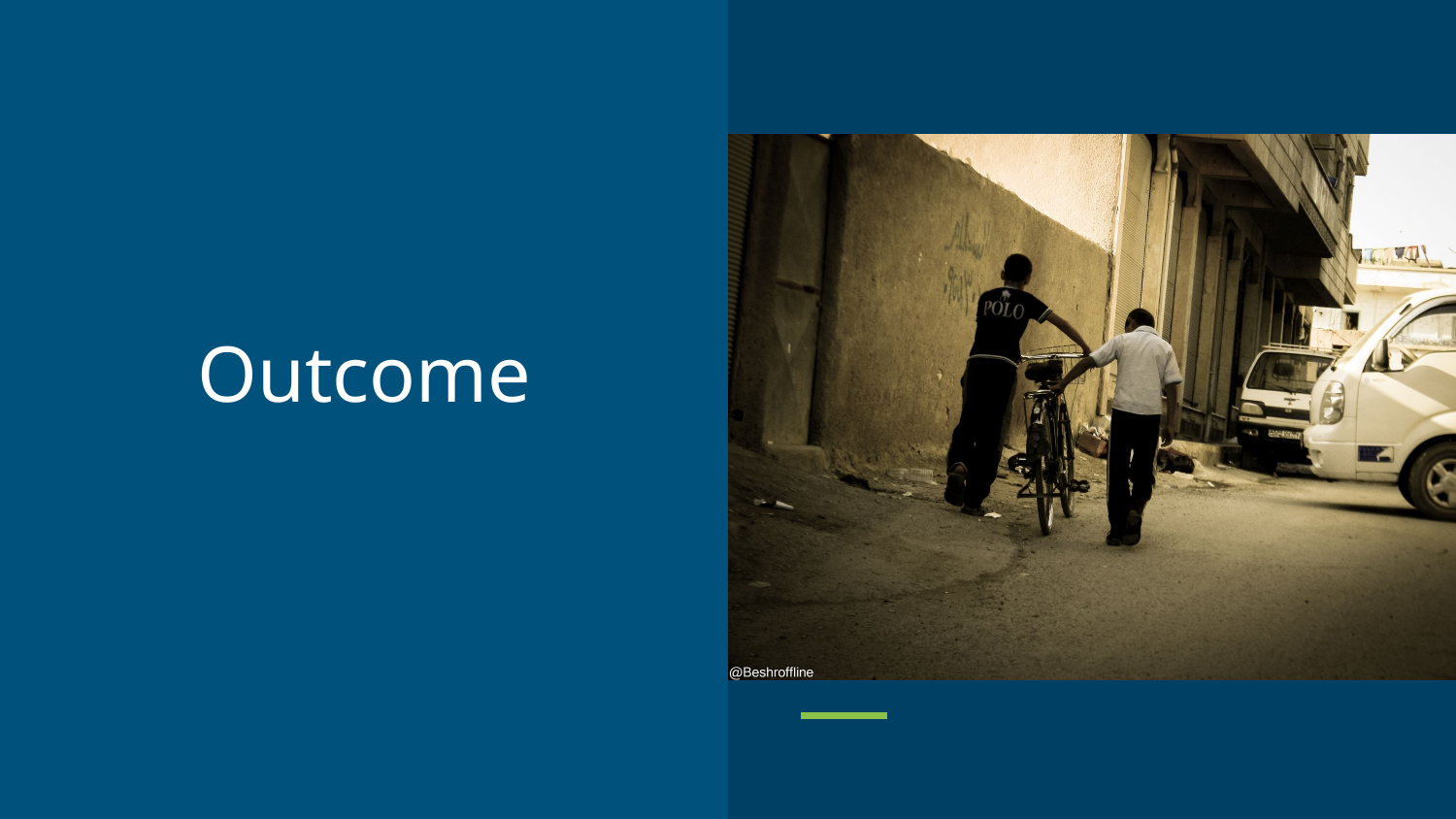

# Outcome

## Slide 15
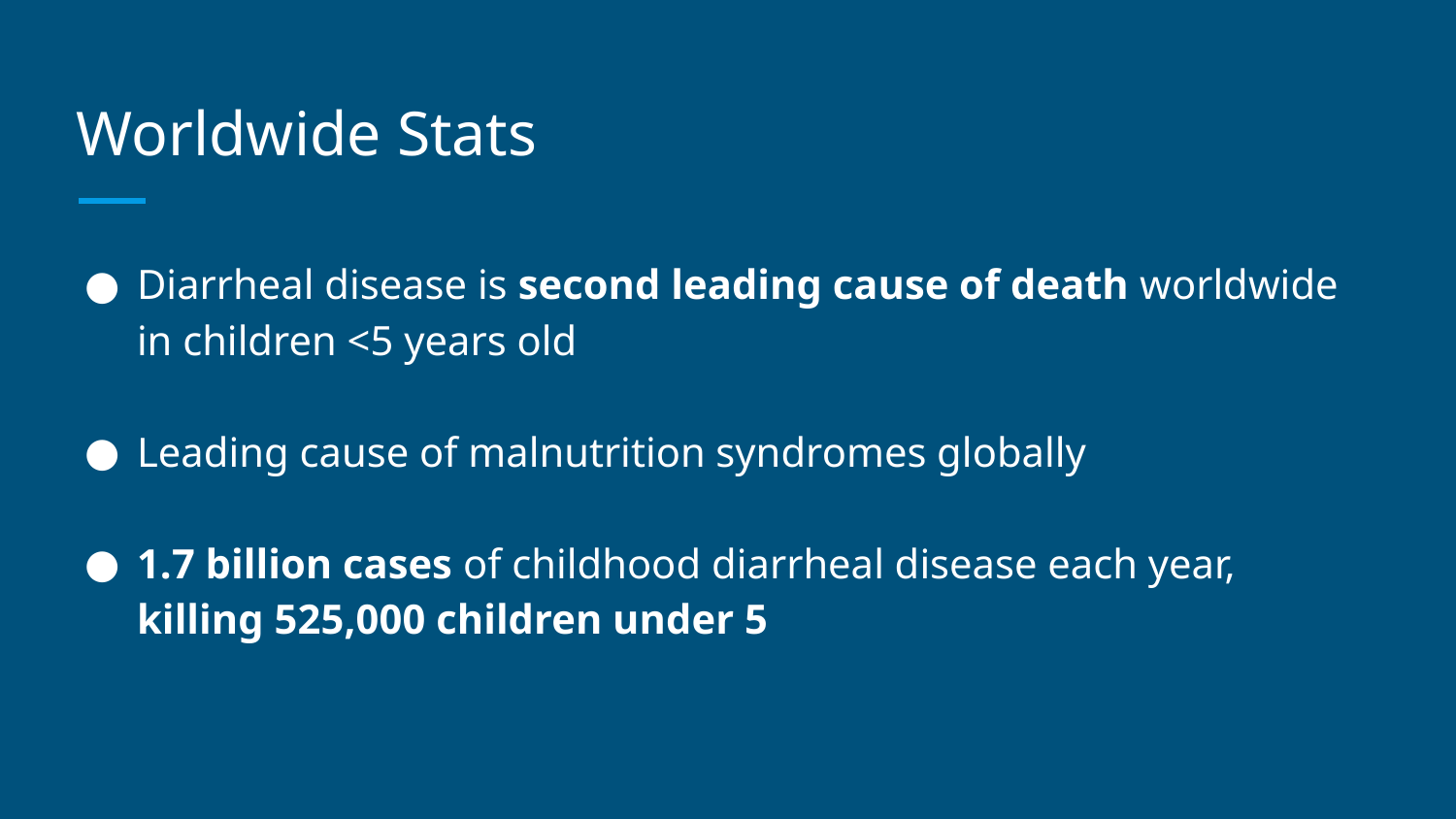

# Worldwide Stats
Diarrheal disease is second leading cause of death worldwide in children <5 years old
Leading cause of malnutrition syndromes globally
1.7 billion cases of childhood diarrheal disease each year, killing 525,000 children under 5

## Slide 16
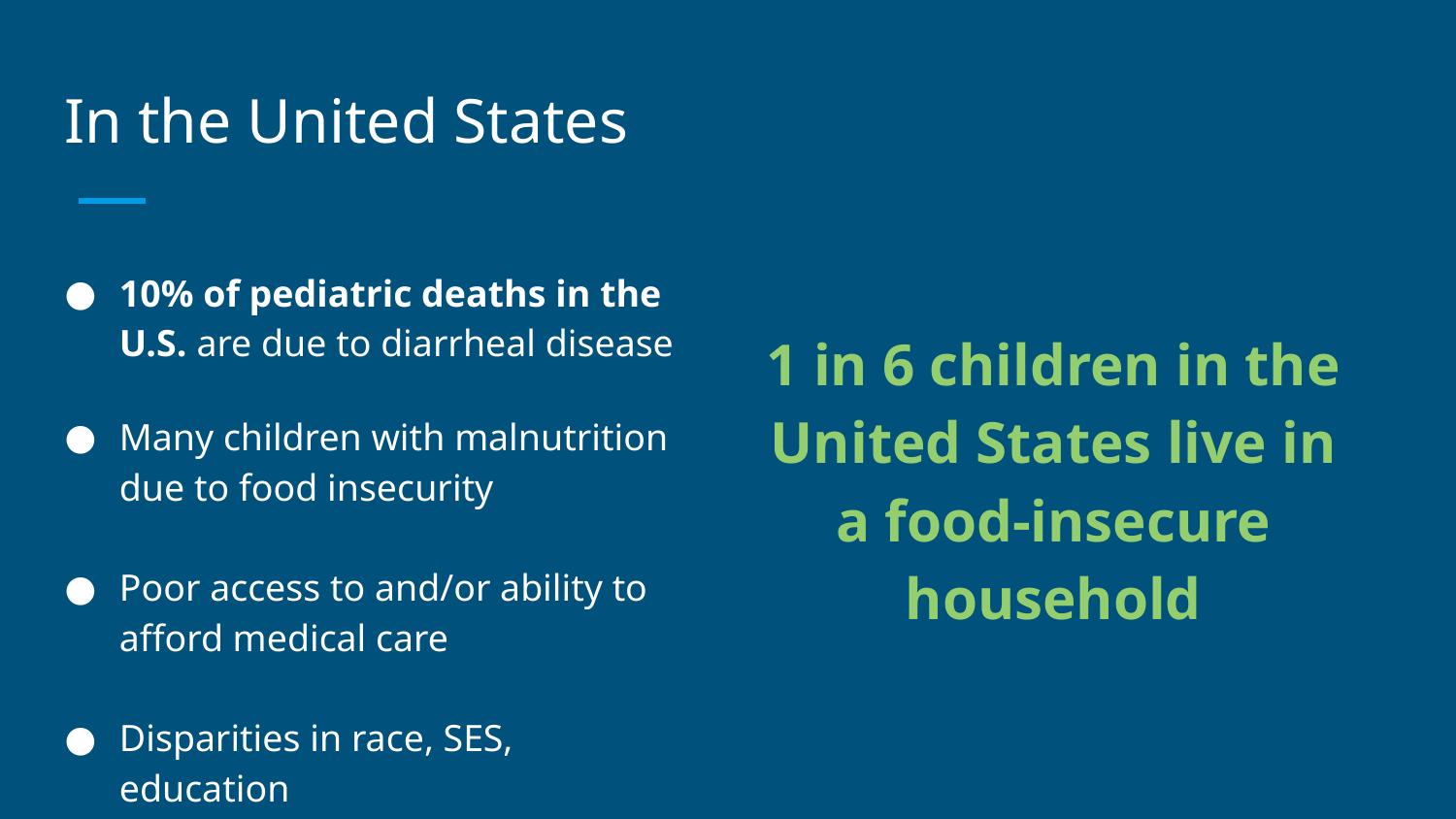

# In the United States
10% of pediatric deaths in the U.S. are due to diarrheal disease
Many children with malnutrition due to food insecurity
Poor access to and/or ability to afford medical care
Disparities in race, SES, education
1 in 6 children in the United States live in a food-insecure household

## Slide 17
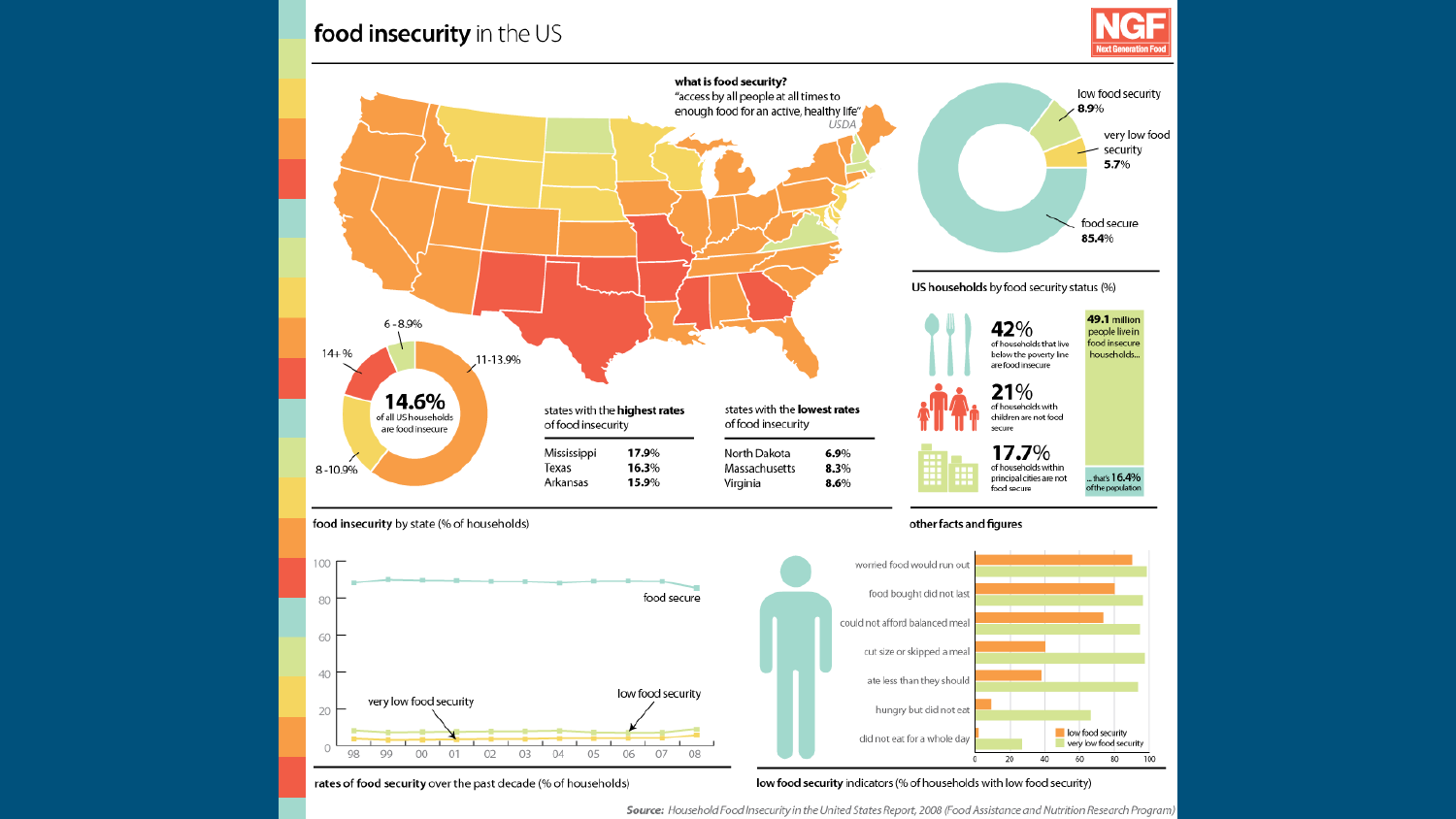

## Slide 18
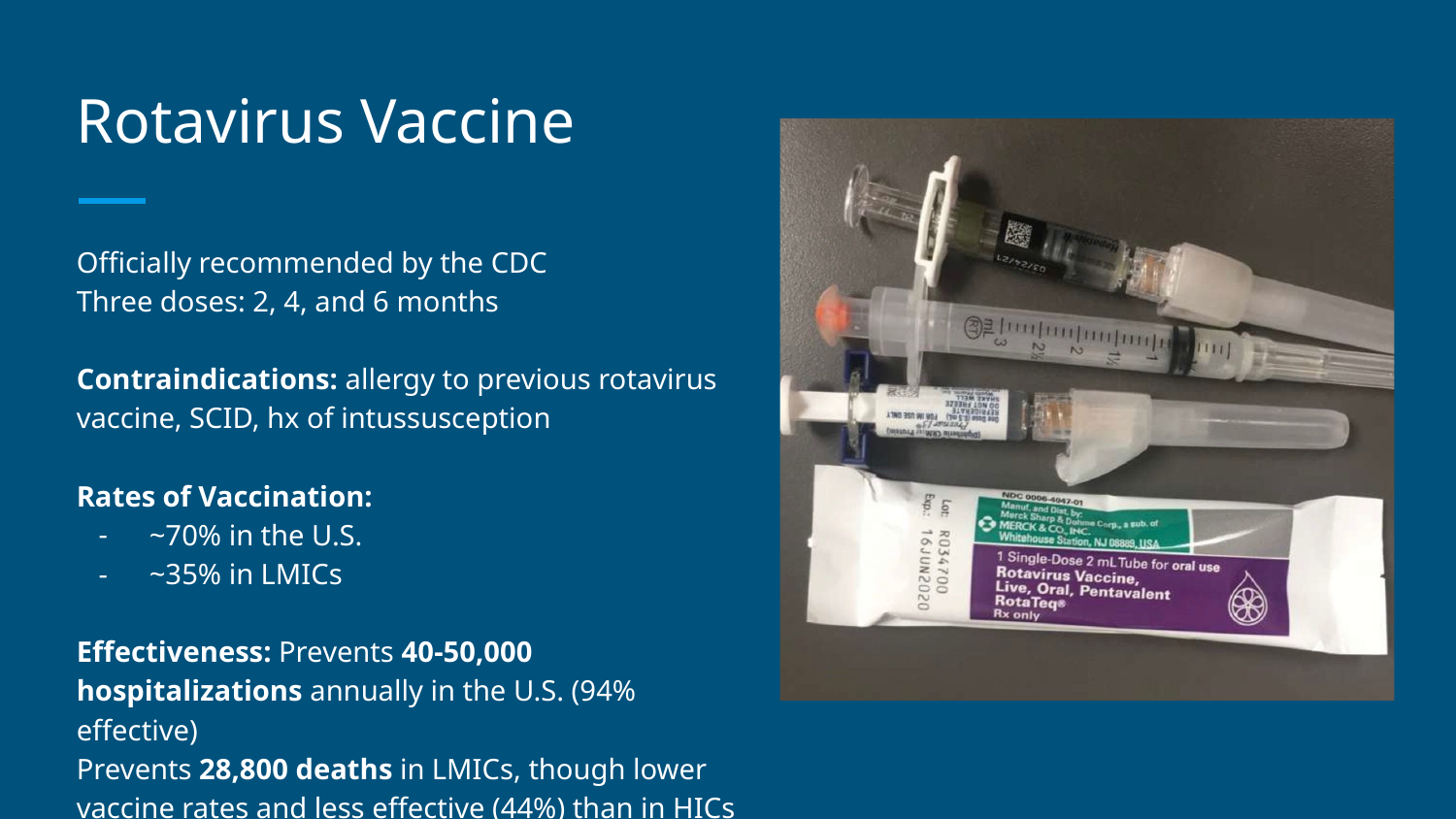

# Rotavirus Vaccine
Officially recommended by the CDC
Three doses: 2, 4, and 6 months
Contraindications: allergy to previous rotavirus vaccine, SCID, hx of intussusception
Rates of Vaccination:
~70% in the U.S.
~35% in LMICs
Effectiveness: Prevents 40-50,000 hospitalizations annually in the U.S. (94% effective)
Prevents 28,800 deaths in LMICs, though lower vaccine rates and less effective (44%) than in HICs

## Slide 19
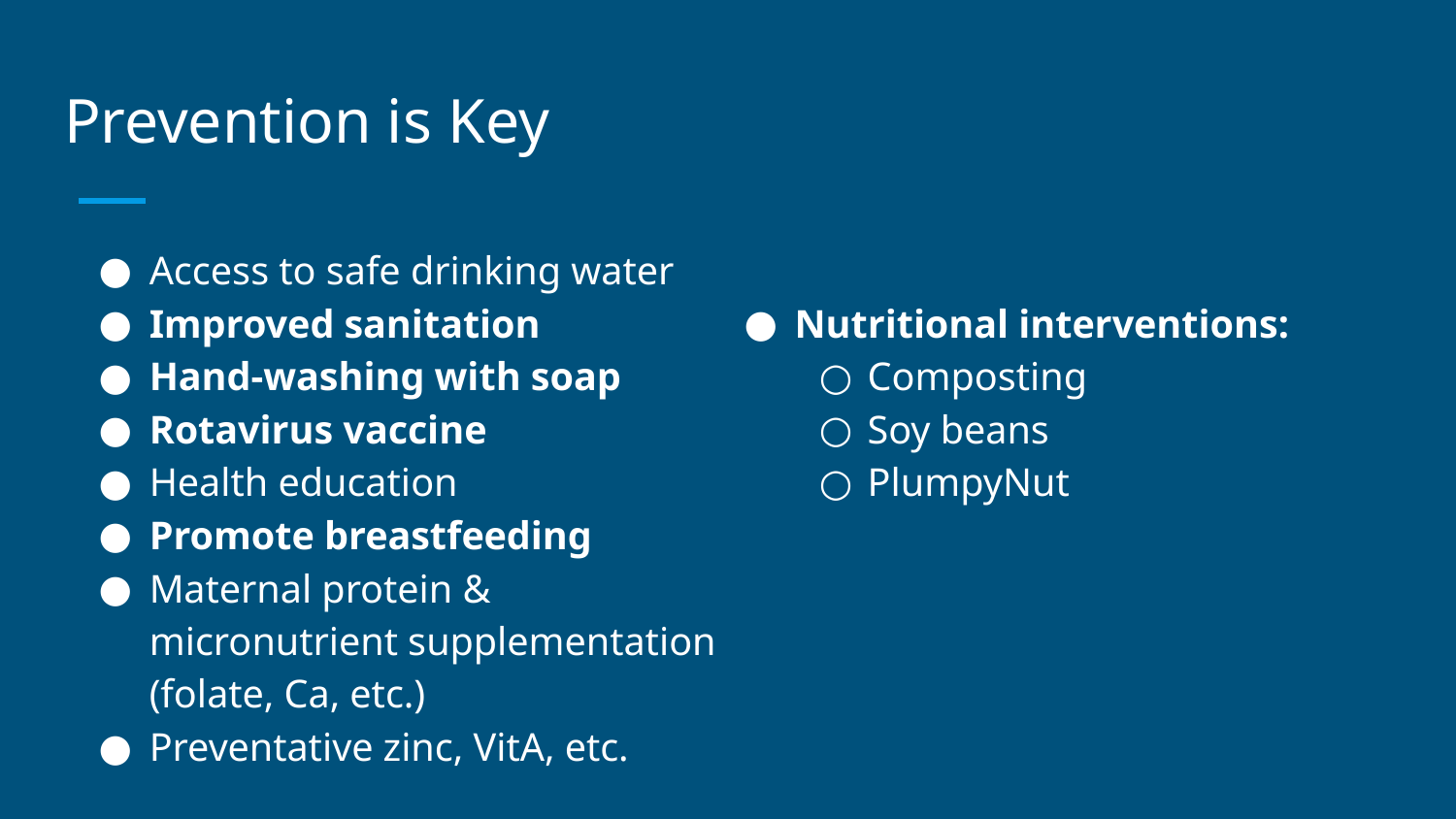

# Prevention is Key
Access to safe drinking water
Improved sanitation
Hand-washing with soap
Rotavirus vaccine
Health education
Promote breastfeeding
Maternal protein & micronutrient supplementation (folate, Ca, etc.)
Preventative zinc, VitA, etc.
Nutritional interventions:
Composting
Soy beans
PlumpyNut

## Slide 20
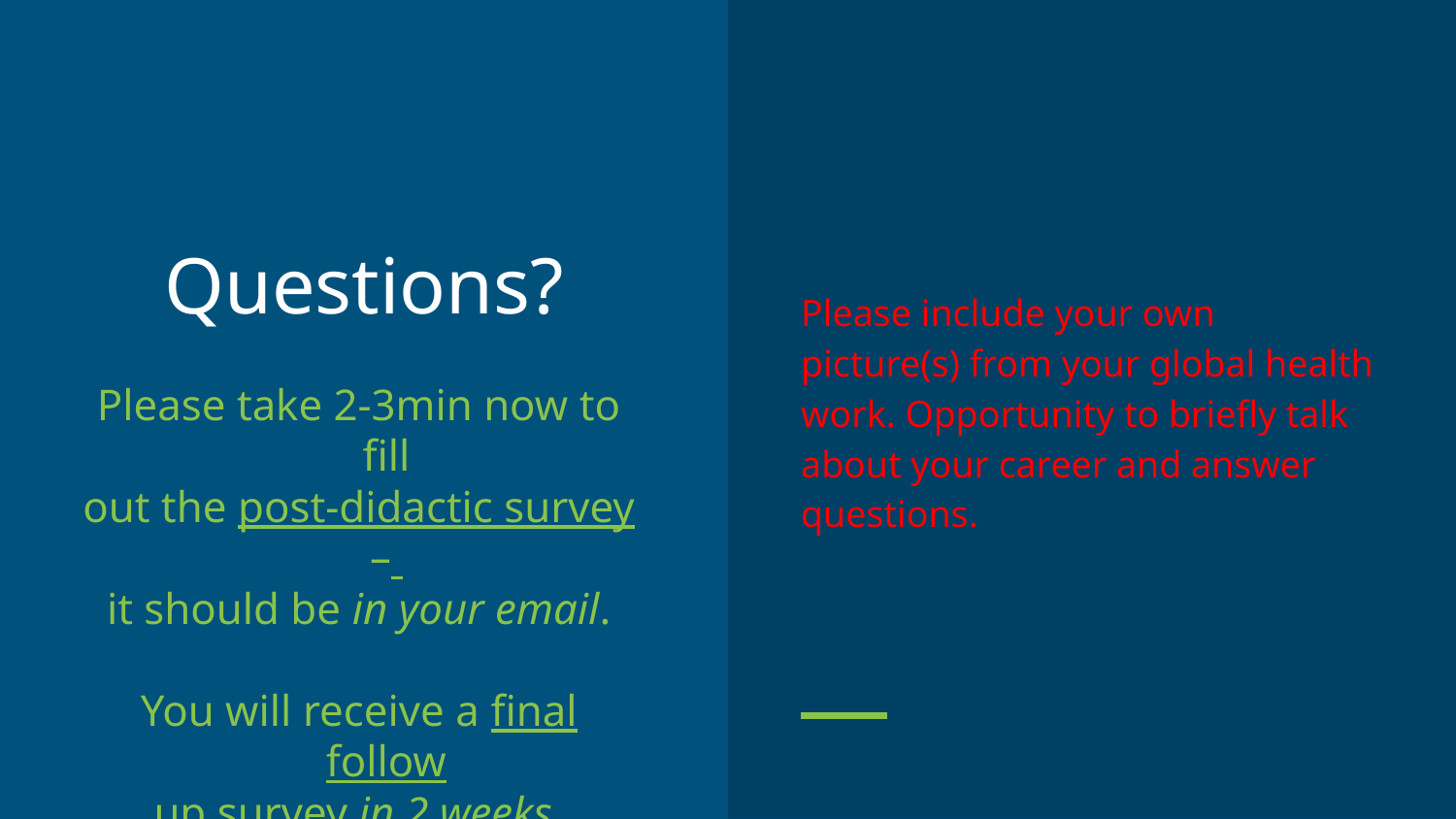

# Questions?
Please include your own picture(s) from your global health work. Opportunity to briefly talk about your career and answer questions.
Please take 2-3min now to fill
out the post-didactic survey –
it should be in your email.
You will receive a final follow
up survey in 2 weeks.
