# Supplementary material for: A Longitudinal Case-Based Global Health Curriculum for the Medical Student Clerkship Year
Source: MedEdPORTAL. 2020 Dec 8;16:11038. doi: 10.15766/mep_2374-8265.11038 (PMC7732136; doi:10.15766/mep_2374-8265.11038)
Supplement: Supplementary file 1 — Clerkship Director Proposal.pptxProject Description.docxPediatrics GH Didactic.pptxSurgery GH Didactic.pptxMedicine GH Didactic.pptxFacilitator Notes.docxPredidactic Survey.docxPostdidactic Survey.docxFollow-up Survey.docx [file mep_2374-8265.11038-s001.zip › D. Surgery GH Didactic.pptx]

## Slide 1
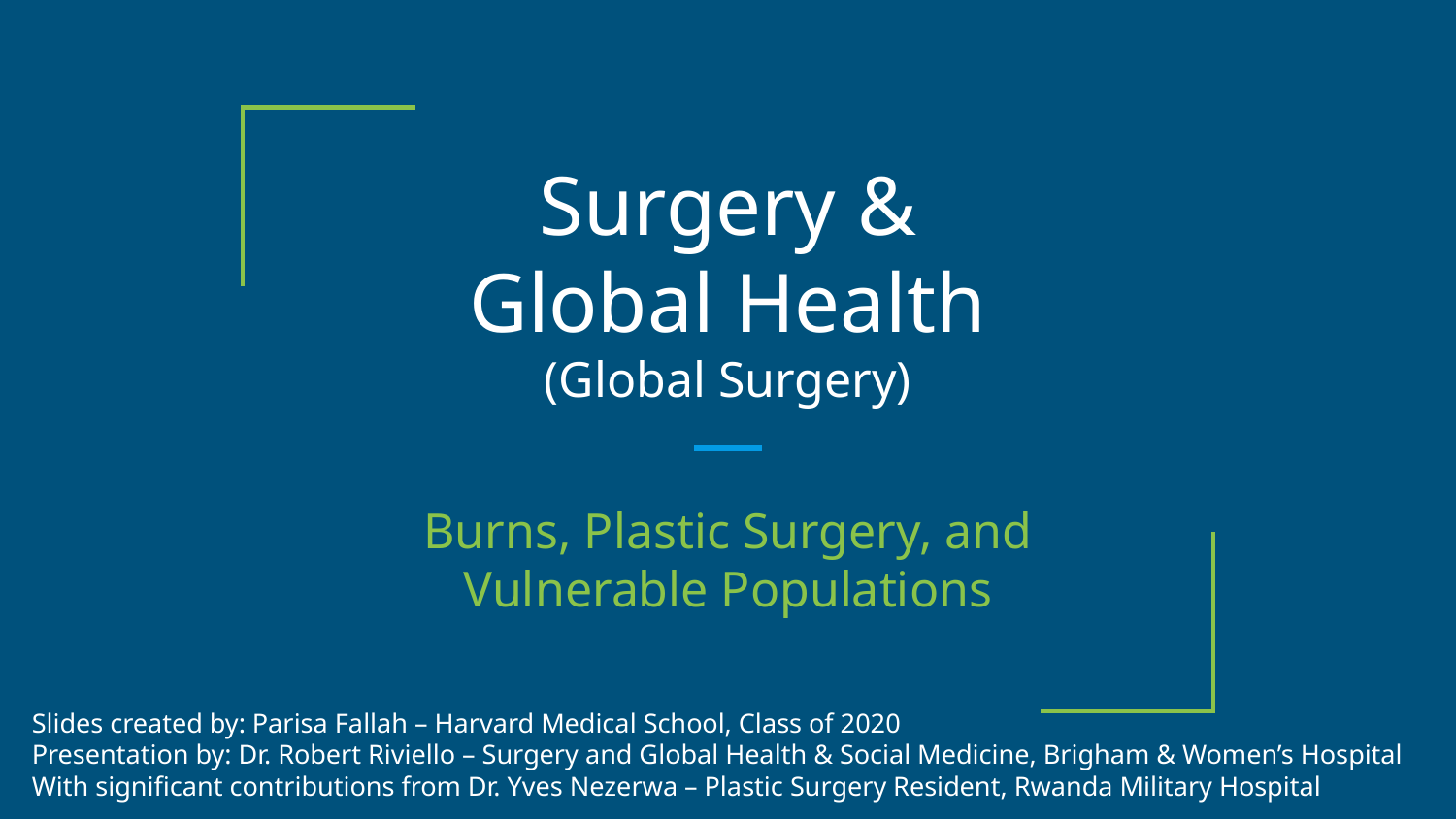

# Surgery & Global Health
(Global Surgery)
Burns, Plastic Surgery, and
Vulnerable Populations
Slides created by: Parisa Fallah – Harvard Medical School, Class of 2020
Presentation by: Dr. Robert Riviello – Surgery and Global Health & Social Medicine, Brigham & Women’s Hospital
With significant contributions from Dr. Yves Nezerwa – Plastic Surgery Resident, Rwanda Military Hospital

## Slide 2
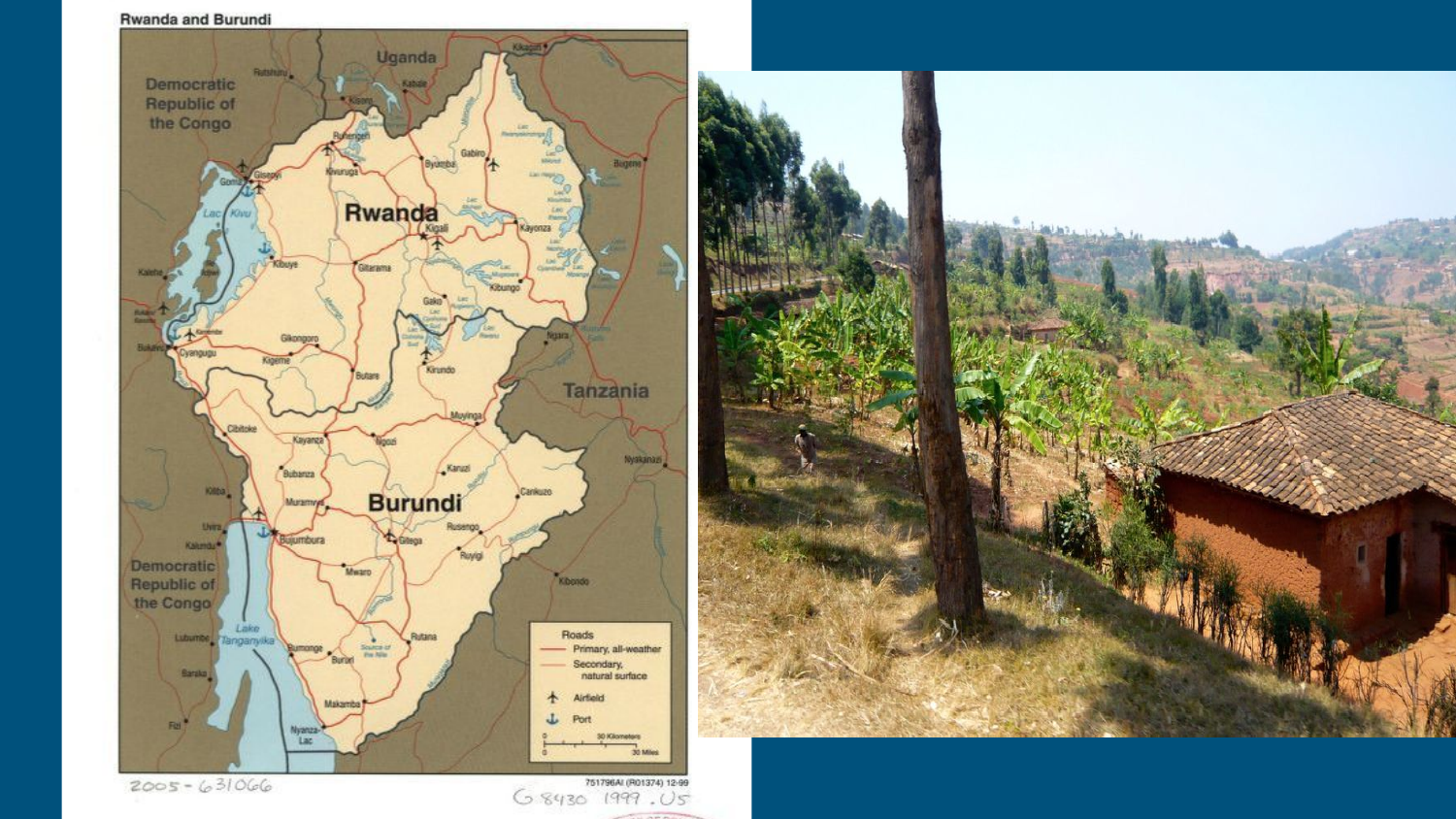

# Rural Rwandan Clinic
Working at a teaching hospital
in Kigali, Rwanda
Limited IV fluids
Unable to draw labs
Thermometers
Blood pressure cuff

## Slide 3
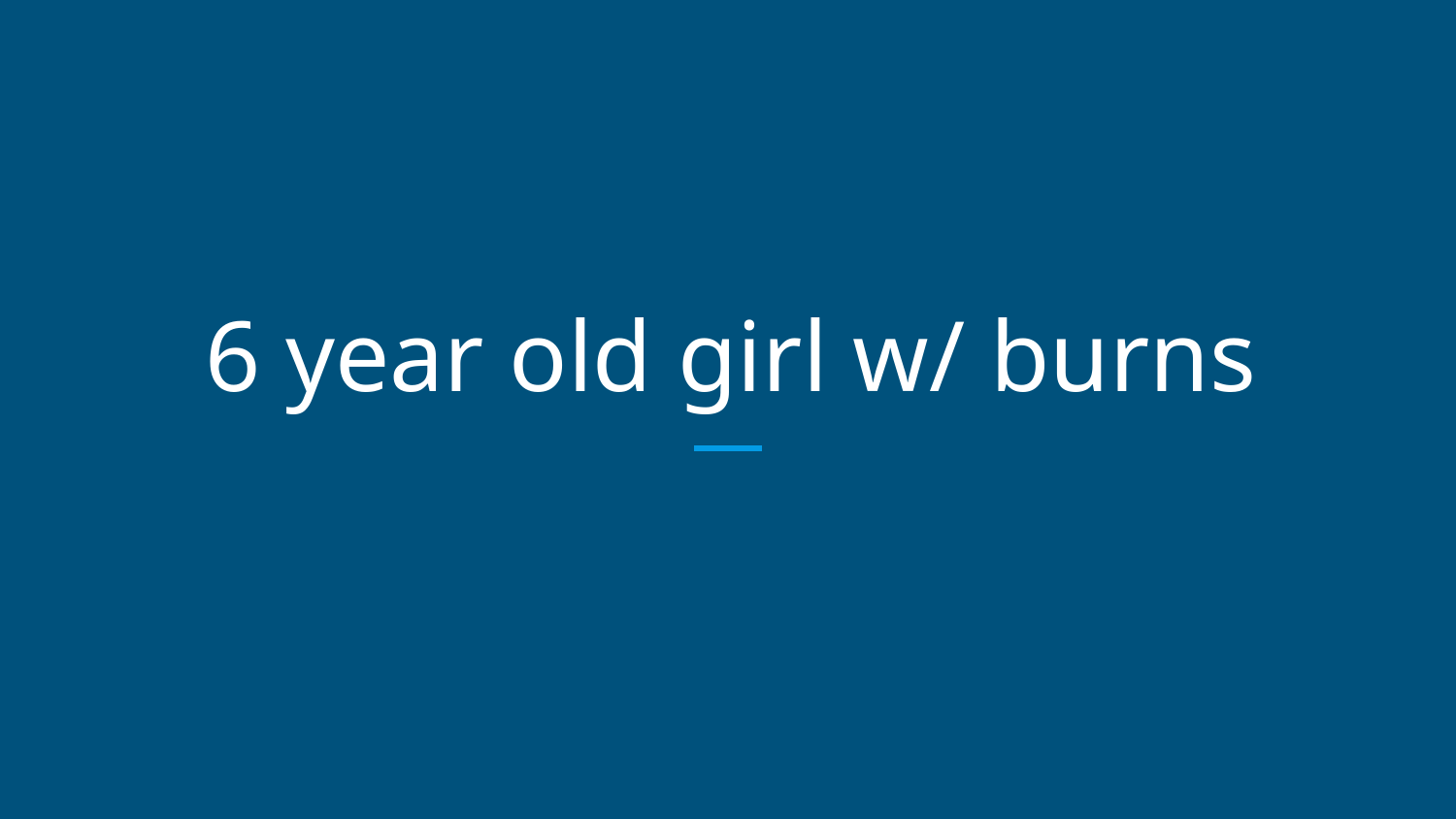

# 6 year old girl w/ burns

## Slide 4
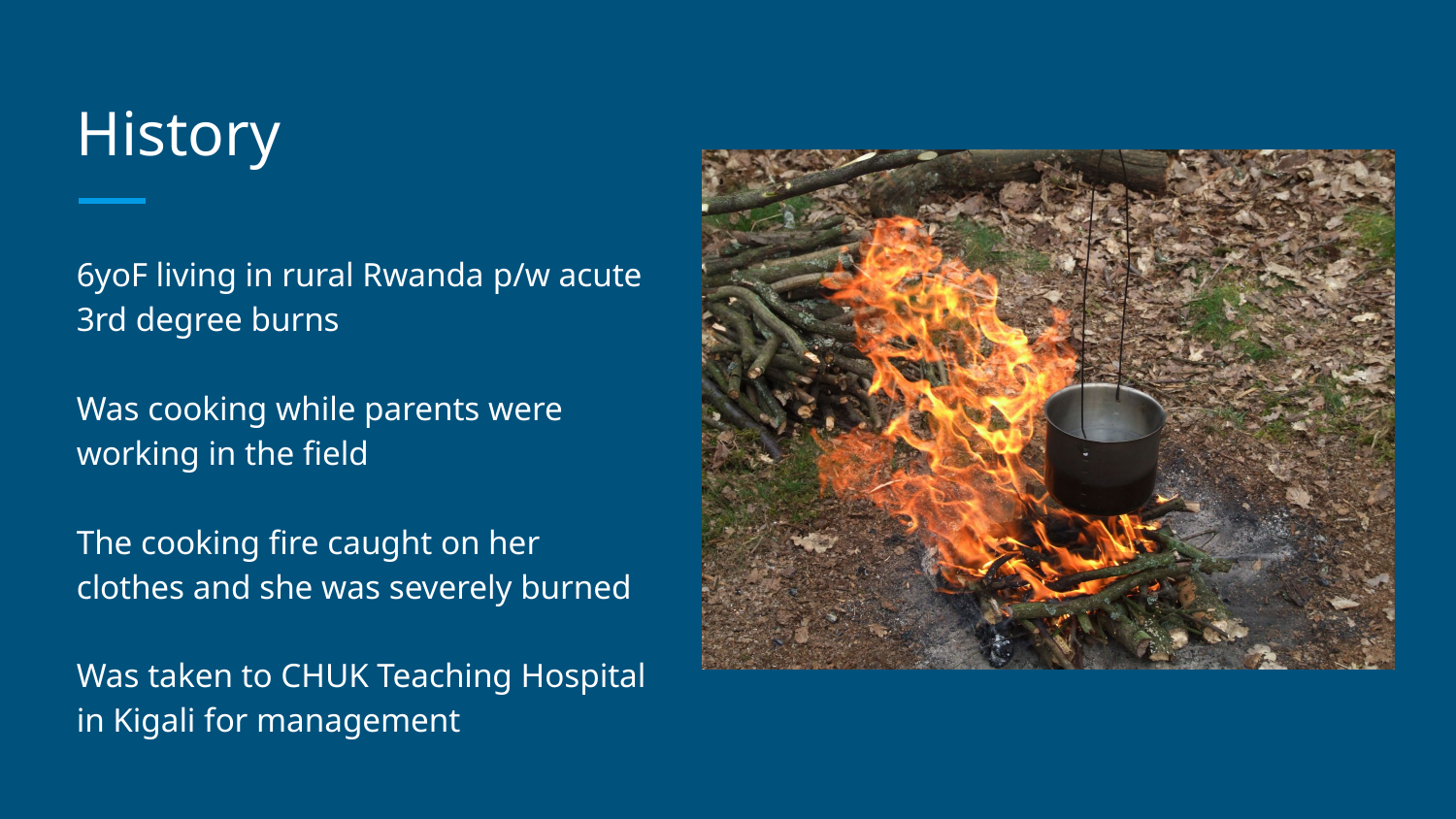

# History
6yoF living in rural Rwanda p/w acute 3rd degree burns
Was cooking while parents were working in the field
The cooking fire caught on her clothes and she was severely burned
Was taken to CHUK Teaching Hospital in Kigali for management

## Slide 5
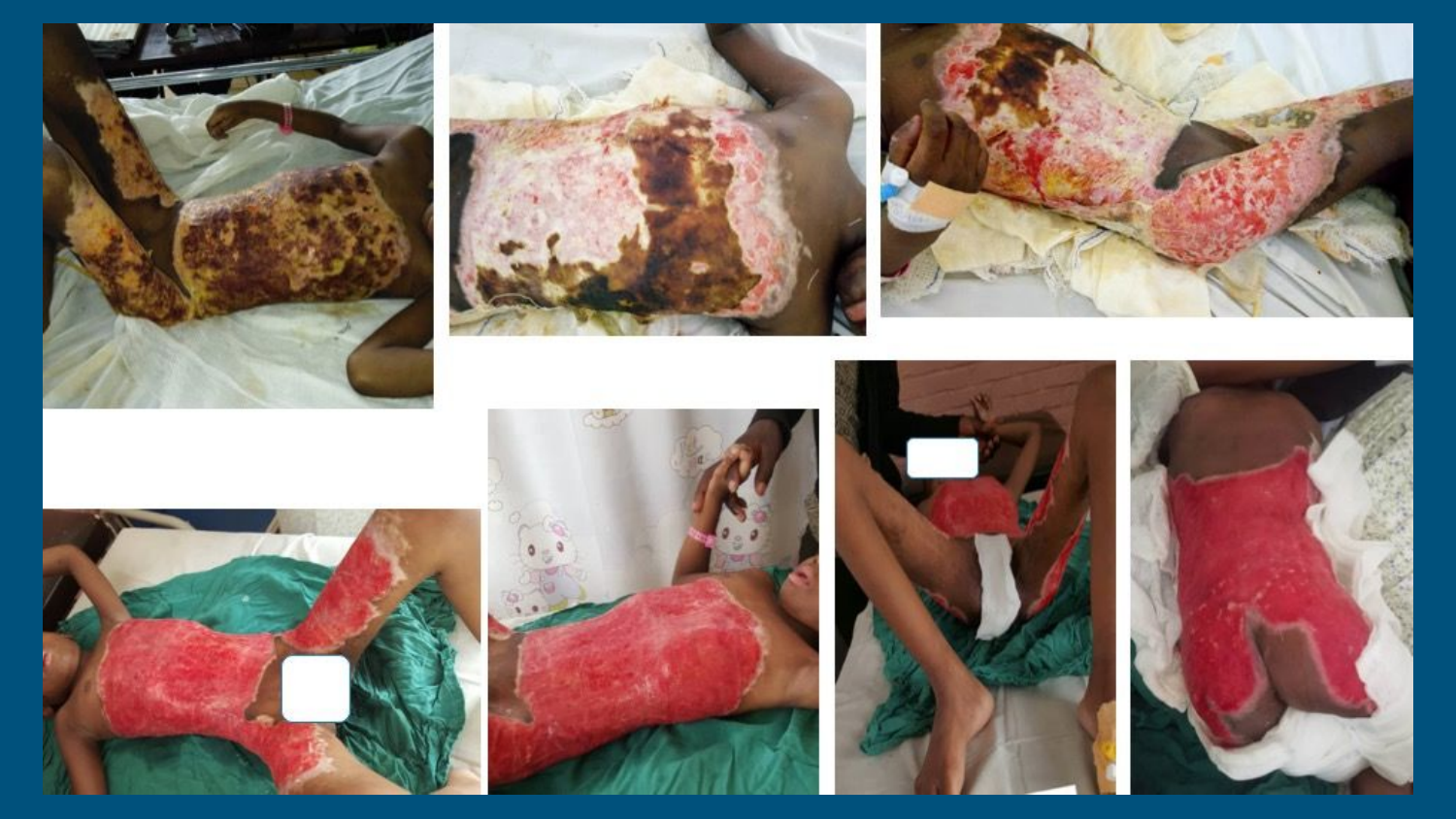

## Slide 6
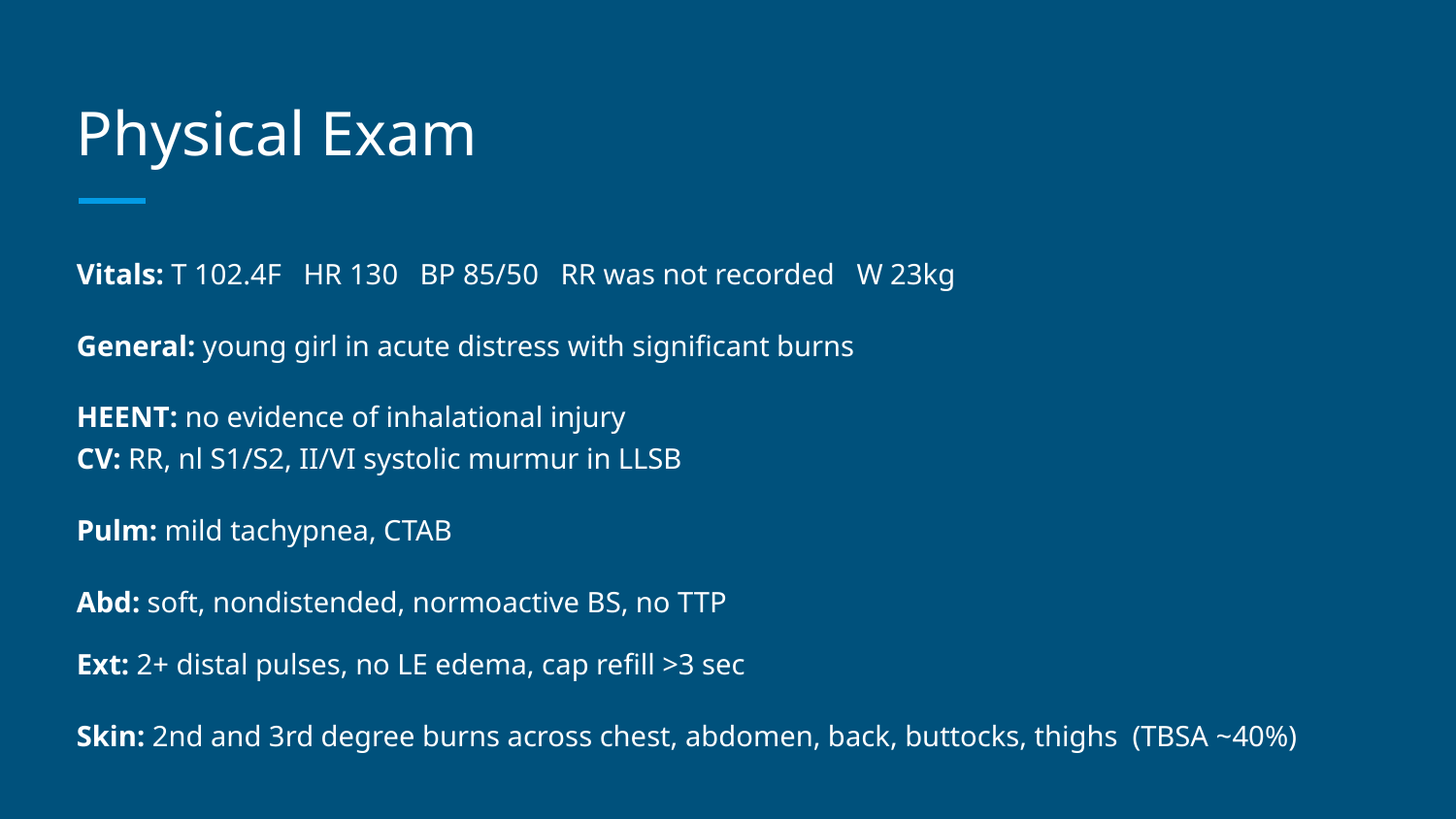

# Physical Exam
Vitals: T 102.4F HR 130 BP 85/50 RR was not recorded W 23kg
General: young girl in acute distress with significant burns
HEENT: no evidence of inhalational injury
CV: RR, nl S1/S2, II/VI systolic murmur in LLSB
Pulm: mild tachypnea, CTAB
Abd: soft, nondistended, normoactive BS, no TTP
Ext: 2+ distal pulses, no LE edema, cap refill >3 sec
Skin: 2nd and 3rd degree burns across chest, abdomen, back, buttocks, thighs (TBSA ~40%)

## Slide 7
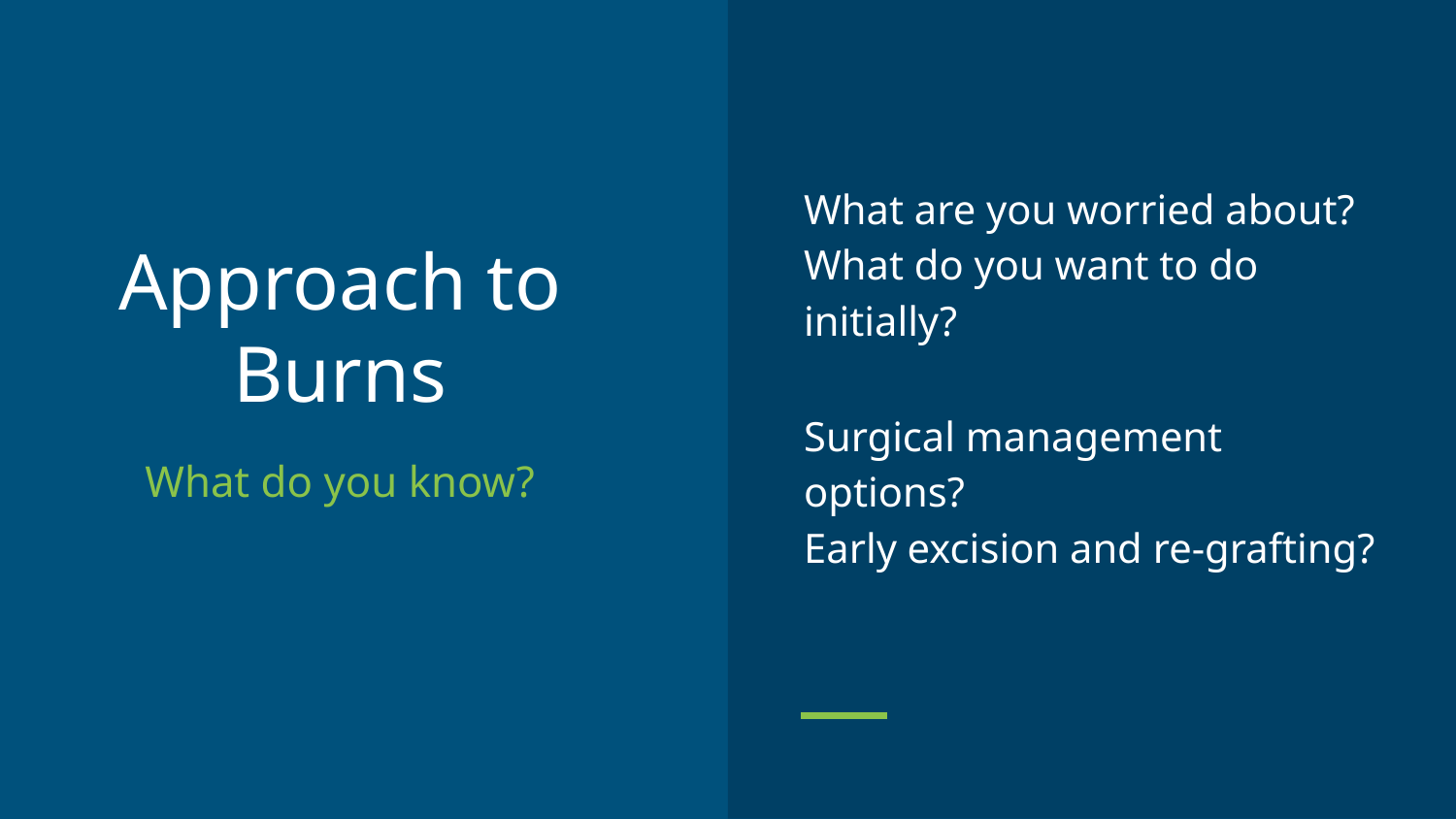

What are you worried about?
What do you want to do initially?
# Approach to Burns
Surgical management options?
Early excision and re-grafting?
What do you know?

## Slide 8
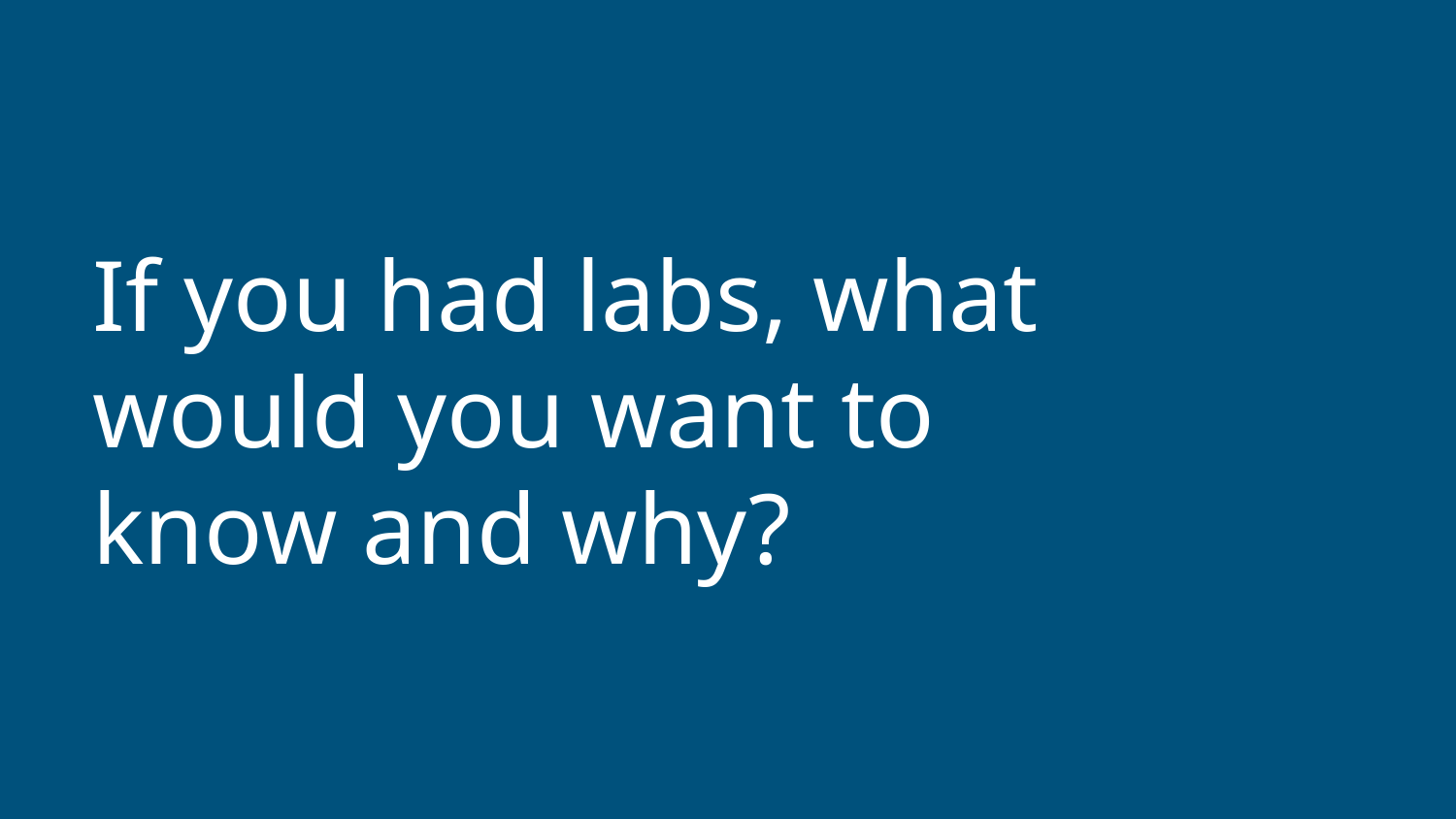

# If you had labs, what would you want to know and why?

## Slide 9
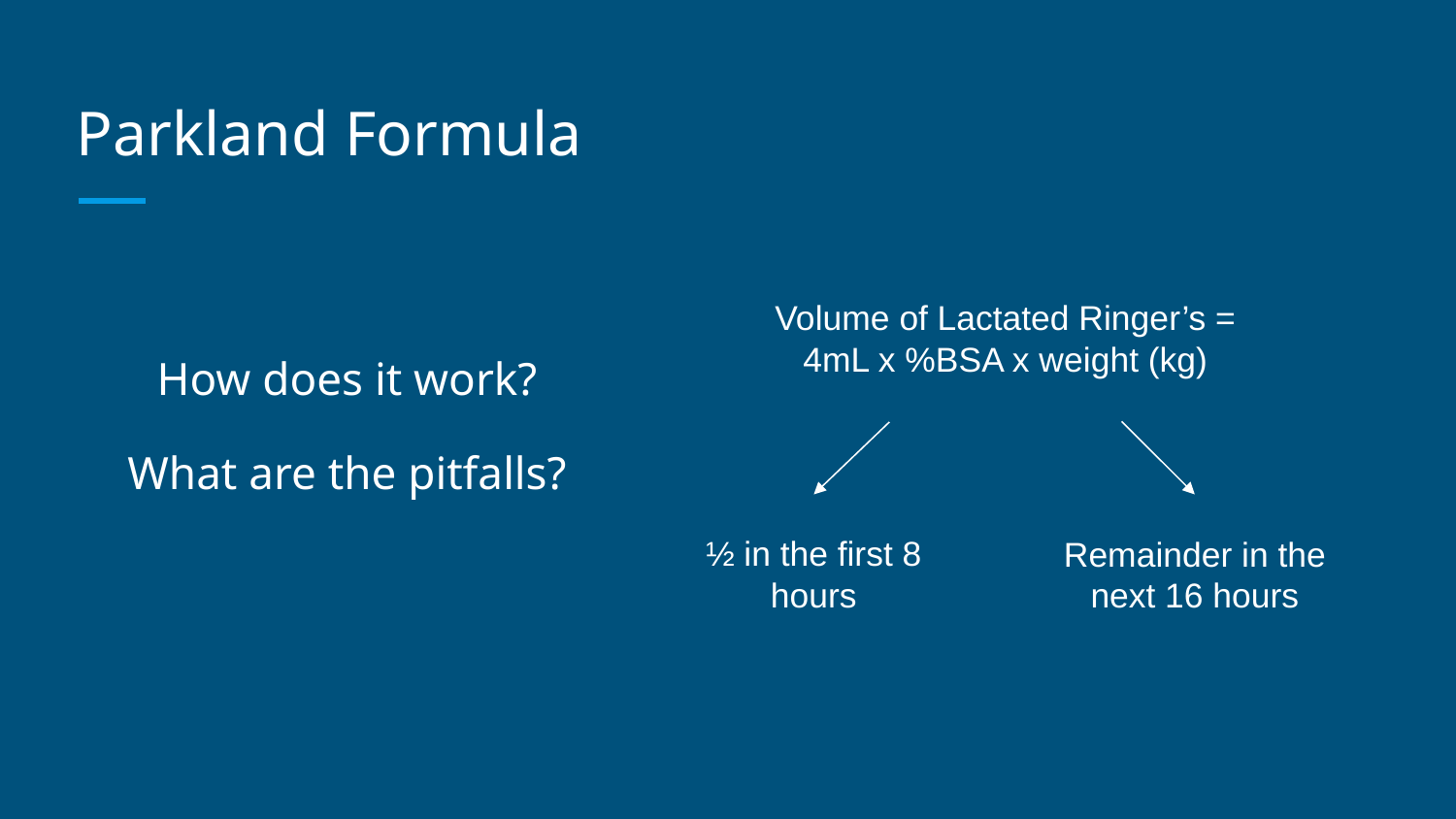

# Parkland Formula
Volume of Lactated Ringer’s =
4mL x %BSA x weight (kg)
½ in the first 8 hours
Remainder in the next 16 hours
How does it work?
What are the pitfalls?

## Slide 10
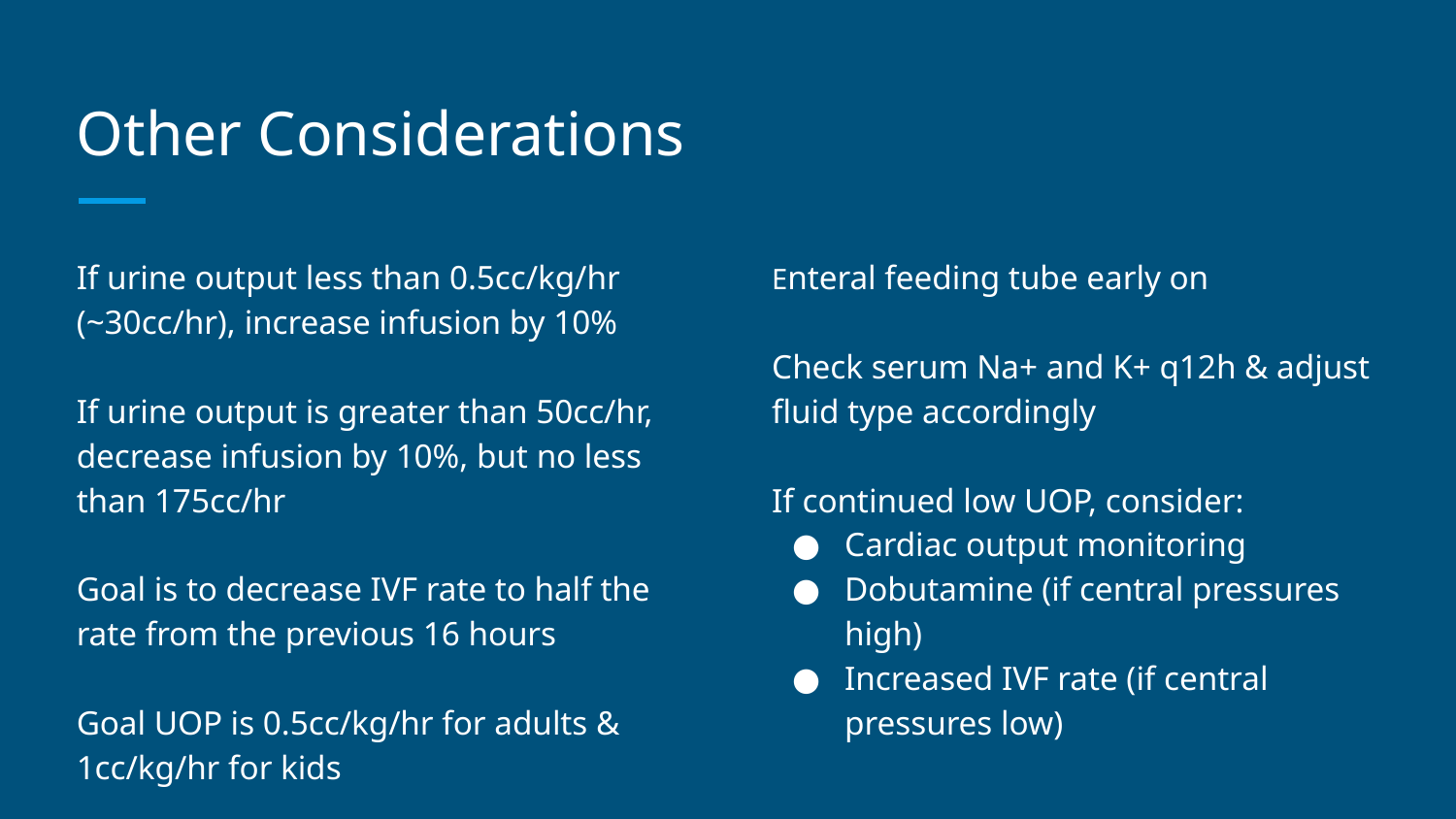

# Other Considerations
If urine output less than 0.5cc/kg/hr (~30cc/hr), increase infusion by 10%
If urine output is greater than 50cc/hr, decrease infusion by 10%, but no less than 175cc/hr
Goal is to decrease IVF rate to half the rate from the previous 16 hours
Goal UOP is 0.5cc/kg/hr for adults &
1cc/kg/hr for kids
Enteral feeding tube early on
Check serum Na+ and K+ q12h & adjust fluid type accordingly
If continued low UOP, consider:
Cardiac output monitoring
Dobutamine (if central pressures high)
Increased IVF rate (if central pressures low)

## Slide 11
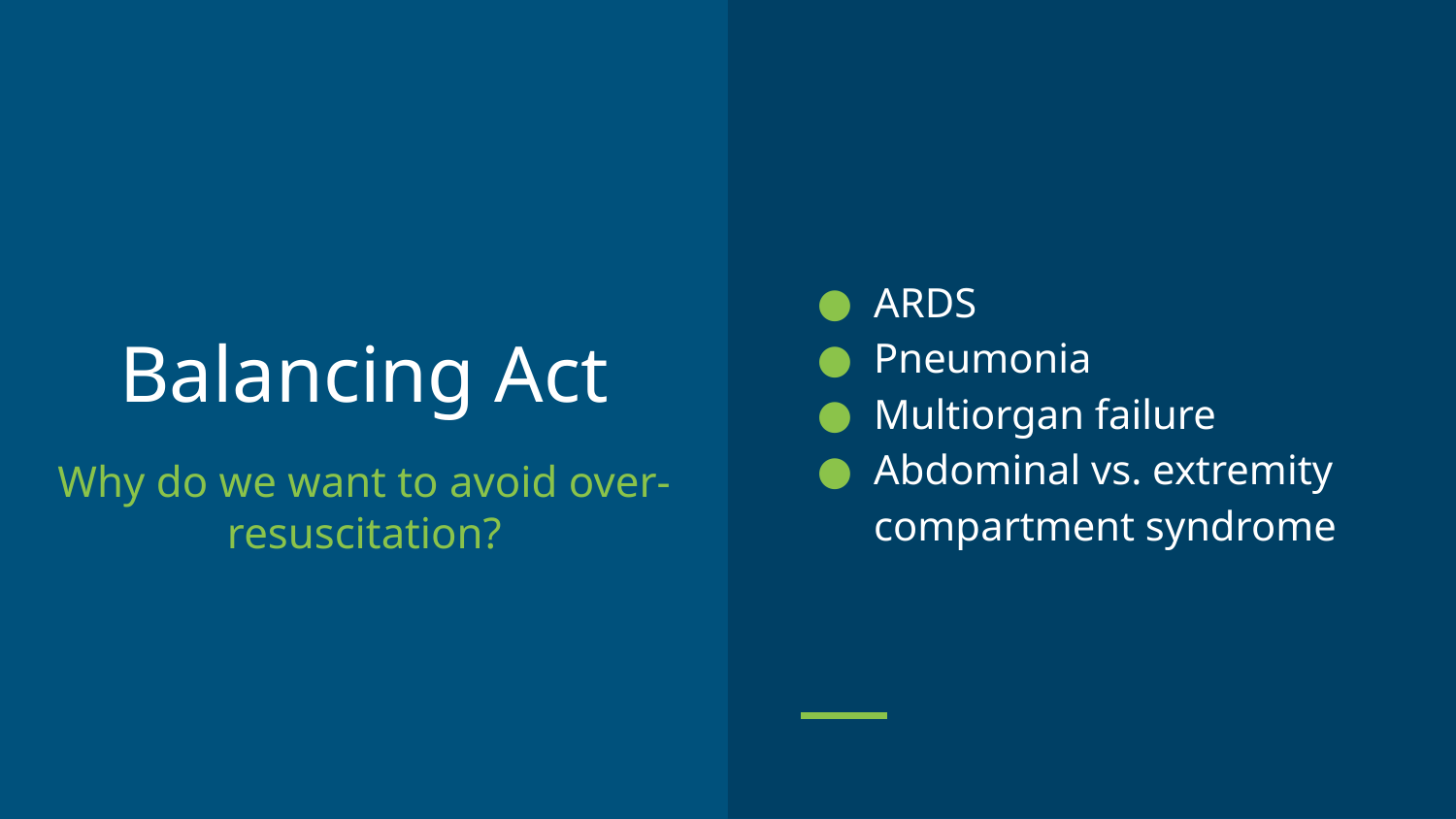

ARDS
Pneumonia
Multiorgan failure
Abdominal vs. extremity compartment syndrome
# Balancing Act
Why do we want to avoid over-resuscitation?

## Slide 12
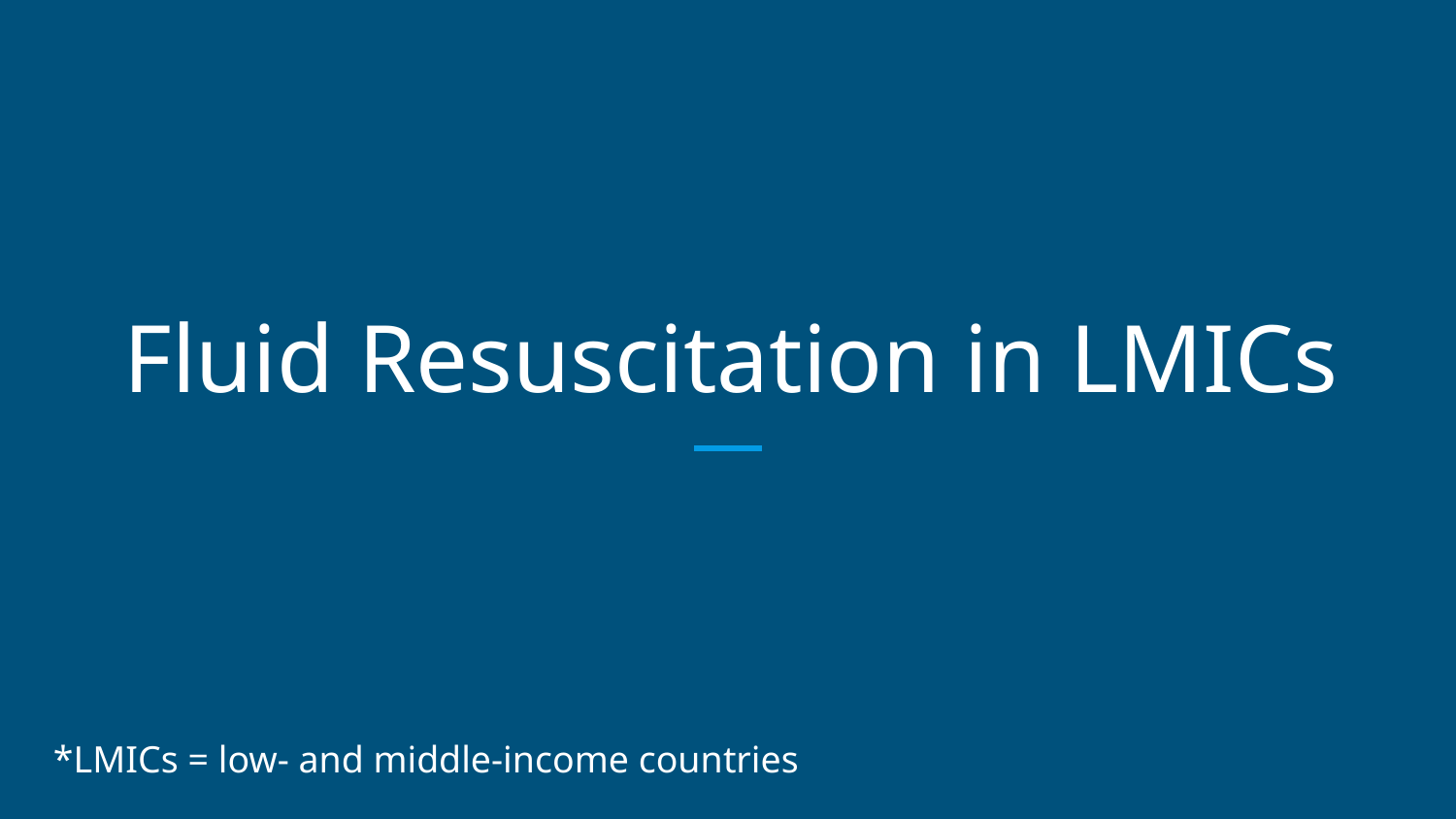

# Fluid Resuscitation in LMICs
*LMICs = low- and middle-income countries

## Slide 13
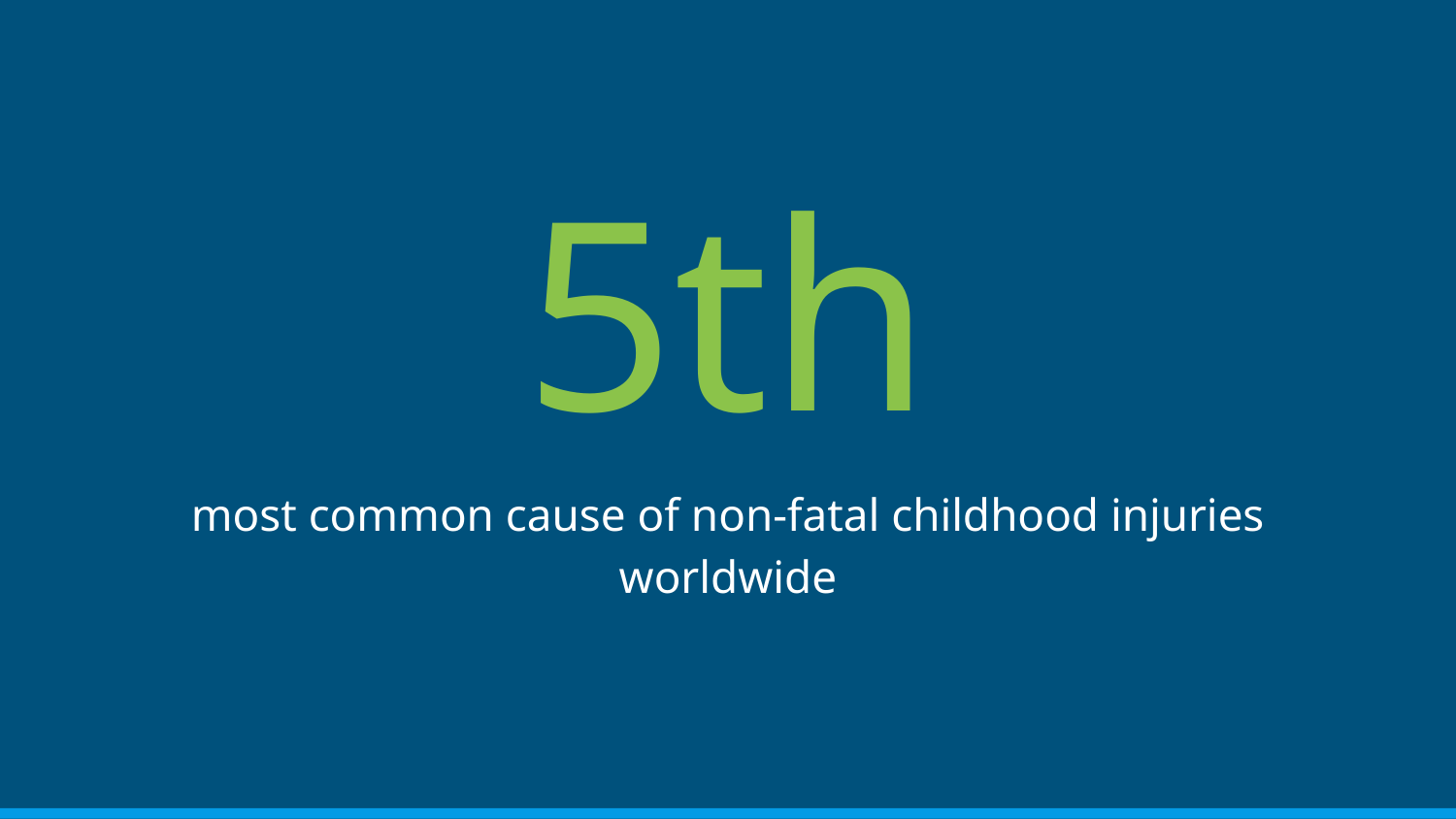

# 5th
most common cause of non-fatal childhood injuries worldwide

## Slide 14
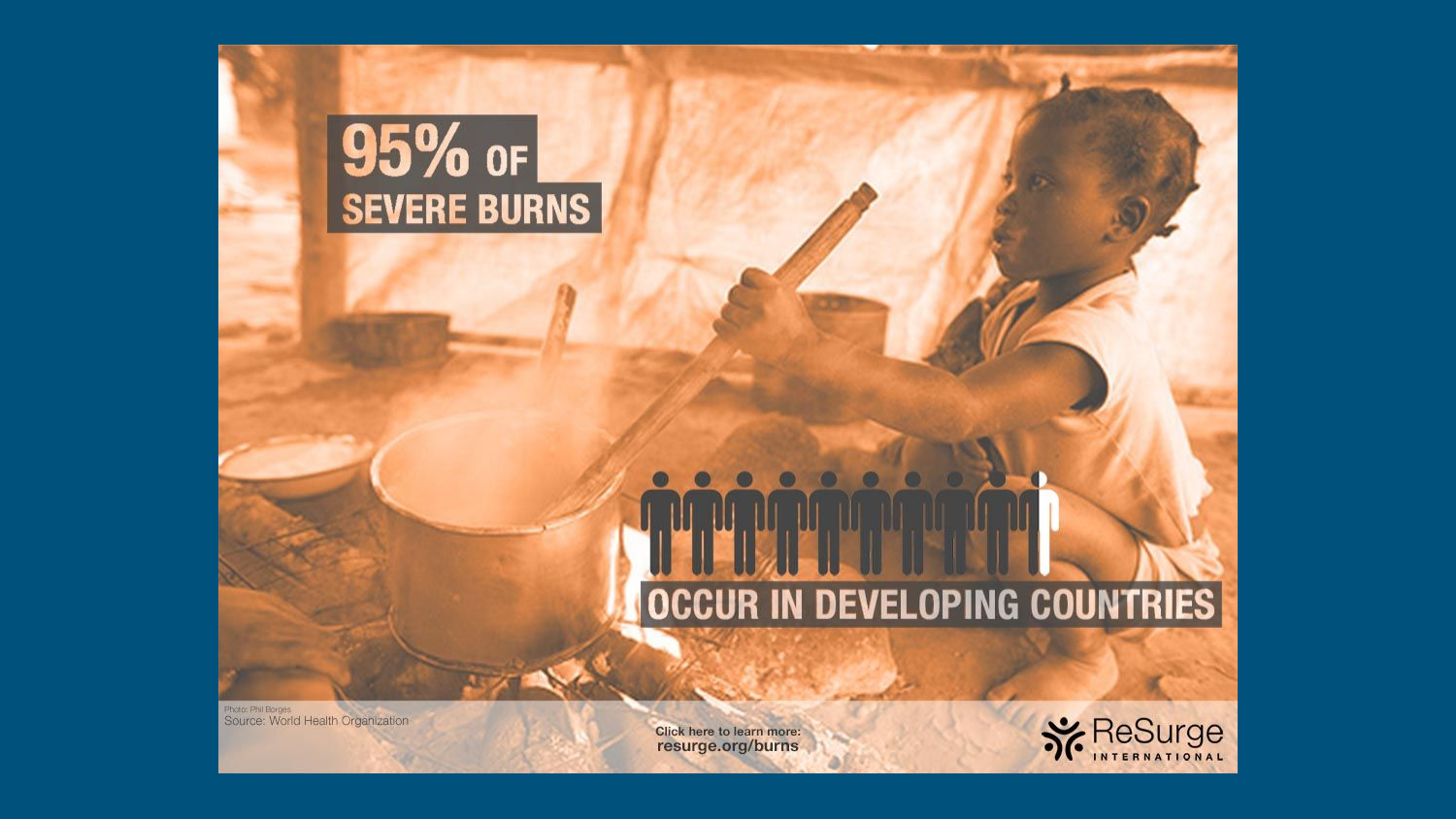

## Slide 15
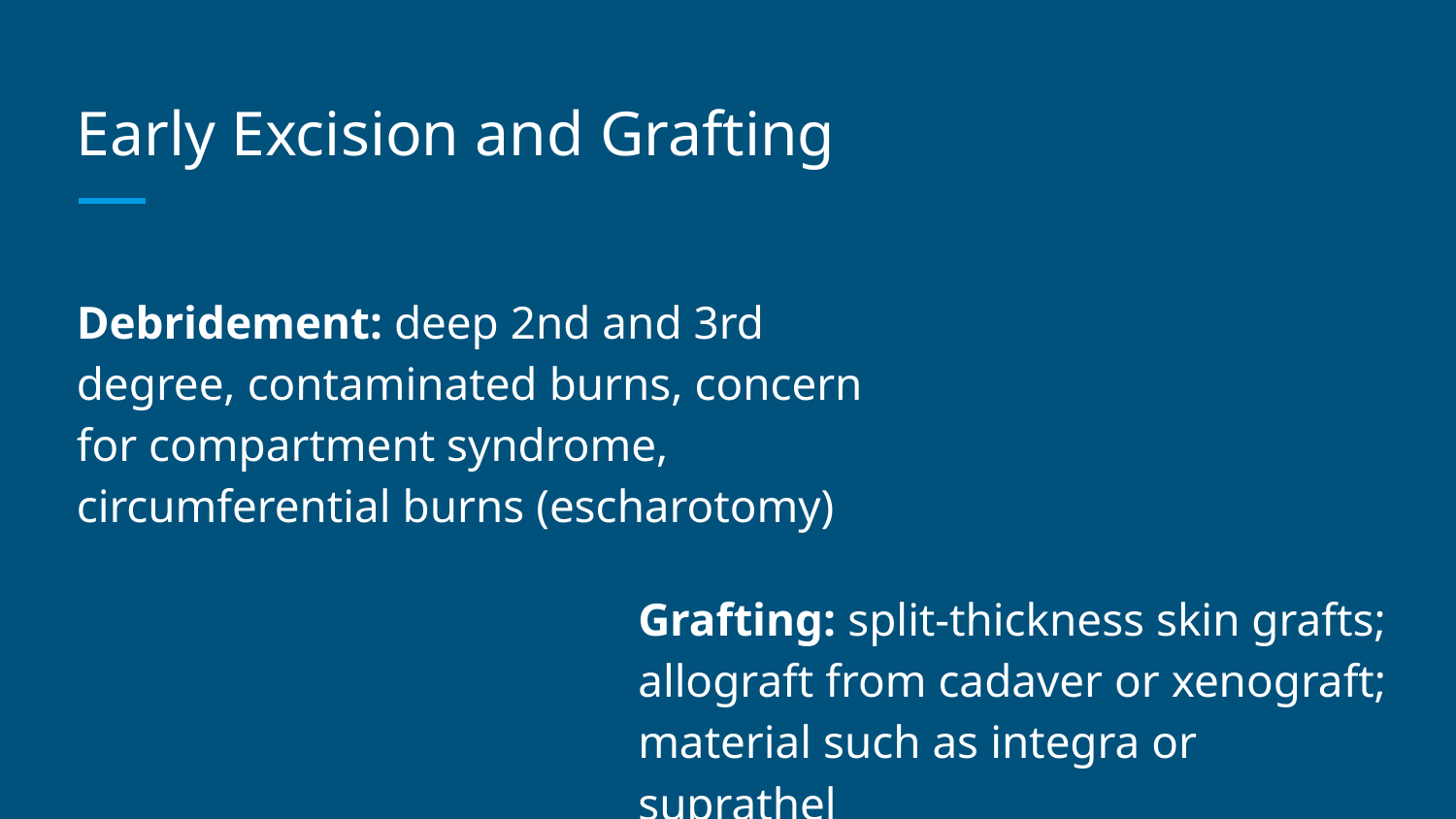

# Early Excision and Grafting
Debridement: deep 2nd and 3rd degree, contaminated burns, concern for compartment syndrome, circumferential burns (escharotomy)
Grafting: split-thickness skin grafts; allograft from cadaver or xenograft; material such as integra or suprathel

## Slide 16
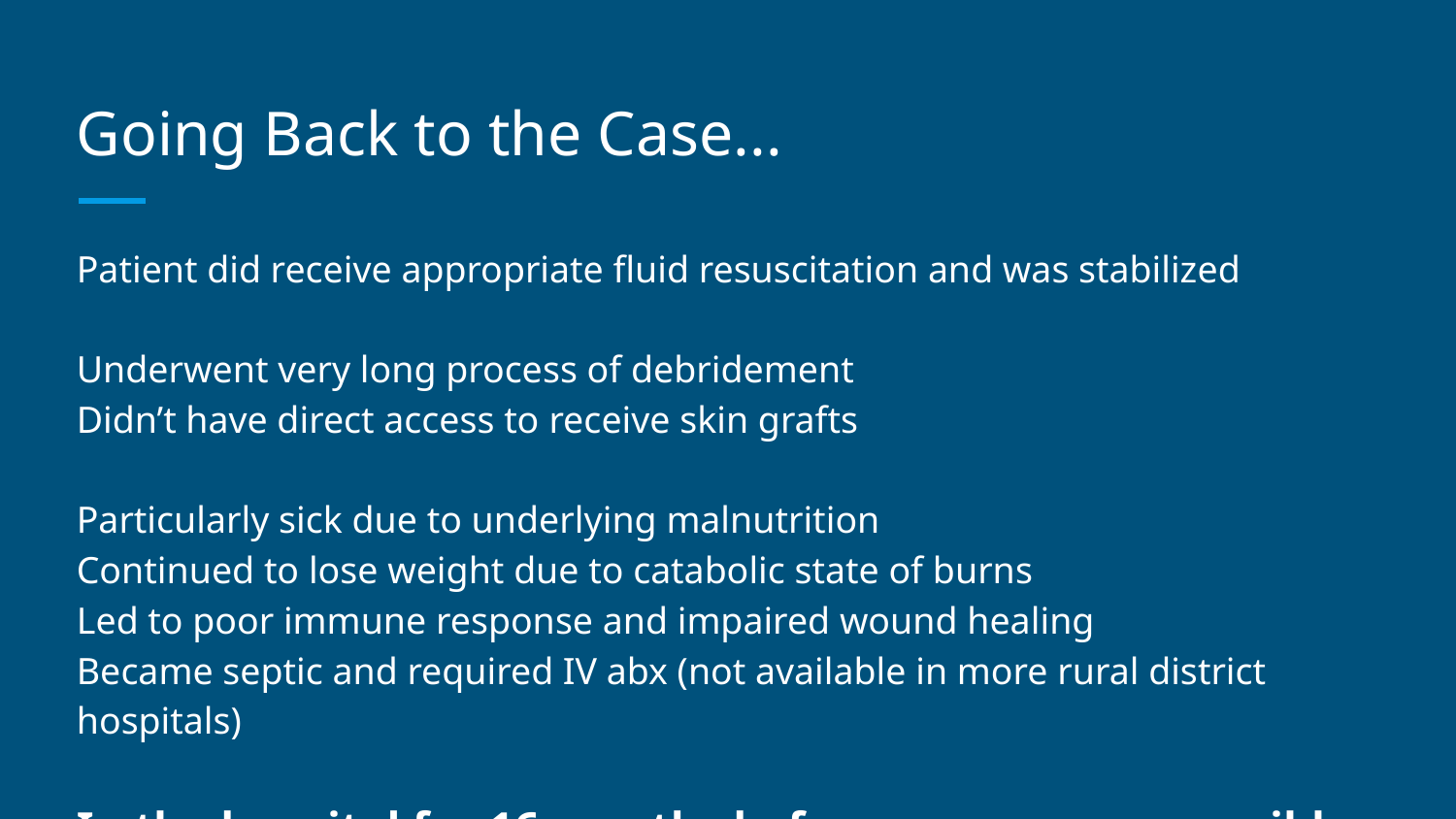

# Going Back to the Case...
Patient did receive appropriate fluid resuscitation and was stabilized
Underwent very long process of debridement
Didn’t have direct access to receive skin grafts
Particularly sick due to underlying malnutrition
Continued to lose weight due to catabolic state of burns
Led to poor immune response and impaired wound healing
Became septic and required IV abx (not available in more rural district hospitals)
In the hospital for 16 months before surgery was possible

## Slide 17
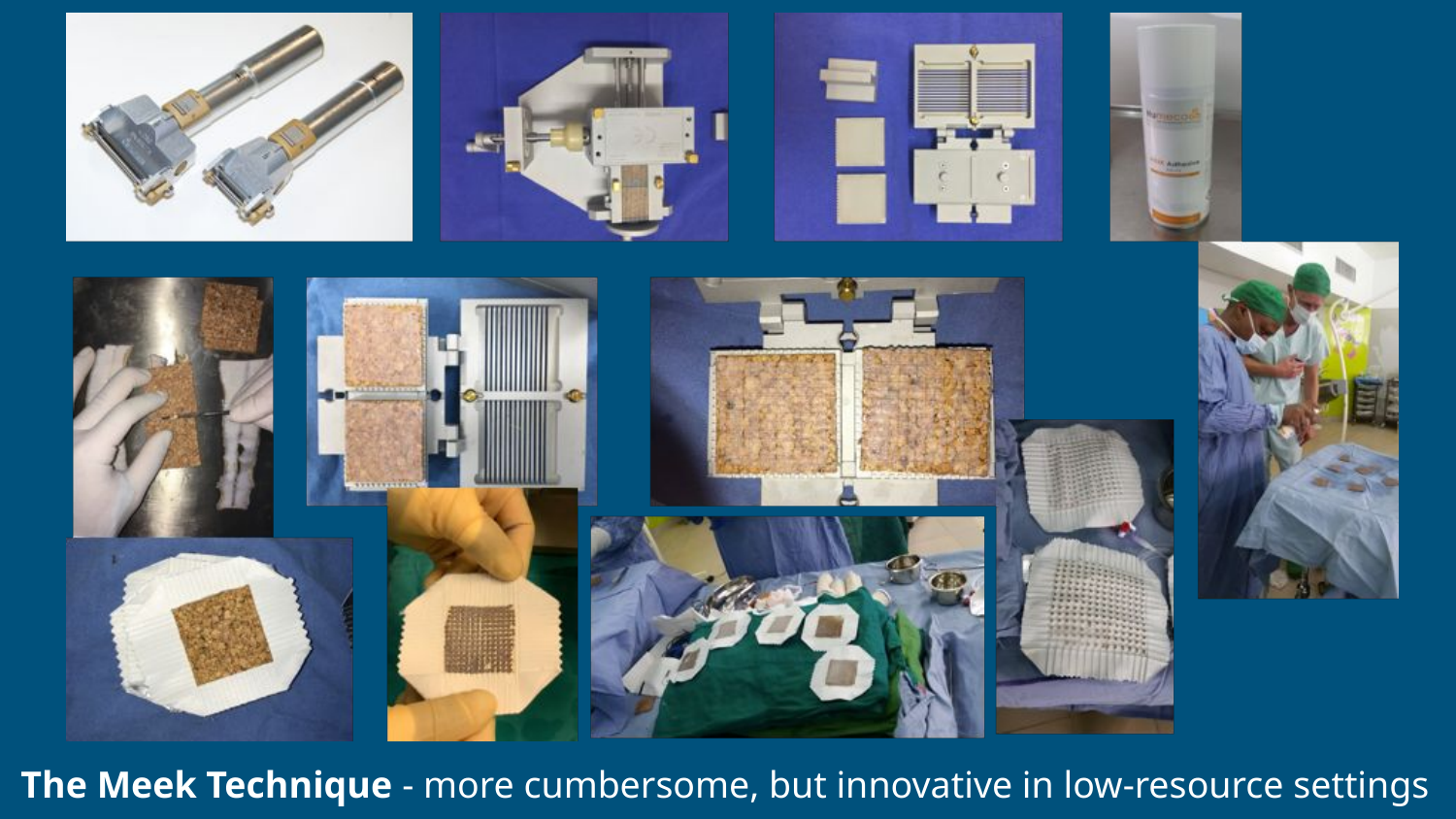

The Meek Technique - more cumbersome, but innovative in low-resource settings

## Slide 18
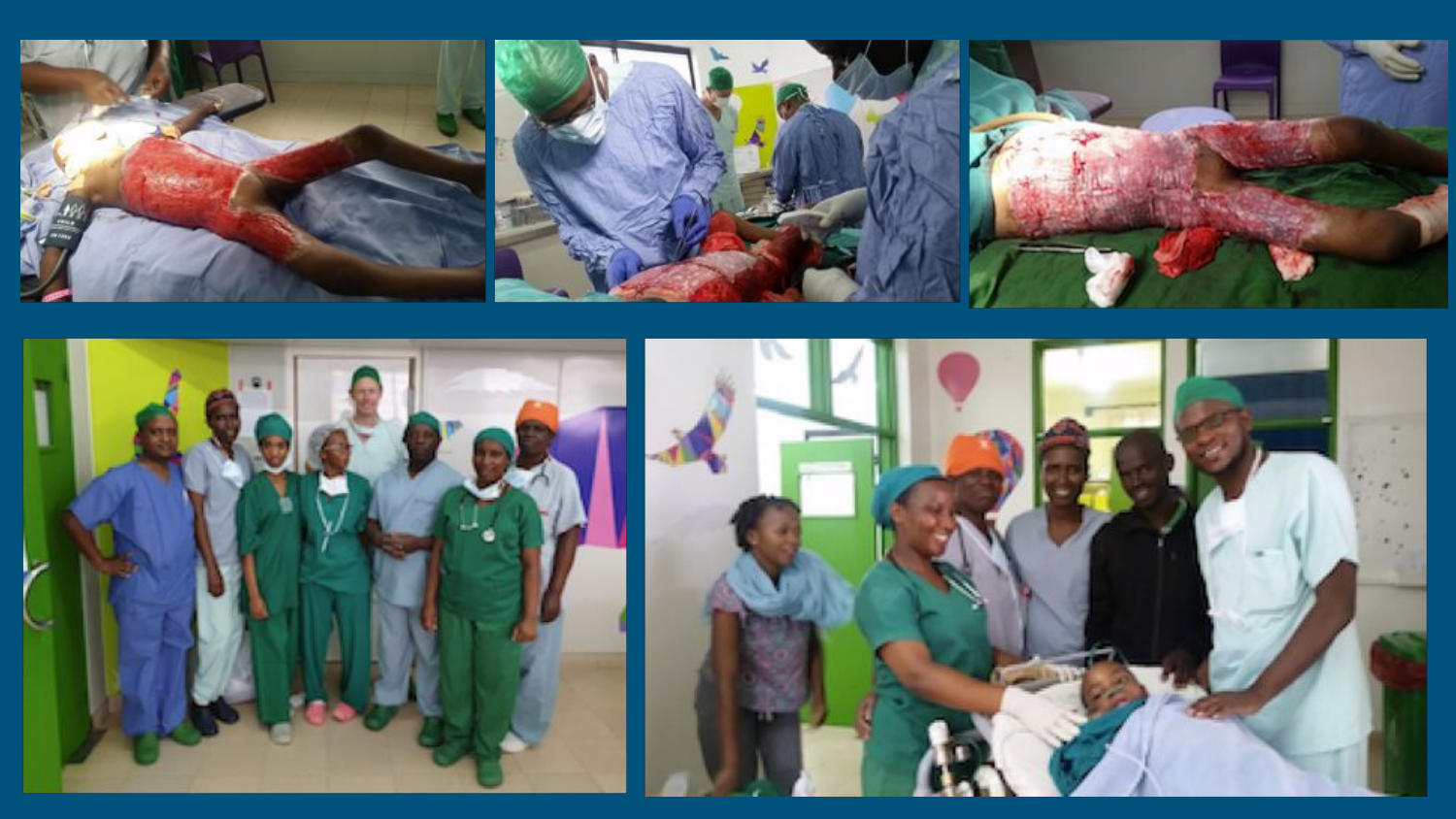

## Slide 19
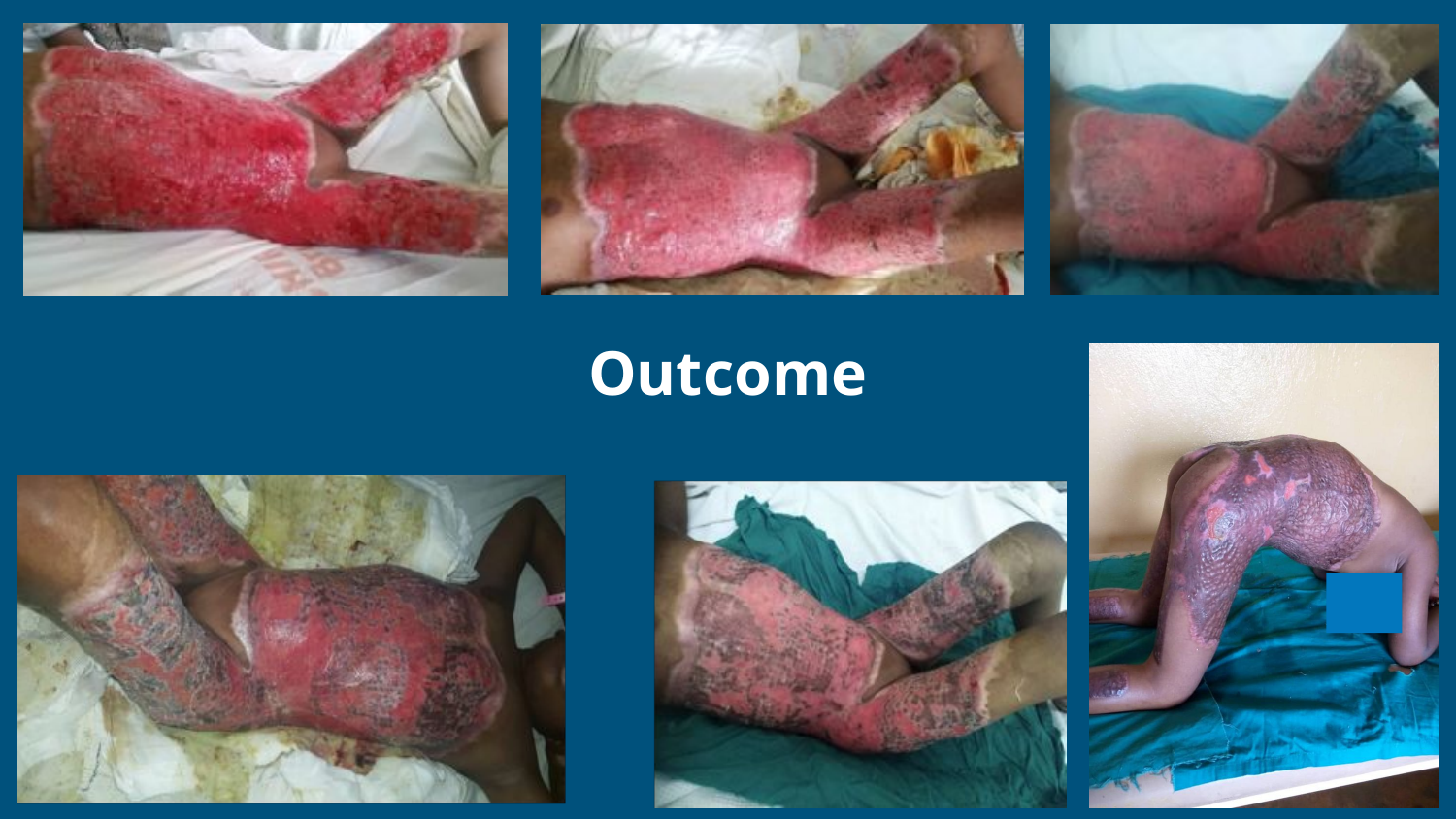

Outcome

## Slide 20
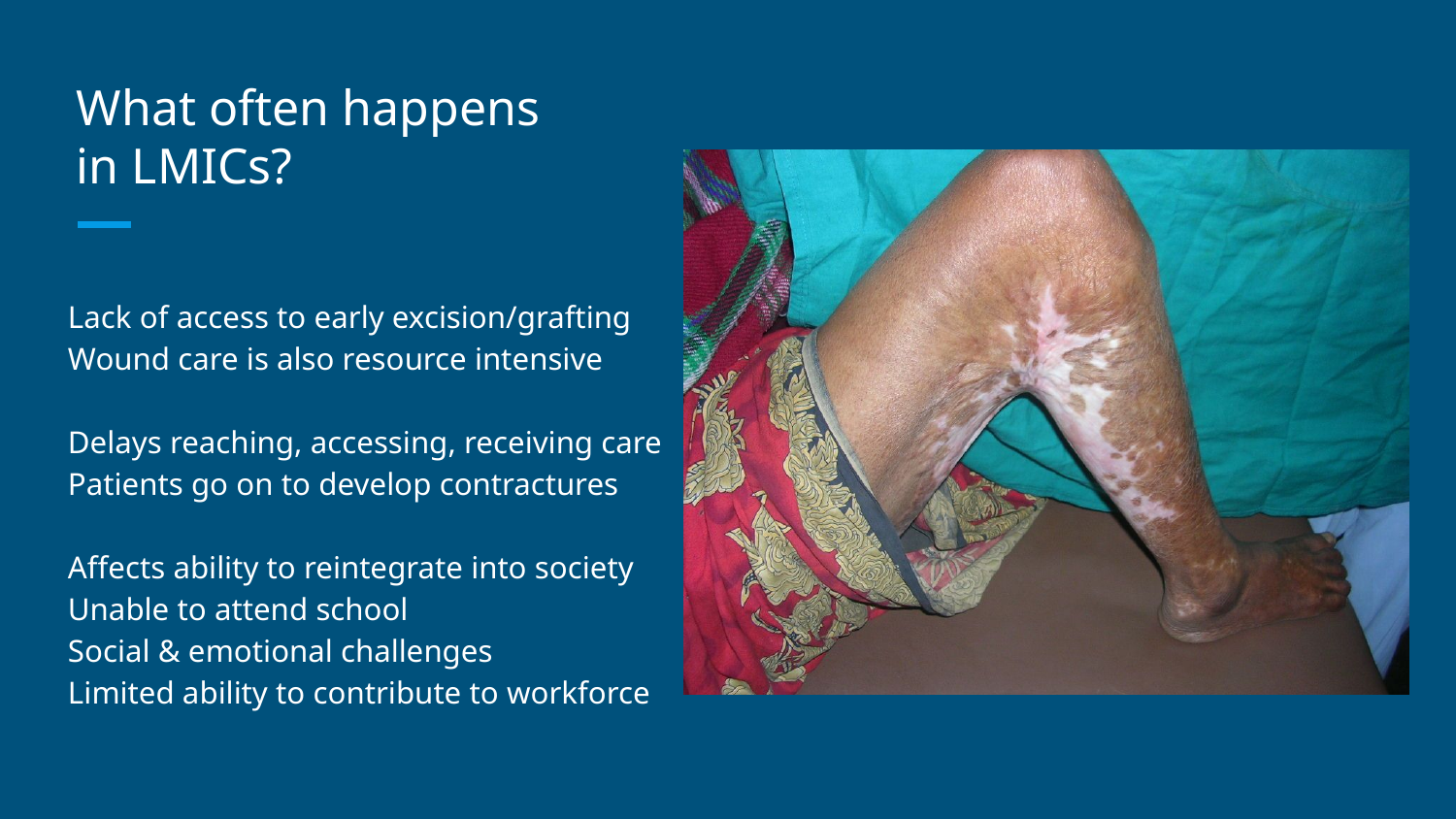

# What often happens in LMICs?
Lack of access to early excision/grafting
Wound care is also resource intensive
Delays reaching, accessing, receiving care
Patients go on to develop contractures
Affects ability to reintegrate into society
Unable to attend school
Social & emotional challenges
Limited ability to contribute to workforce

## Slide 21
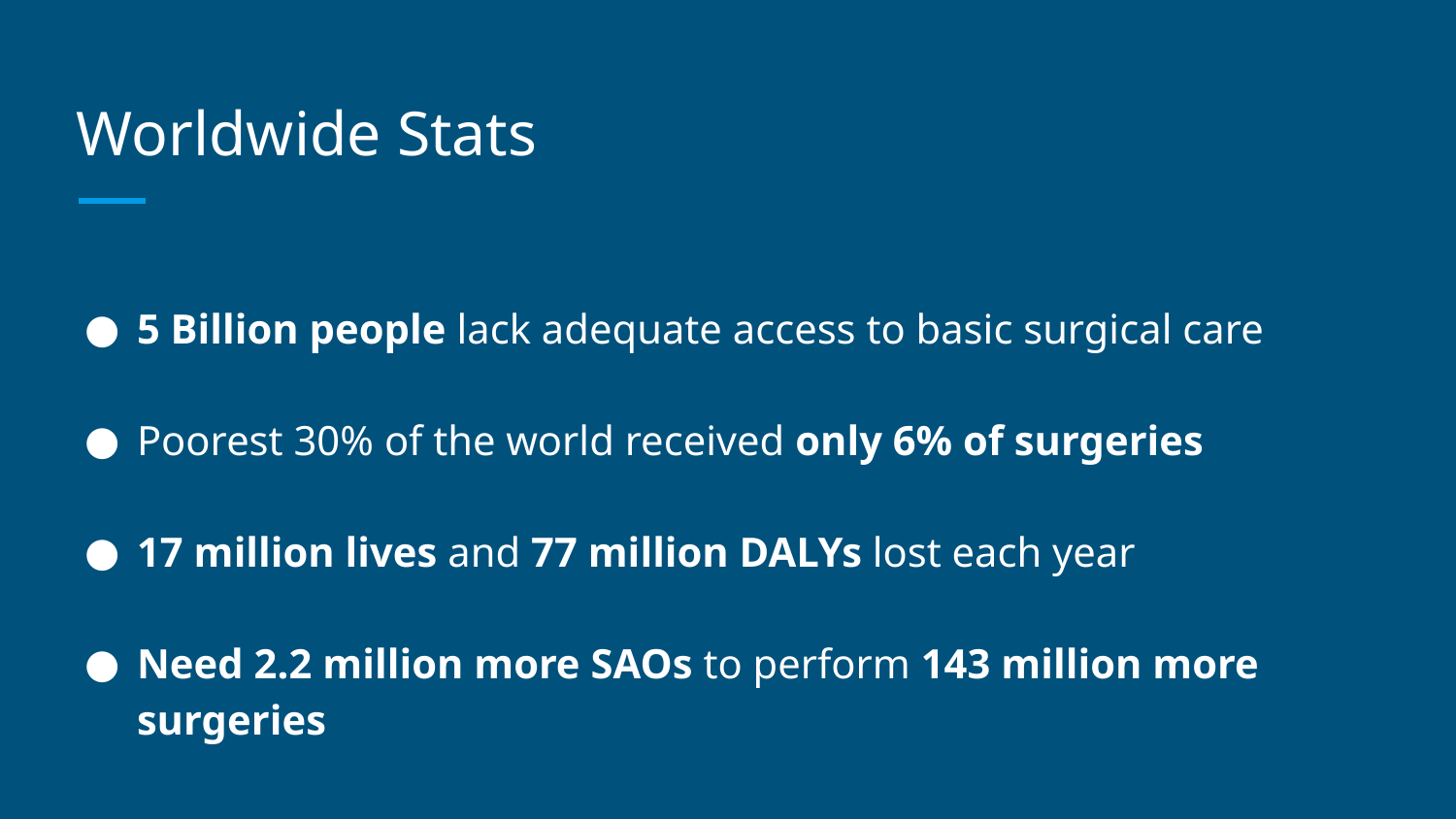

# Worldwide Stats
5 Billion people lack adequate access to basic surgical care
Poorest 30% of the world received only 6% of surgeries
17 million lives and 77 million DALYs lost each year
Need 2.2 million more SAOs to perform 143 million more surgeries

## Slide 22
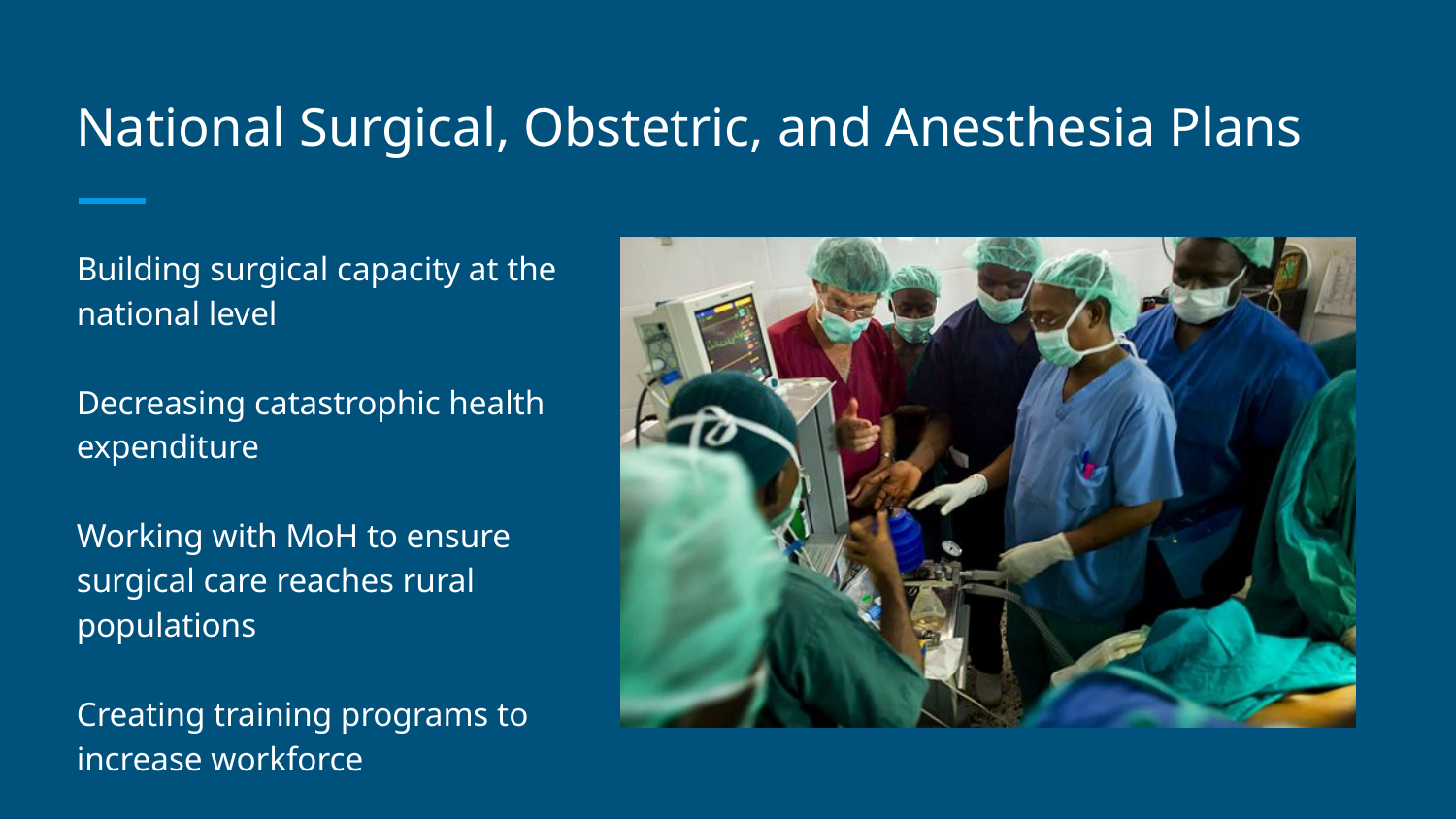

# National Surgical, Obstetric, and Anesthesia Plans
Building surgical capacity at the national level
Decreasing catastrophic health expenditure
Working with MoH to ensure surgical care reaches rural populations
Creating training programs to increase workforce

## Slide 23
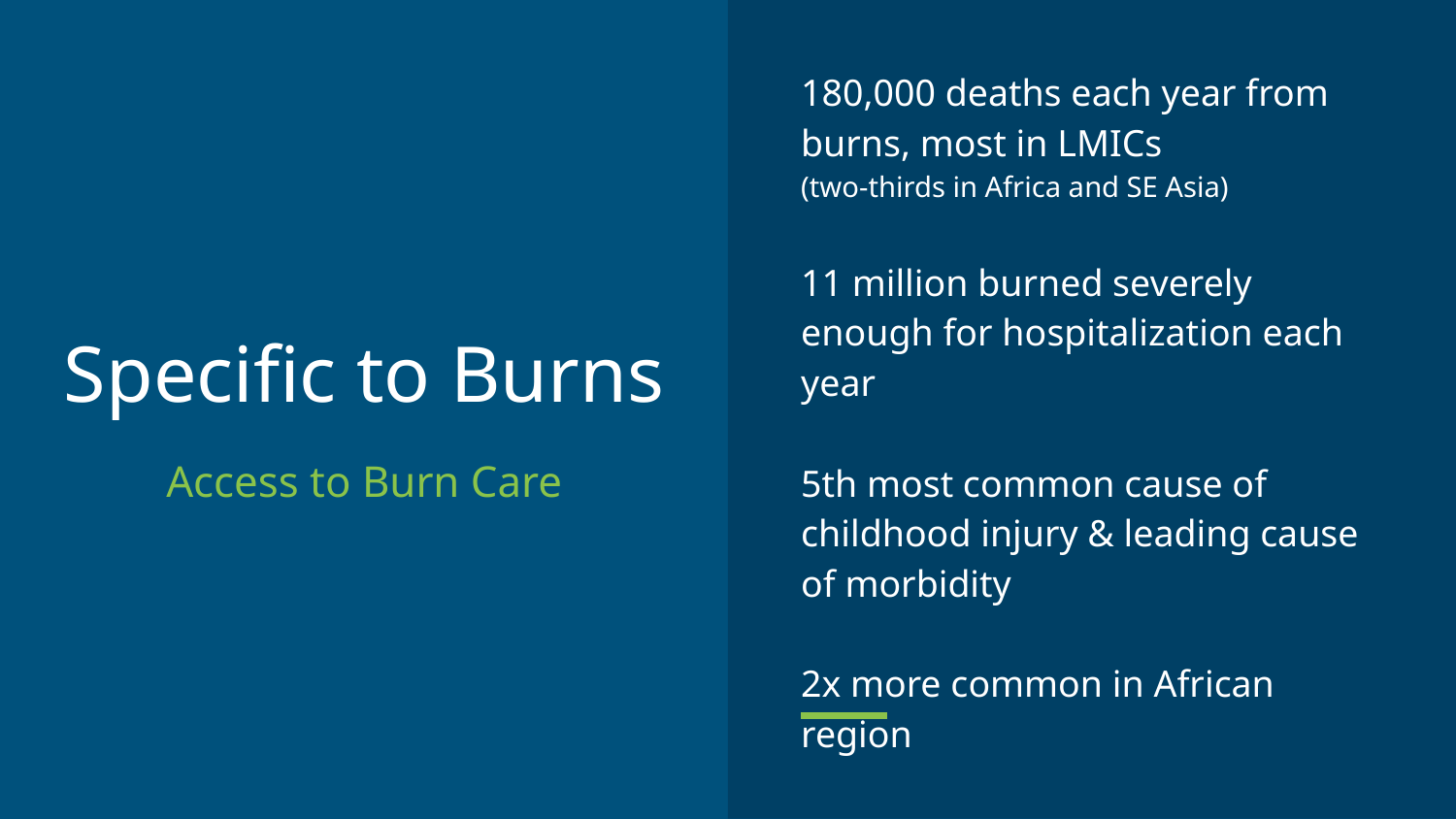

180,000 deaths each year from burns, most in LMICs
(two-thirds in Africa and SE Asia)
11 million burned severely enough for hospitalization each year
5th most common cause of childhood injury & leading cause of morbidity
2x more common in African region
# Specific to Burns
Access to Burn Care

## Slide 24
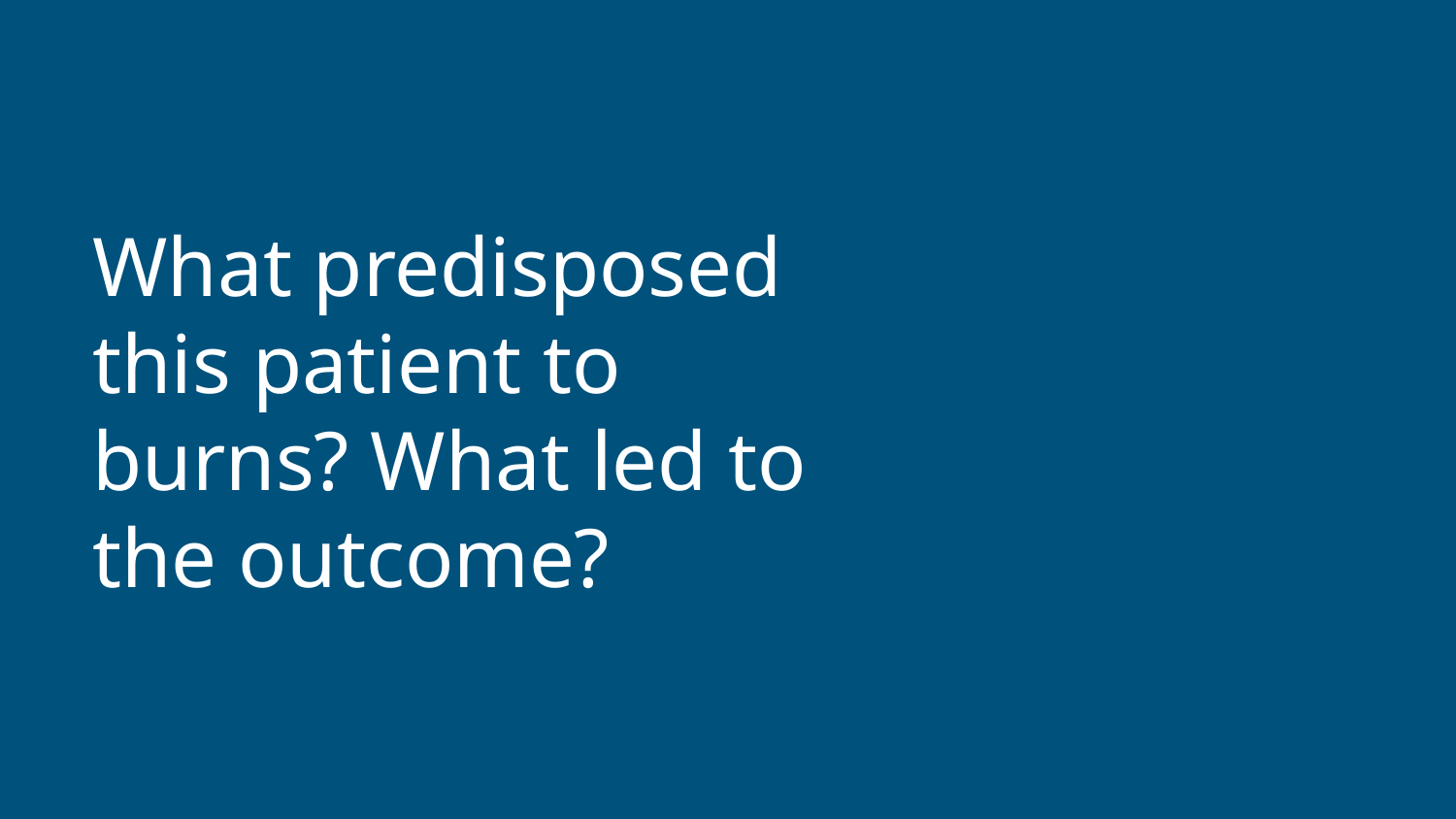

# What predisposed this patient to burns? What led to the outcome?

## Slide 25
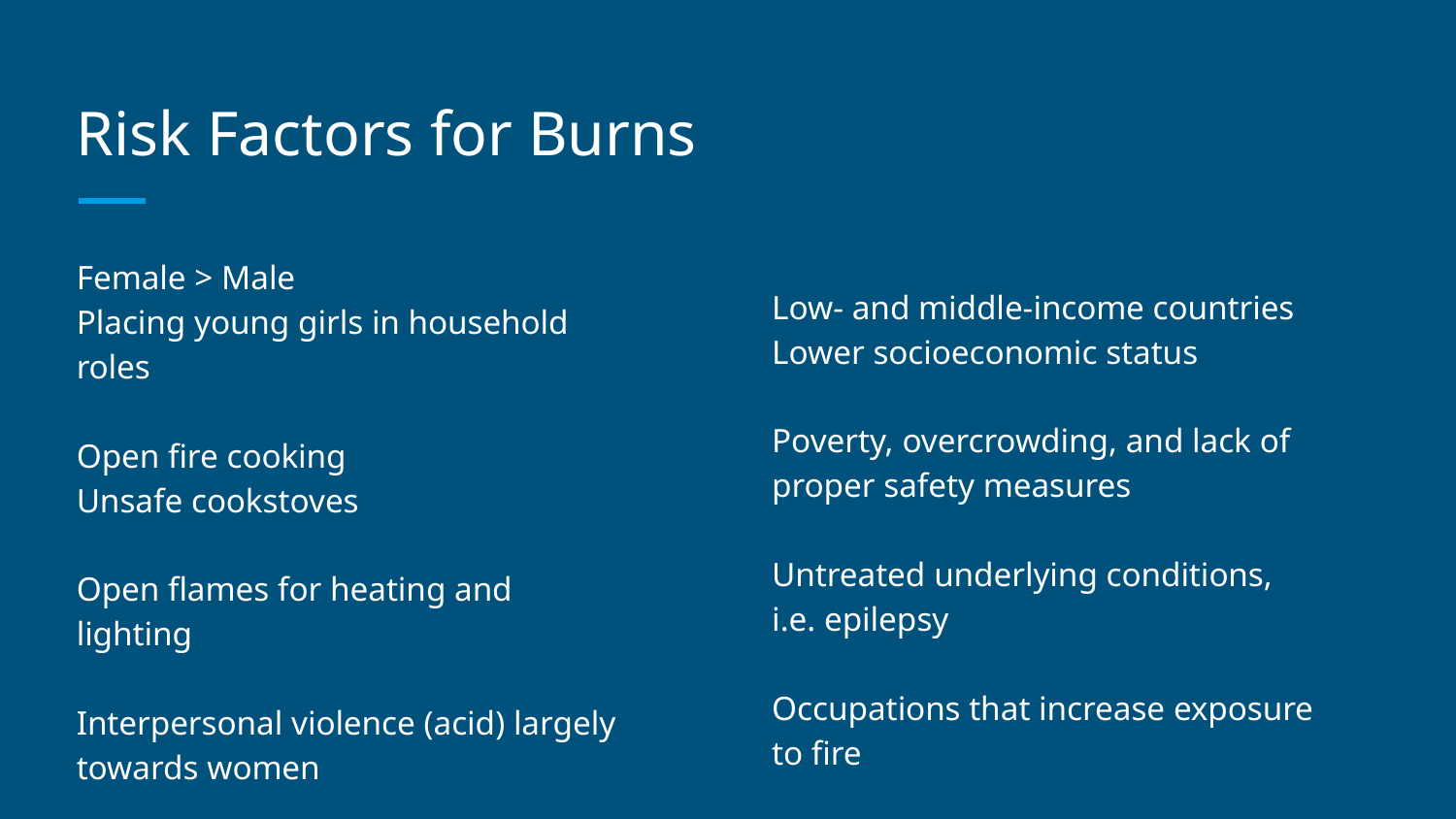

# Risk Factors for Burns
Female > Male
Placing young girls in household roles
Open fire cooking
Unsafe cookstoves
Open flames for heating and lighting
Interpersonal violence (acid) largely towards women
Alcohol use and smoking
Low- and middle-income countries
Lower socioeconomic status
Poverty, overcrowding, and lack of proper safety measures
Untreated underlying conditions, i.e. epilepsy
Occupations that increase exposure to fire

## Slide 26
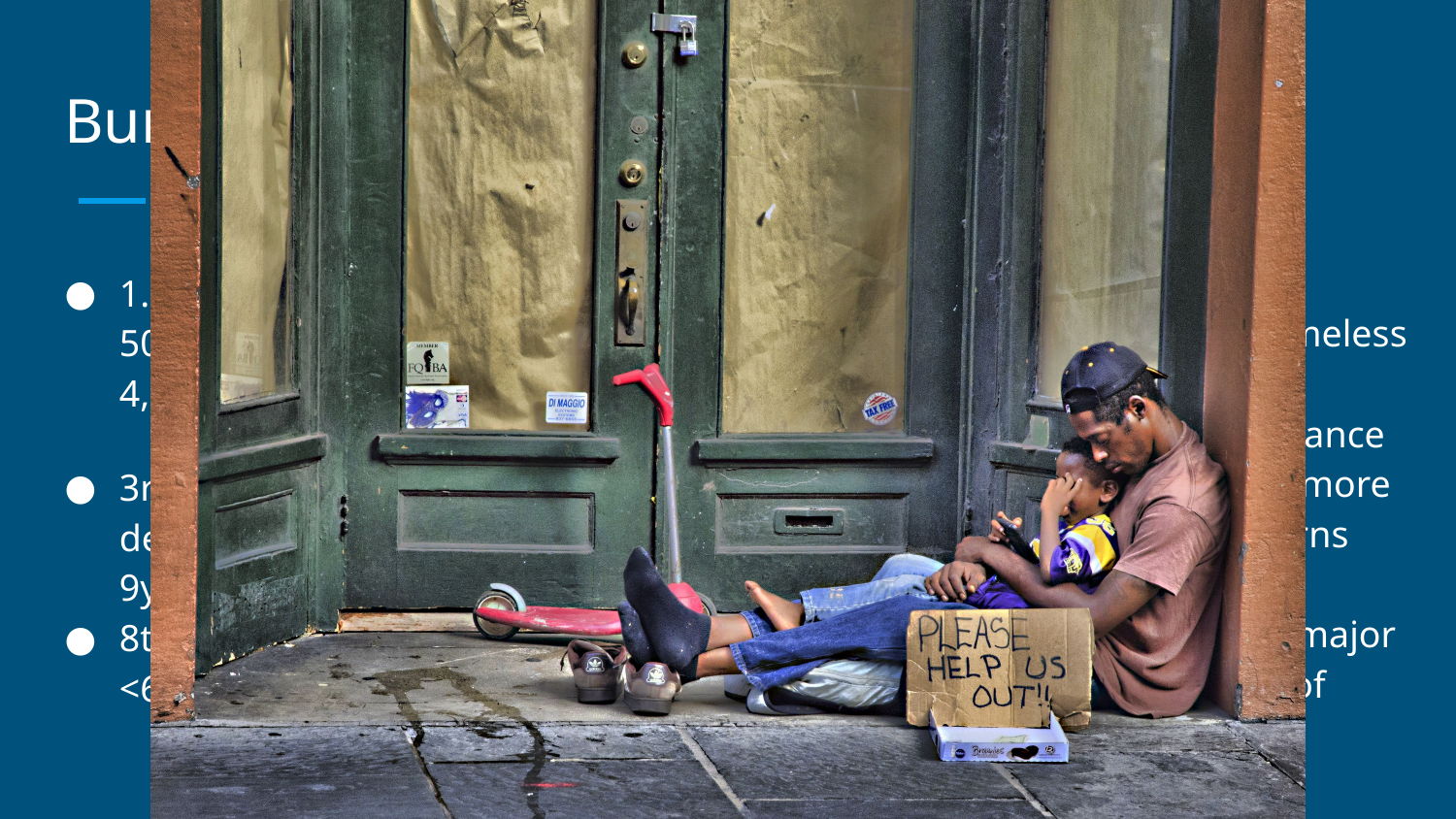

# Burns in the U.S. & Vulnerable Populations
1.1 million burns annually
50,000 require hospitalization
4,500 people die
3rd leading cause of accidental death amongst children age 5-9yrs
8th leading cause of death for <65yrs
Over 500,000 people are homeless in the U.S.
Face challenges of substance use, mental health, and more that increase risk for burns
Low-income housing is also major risk factor (house fires, lack of smoke detectors)

## Slide 27
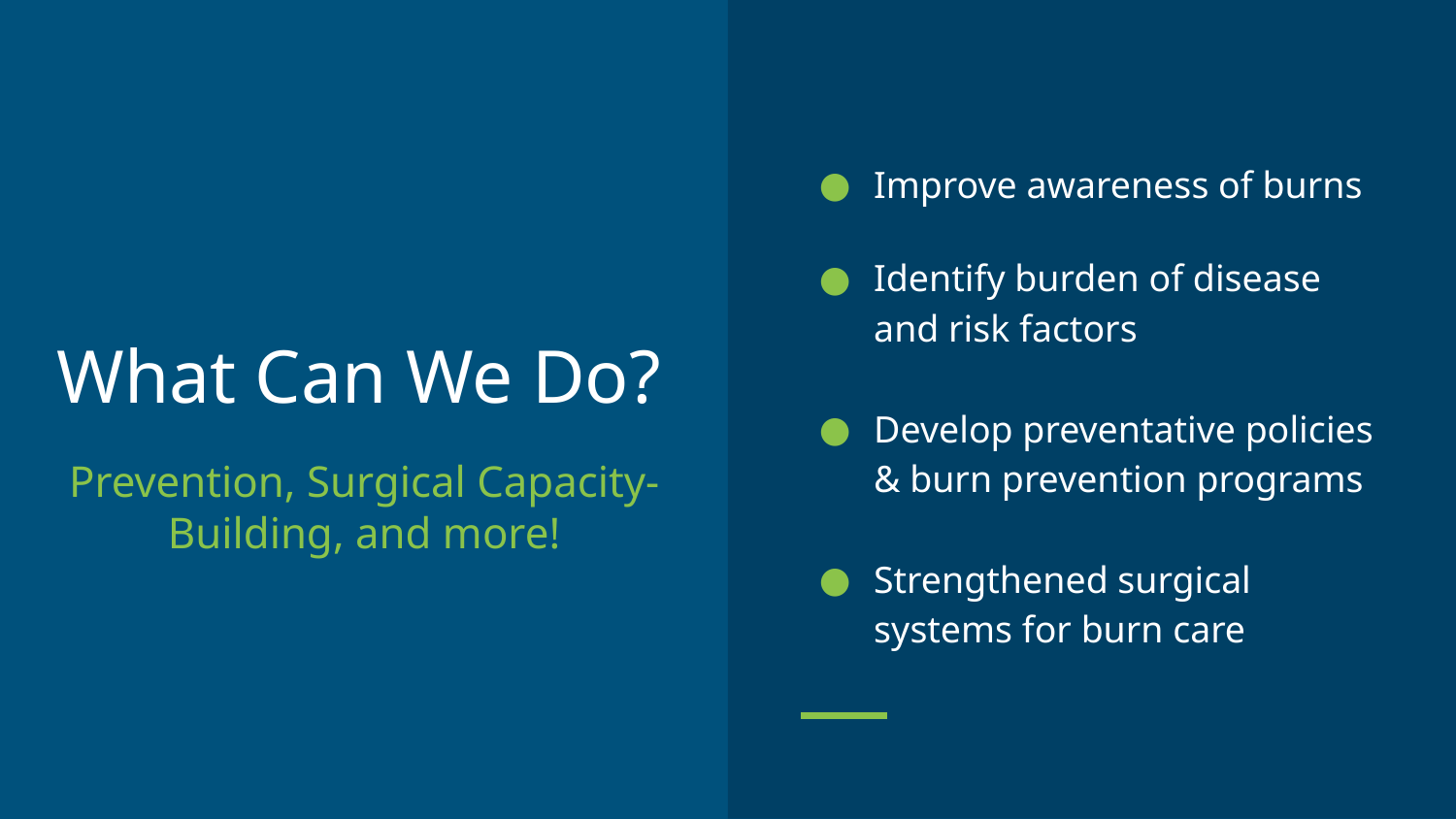

Improve awareness of burns
Identify burden of disease and risk factors
Develop preventative policies & burn prevention programs
Strengthened surgical systems for burn care
# What Can We Do?
Prevention, Surgical Capacity-Building, and more!

## Slide 28
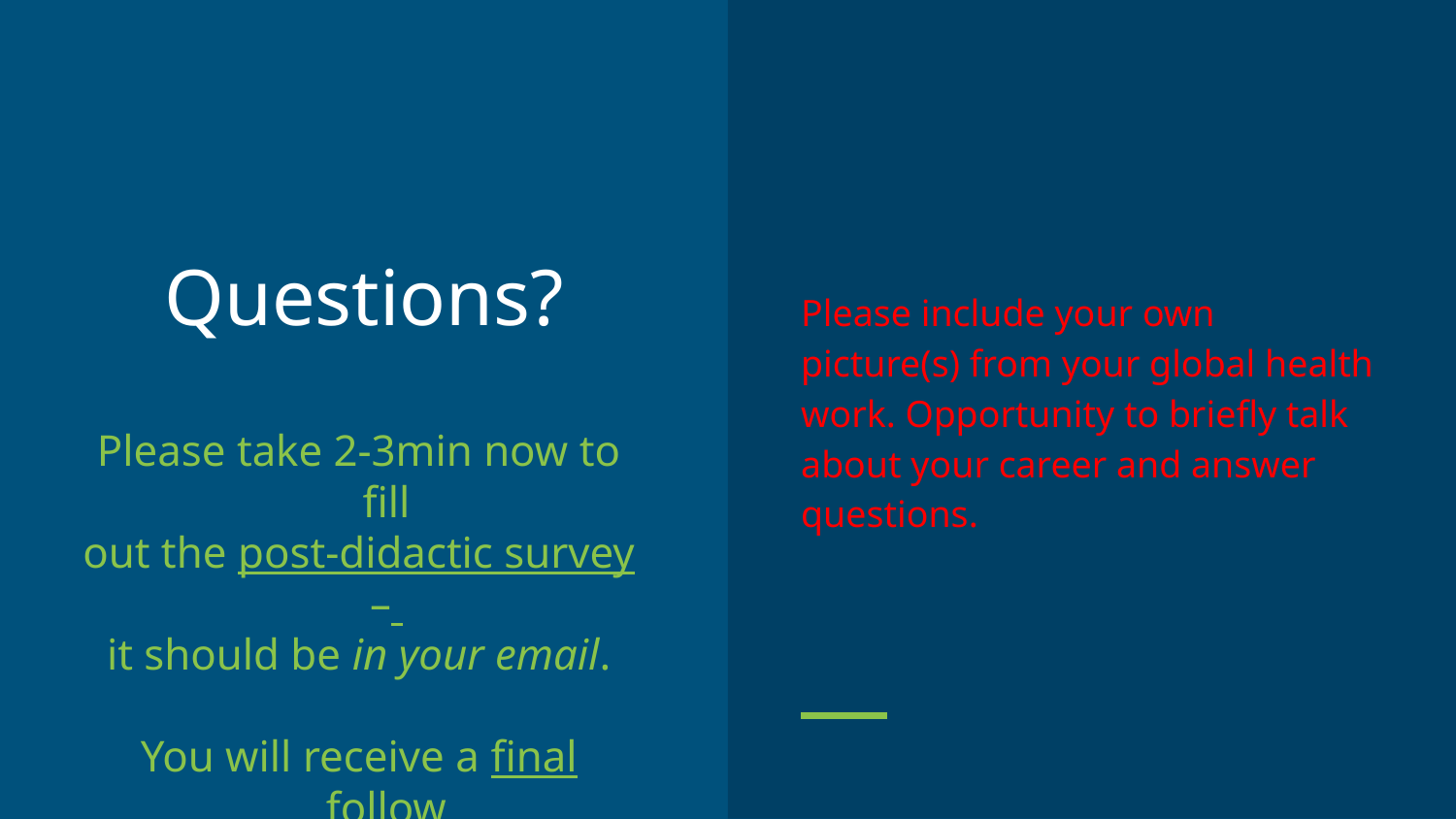

# Questions?
Please include your own picture(s) from your global health work. Opportunity to briefly talk about your career and answer questions.
Please take 2-3min now to fill
out the post-didactic survey –
it should be in your email.
You will receive a final follow
up survey in 2 weeks.
