# Supplementary material for: A Longitudinal Case-Based Global Health Curriculum for the Medical Student Clerkship Year
Source: MedEdPORTAL. 2020 Dec 8;16:11038. doi: 10.15766/mep_2374-8265.11038 (PMC7732136; doi:10.15766/mep_2374-8265.11038)
Supplement: Supplementary file 1 — Clerkship Director Proposal.pptxProject Description.docxPediatrics GH Didactic.pptxSurgery GH Didactic.pptxMedicine GH Didactic.pptxFacilitator Notes.docxPredidactic Survey.docxPostdidactic Survey.docxFollow-up Survey.docx [file mep_2374-8265.11038-s001.zip › E. Medicine GH Didactic.pptx]

## Slide 1
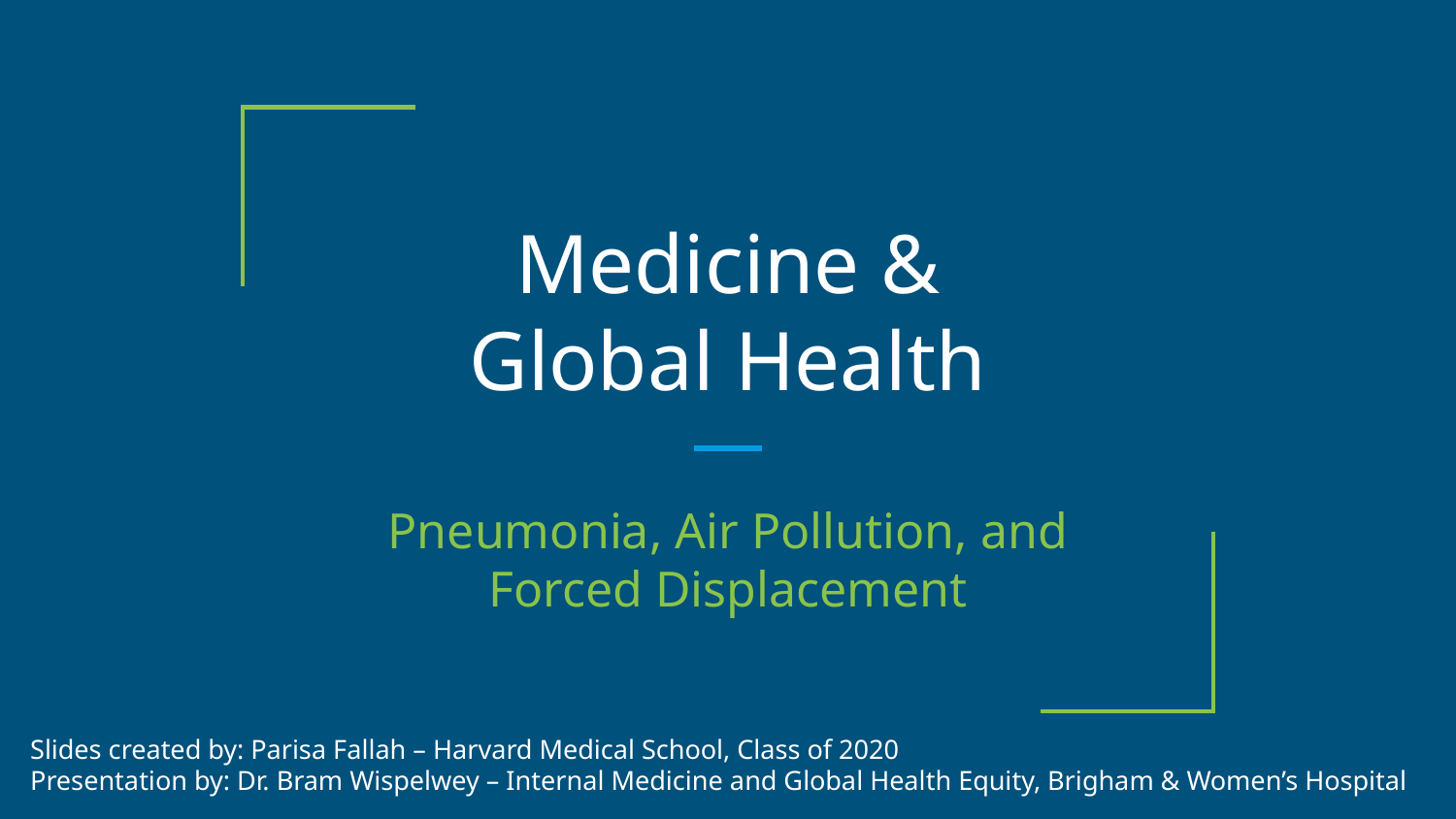

# Medicine &
Global Health
Pneumonia, Air Pollution, and
Forced Displacement
Slides created by: Parisa Fallah – Harvard Medical School, Class of 2020
Presentation by: Dr. Bram Wispelwey – Internal Medicine and Global Health Equity, Brigham & Women’s Hospital

## Slide 2
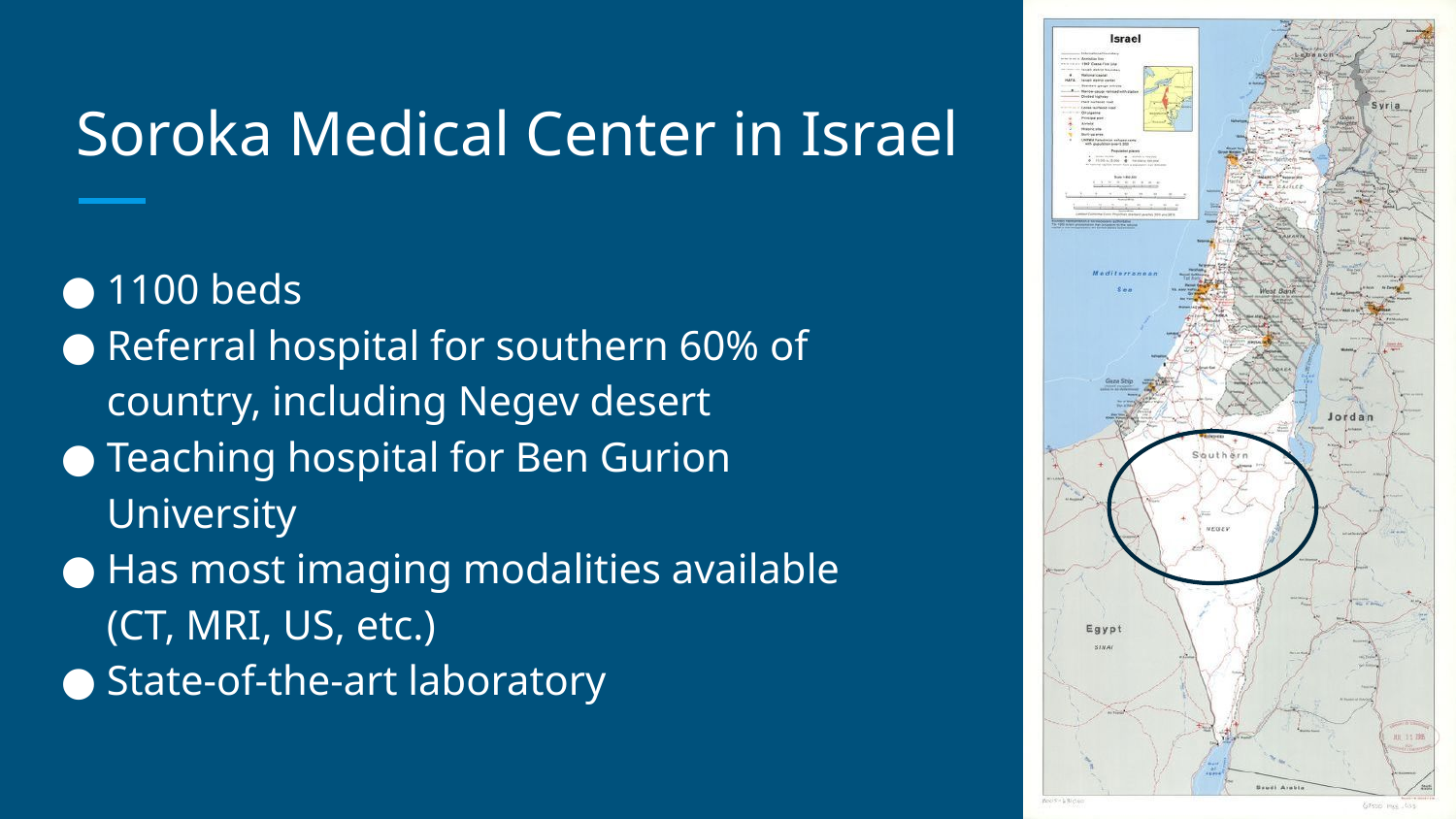

# Soroka Medical Center in Israel
1100 beds
Referral hospital for southern 60% of country, including Negev desert
Teaching hospital for Ben Gurion University
Has most imaging modalities available (CT, MRI, US, etc.)
State-of-the-art laboratory

## Slide 3
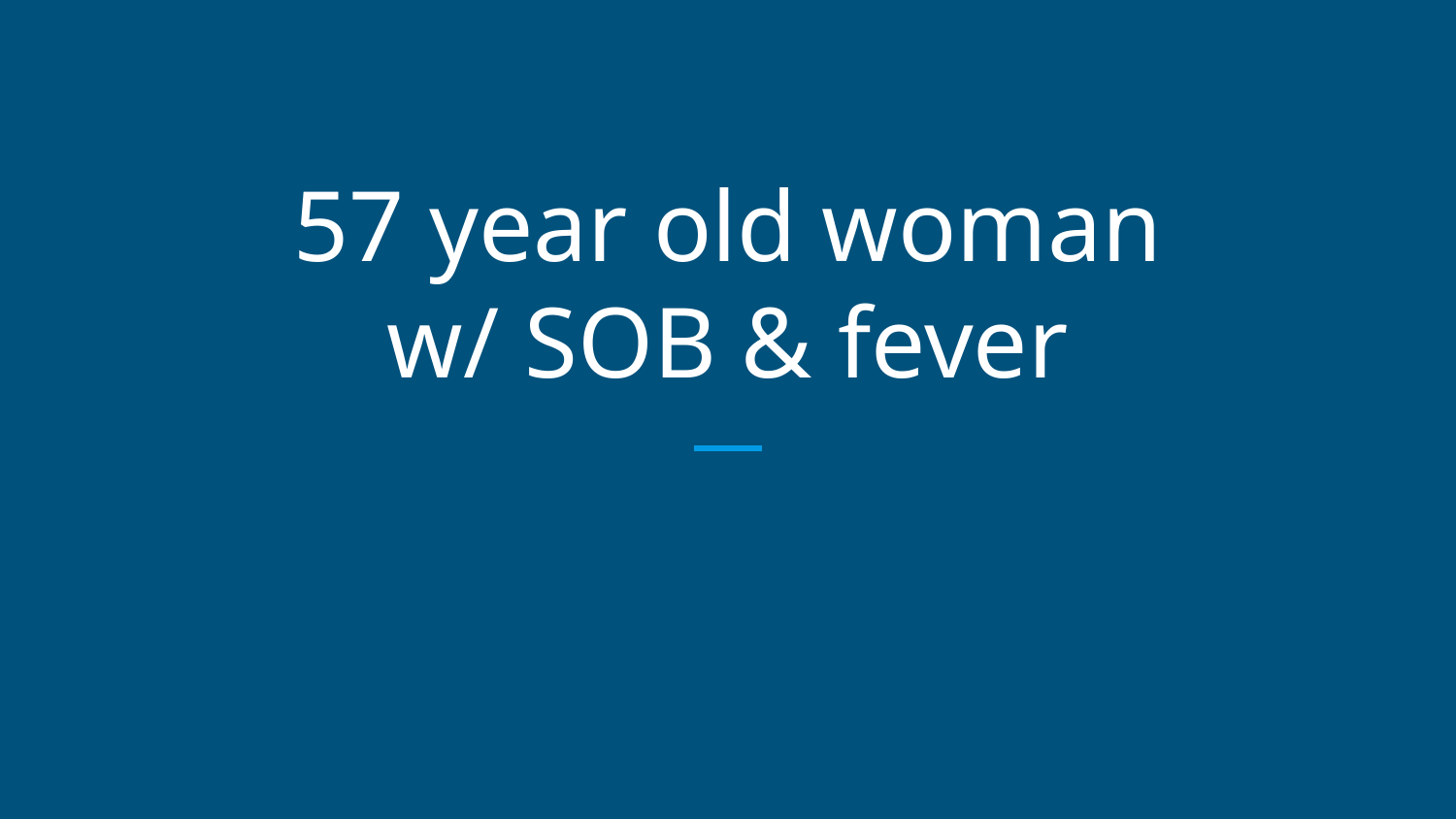

# 57 year old woman w/ SOB & fever

## Slide 4
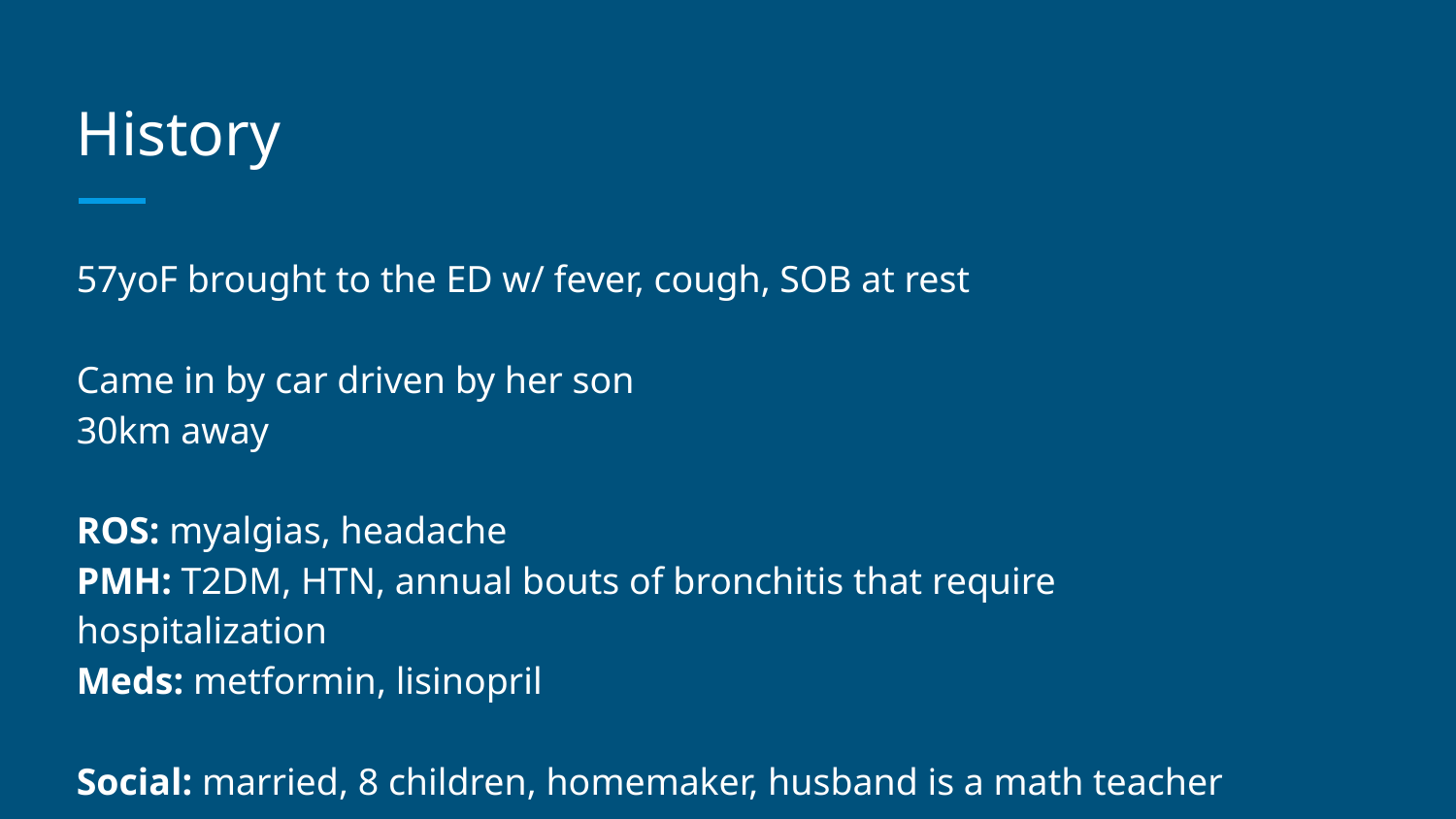

# History
57yoF brought to the ED w/ fever, cough, SOB at rest
Came in by car driven by her son
30km away
ROS: myalgias, headache
PMH: T2DM, HTN, annual bouts of bronchitis that require hospitalization
Meds: metformin, lisinopril
Social: married, 8 children, homemaker, husband is a math teacher

## Slide 5
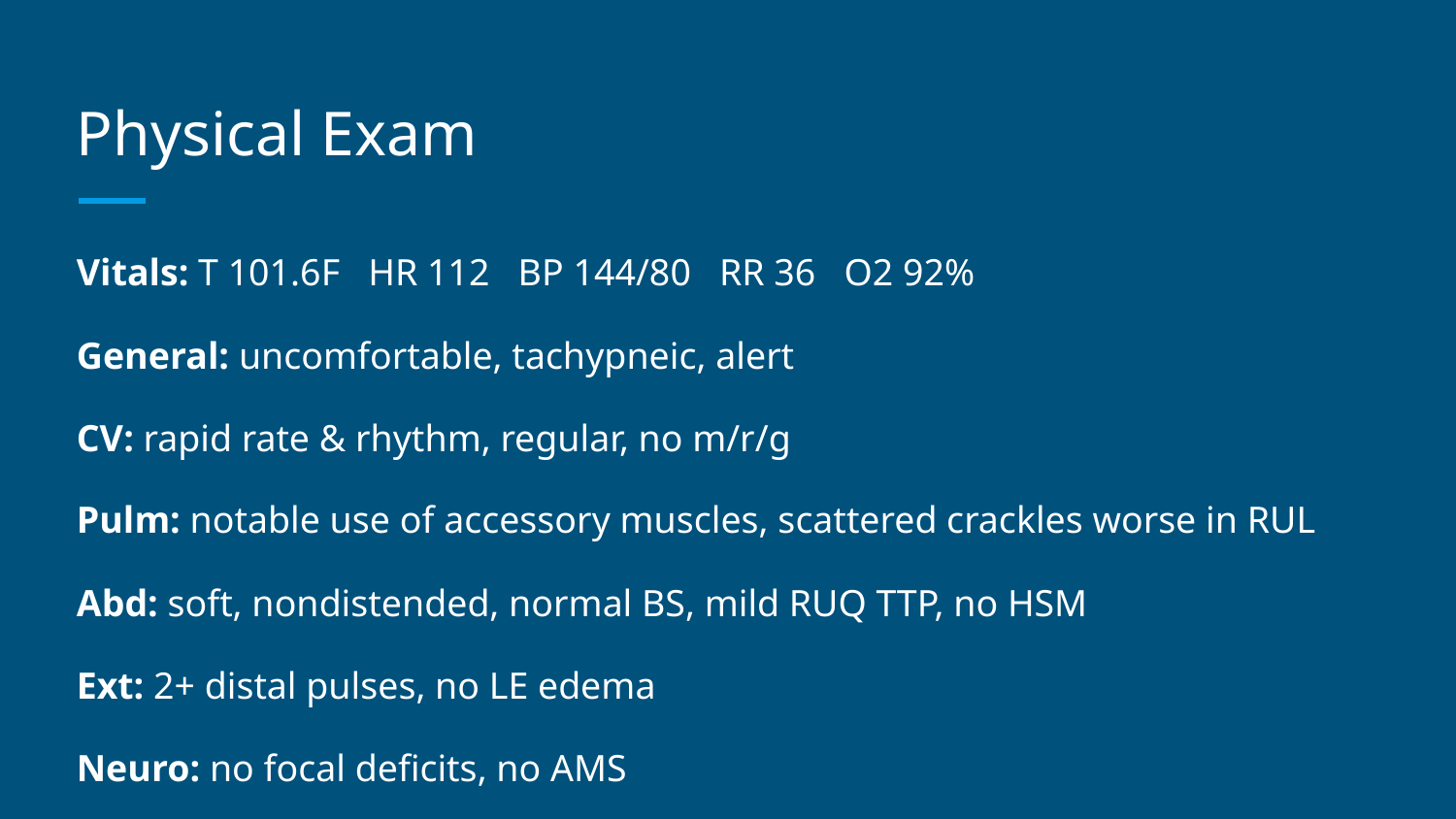

# Physical Exam
Vitals: T 101.6F HR 112 BP 144/80 RR 36 O2 92%
General: uncomfortable, tachypneic, alert
CV: rapid rate & rhythm, regular, no m/r/g
Pulm: notable use of accessory muscles, scattered crackles worse in RUL
Abd: soft, nondistended, normal BS, mild RUQ TTP, no HSM
Ext: 2+ distal pulses, no LE edema
Neuro: no focal deficits, no AMS

## Slide 6
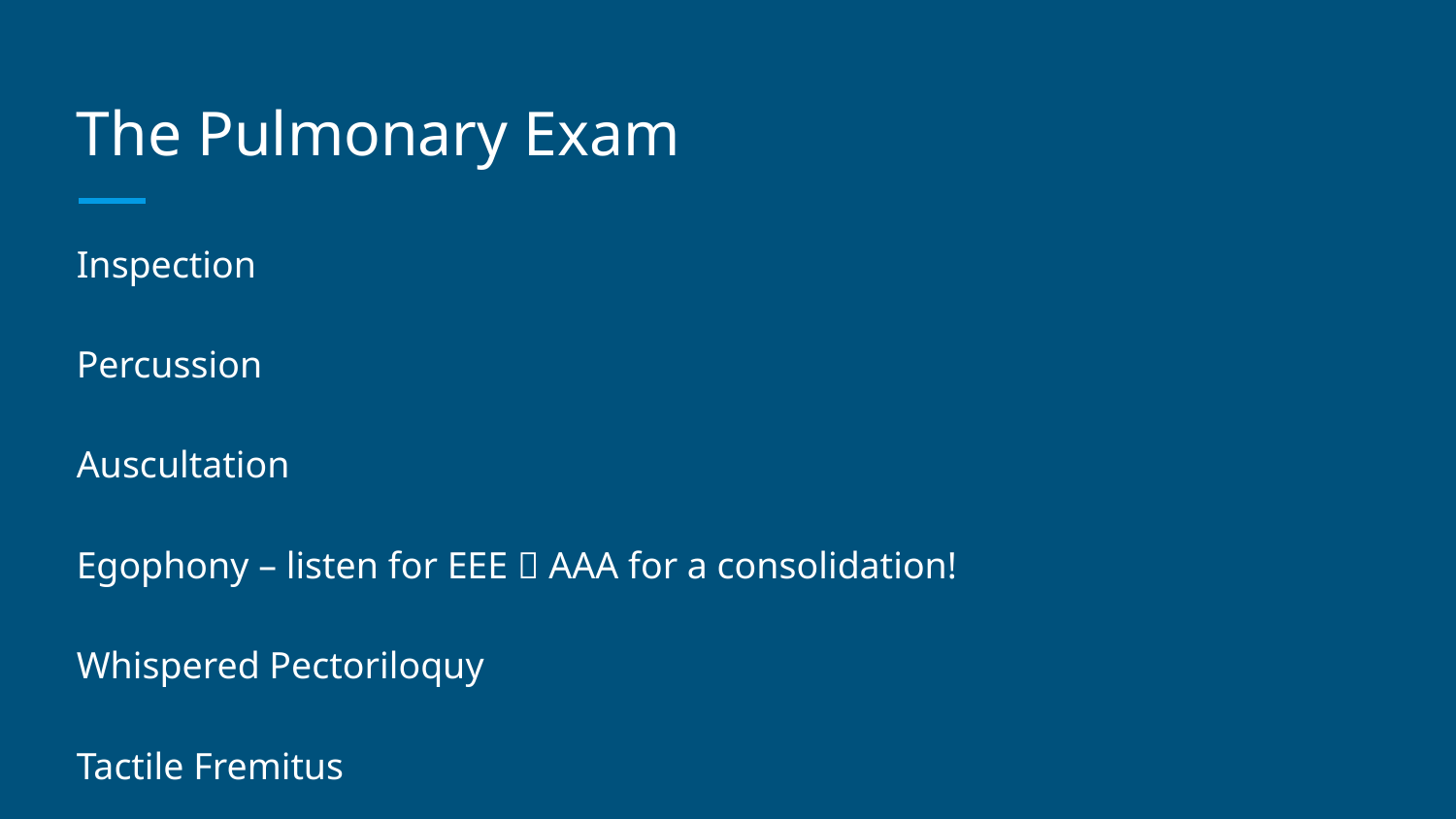

# The Pulmonary Exam
Inspection
Percussion
Auscultation
Egophony – listen for EEE  AAA for a consolidation!
Whispered Pectoriloquy
Tactile Fremitus

## Slide 7
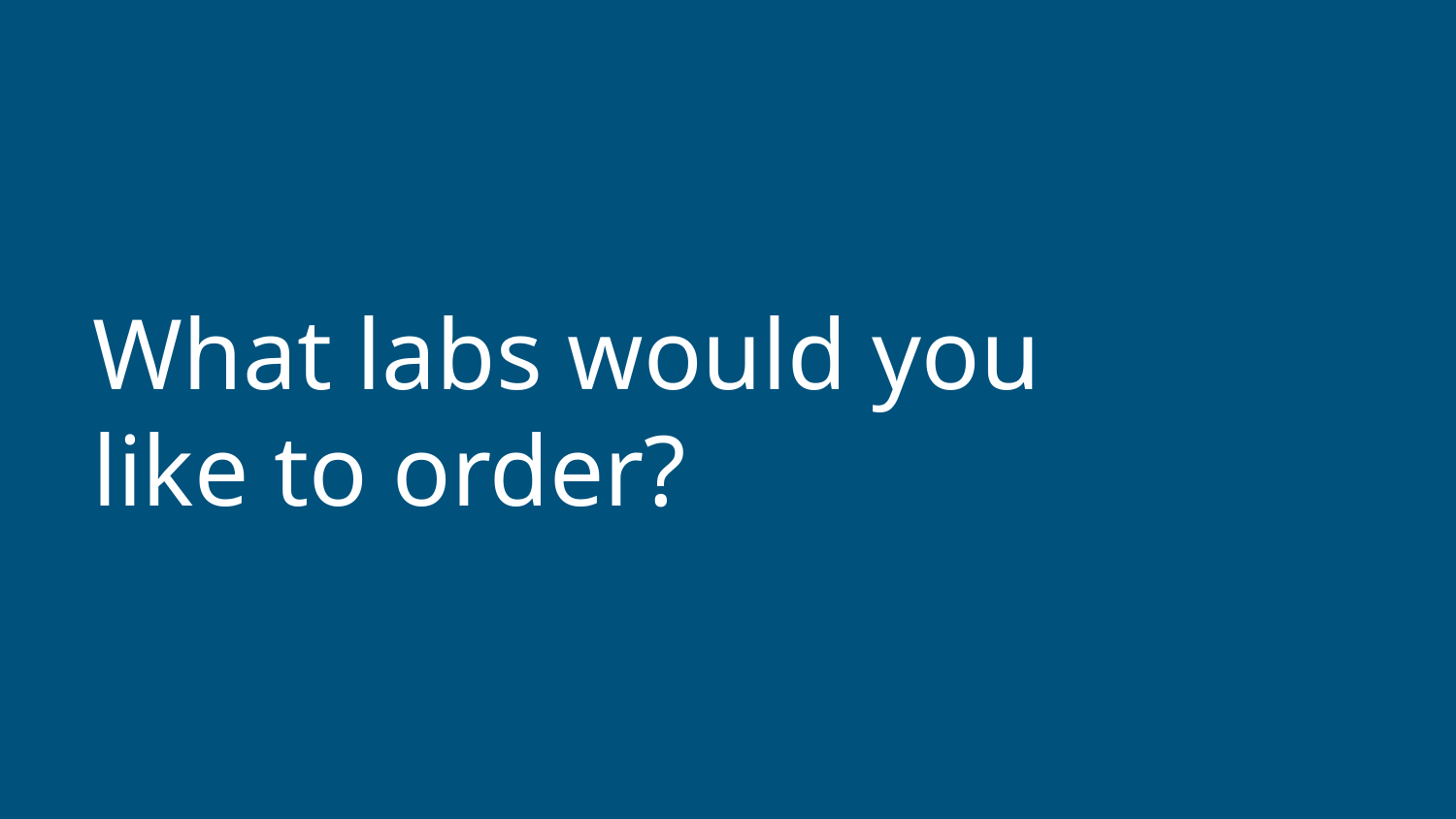

# What labs would you like to order?

## Slide 8
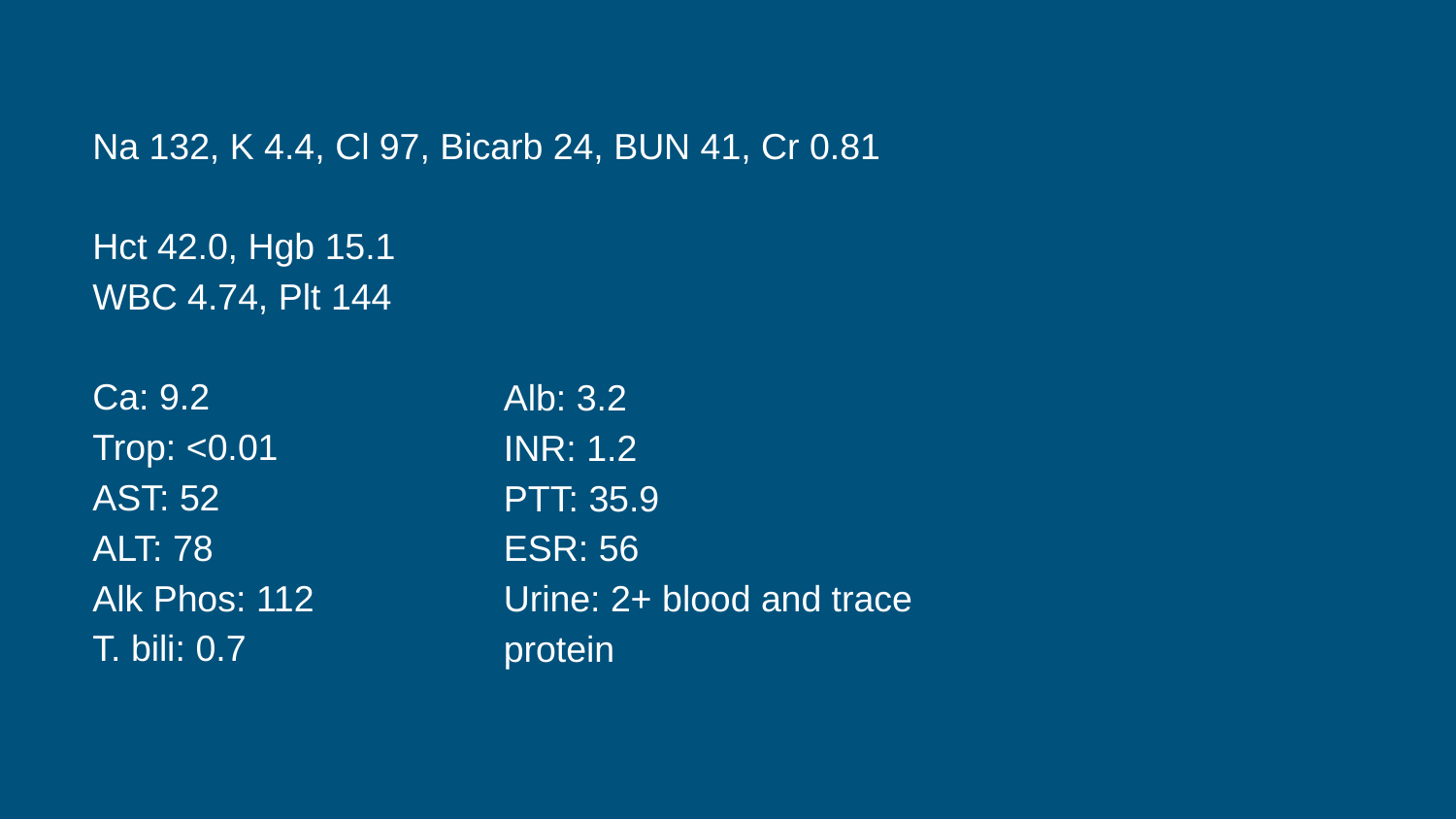

# Na 132, K 4.4, Cl 97, Bicarb 24, BUN 41, Cr 0.81
Hct 42.0, Hgb 15.1
WBC 4.74, Plt 144
Ca: 9.2
Trop: <0.01
AST: 52
ALT: 78
Alk Phos: 112
T. bili: 0.7
Alb: 3.2
INR: 1.2
PTT: 35.9
ESR: 56
Urine: 2+ blood and trace protein

## Slide 9
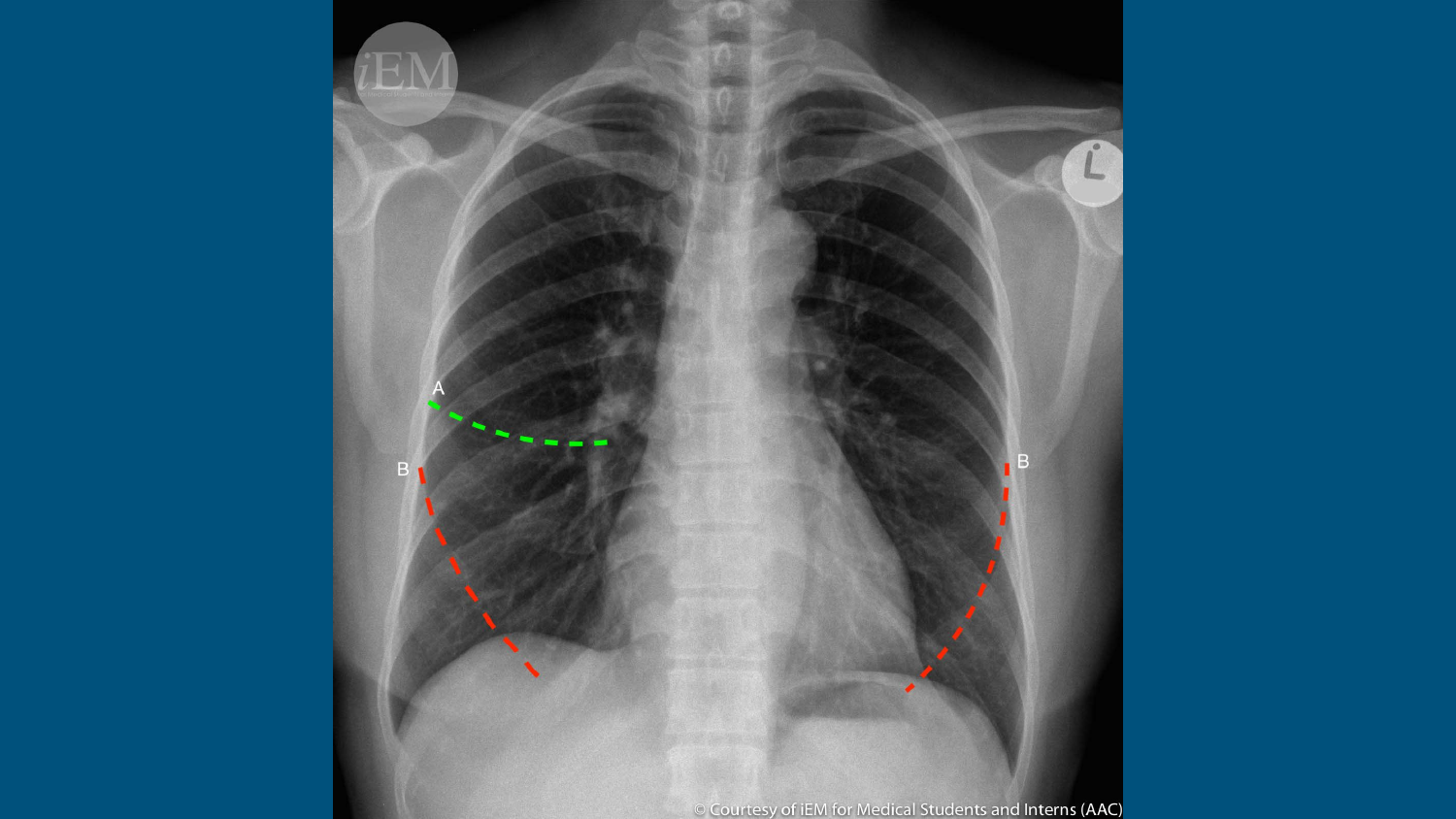

## Slide 10
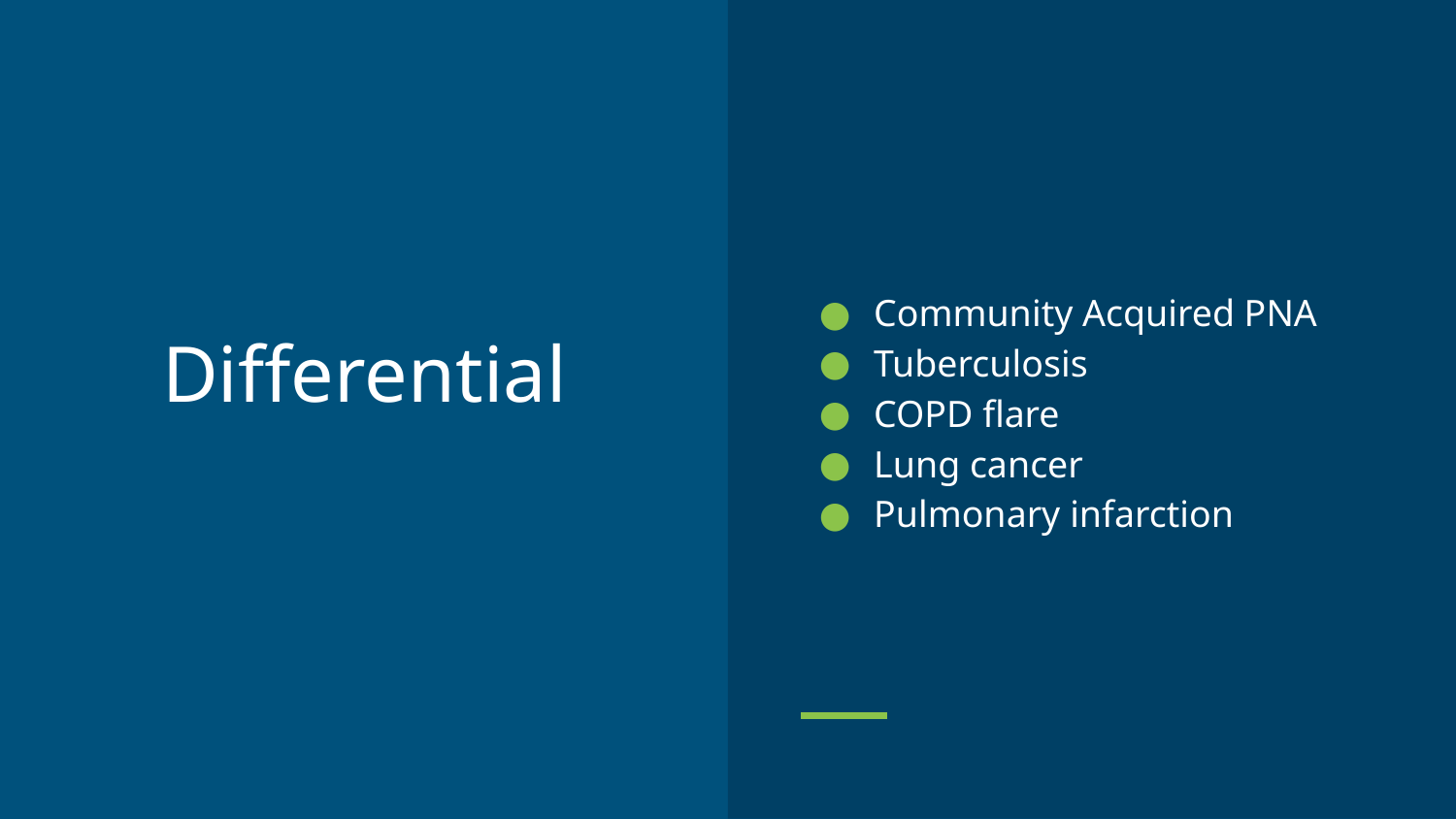

Community Acquired PNA
Tuberculosis
COPD flare
Lung cancer
Pulmonary infarction
# Differential

## Slide 11
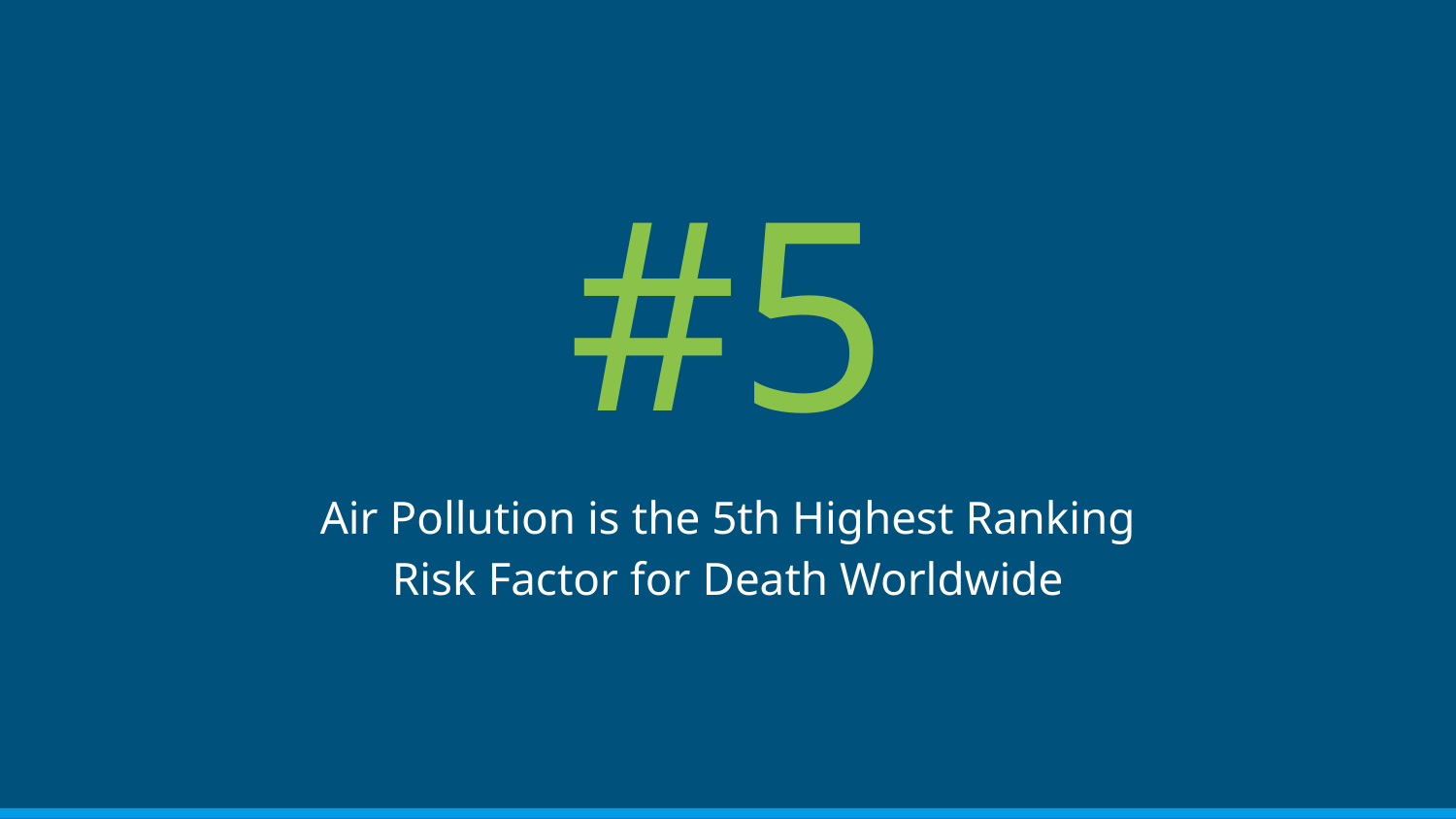

# #5
Air Pollution is the 5th Highest Ranking
Risk Factor for Death Worldwide

## Slide 12
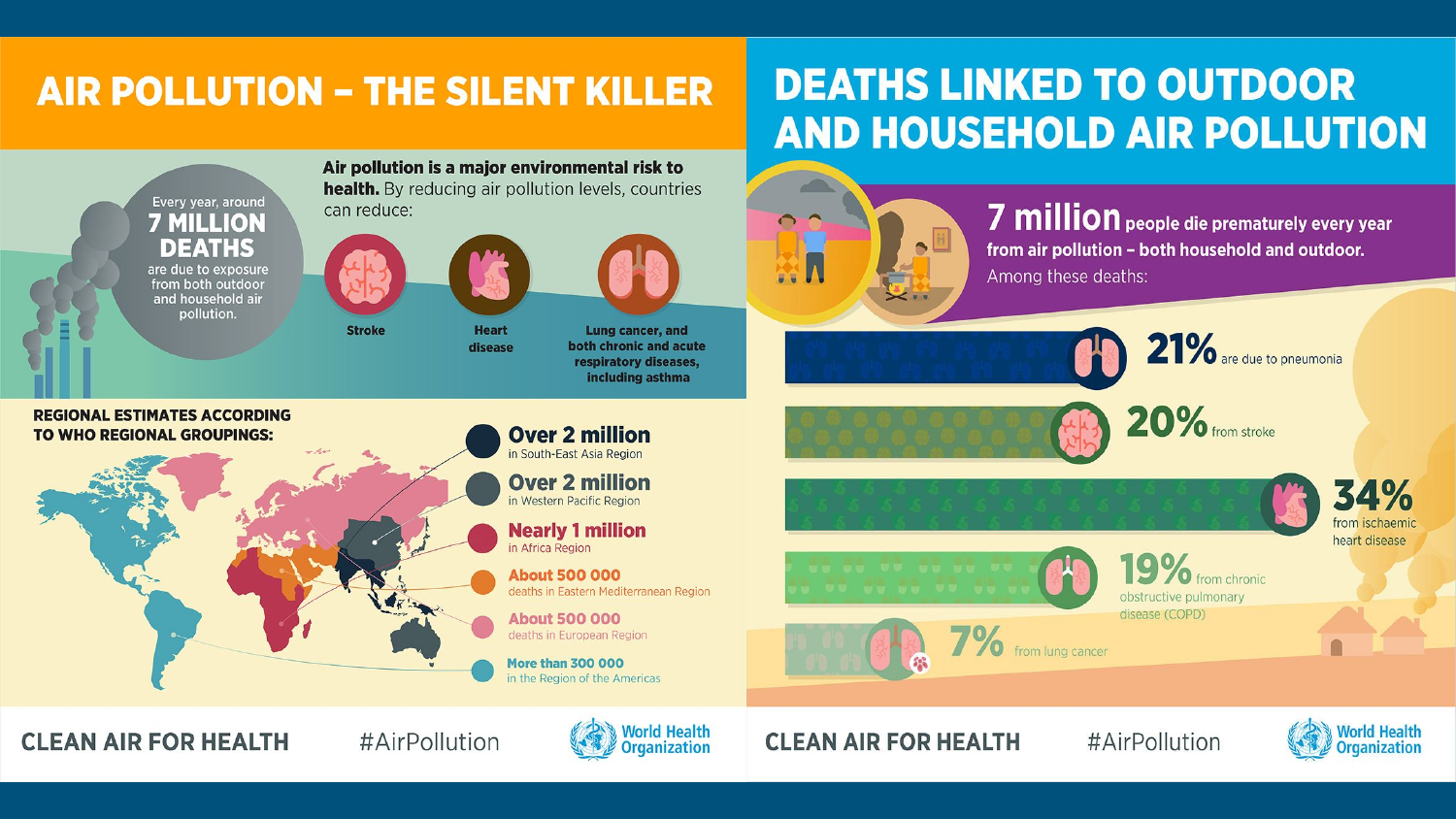

## Slide 13
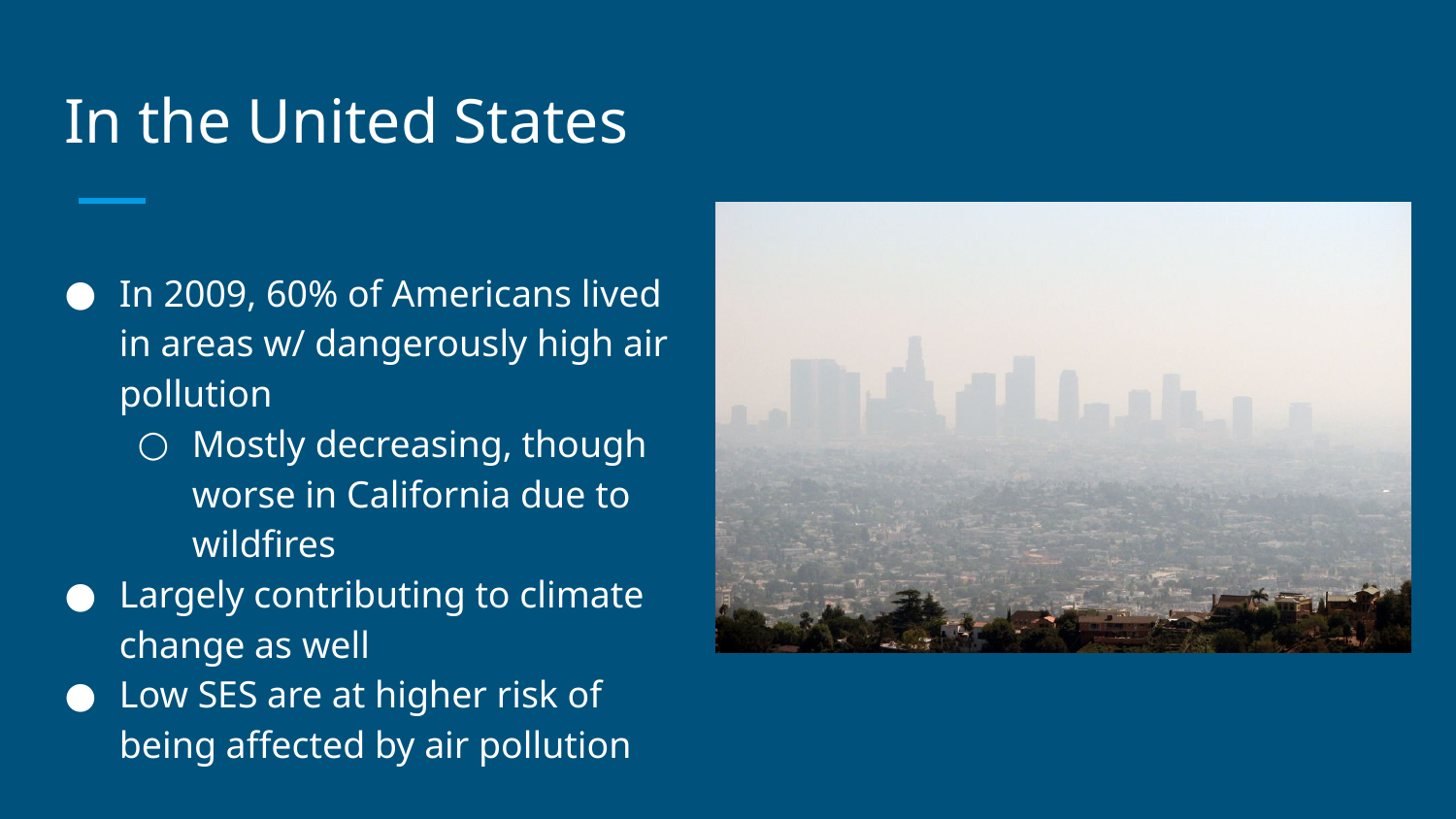

# In the United States
In 2009, 60% of Americans lived in areas w/ dangerously high air pollution
Mostly decreasing, though worse in California due to wildfires
Largely contributing to climate change as well
Low SES are at higher risk of being affected by air pollution

## Slide 14
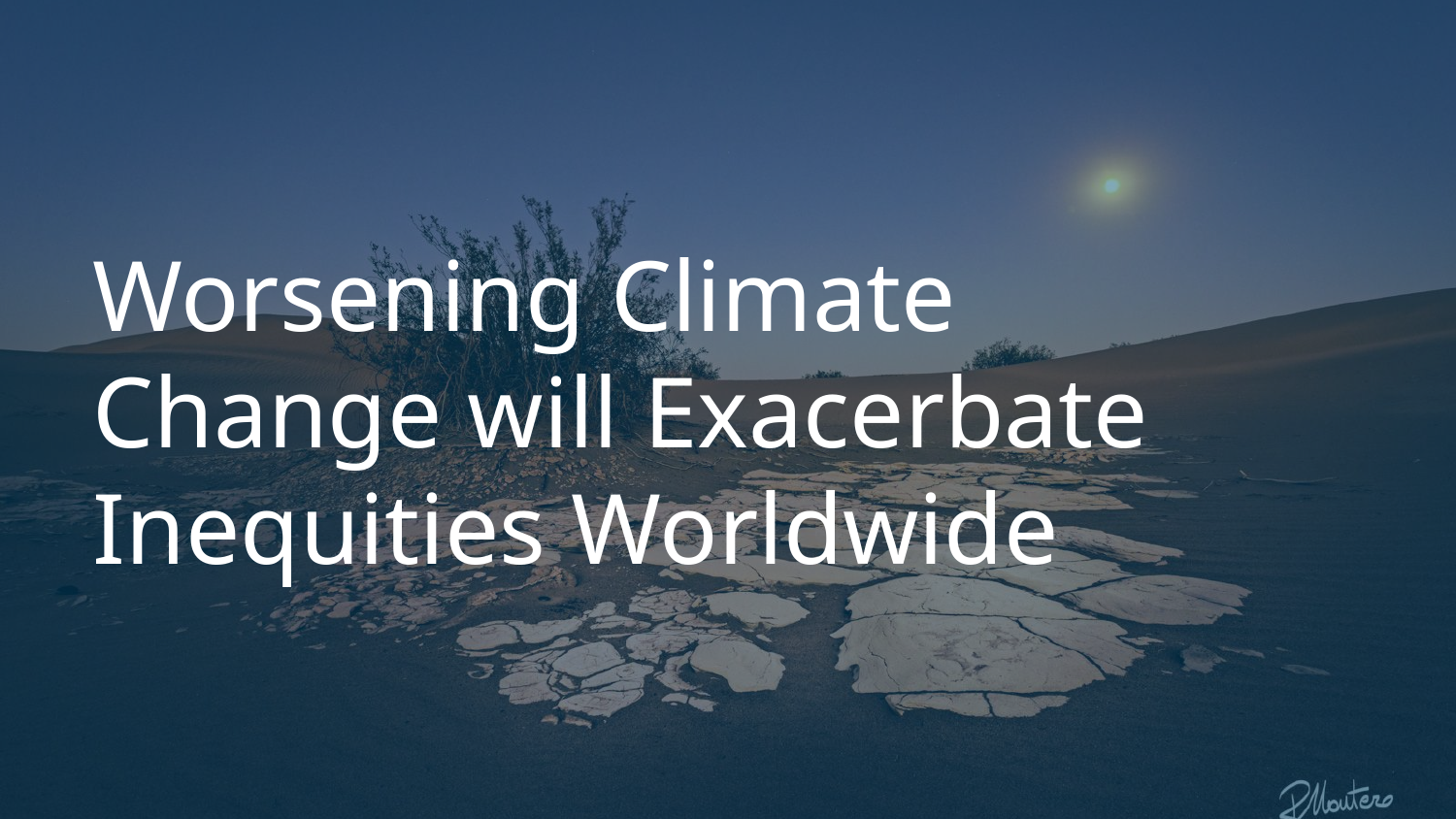

# Worsening Climate Change will Exacerbate Inequities Worldwide

## Slide 15
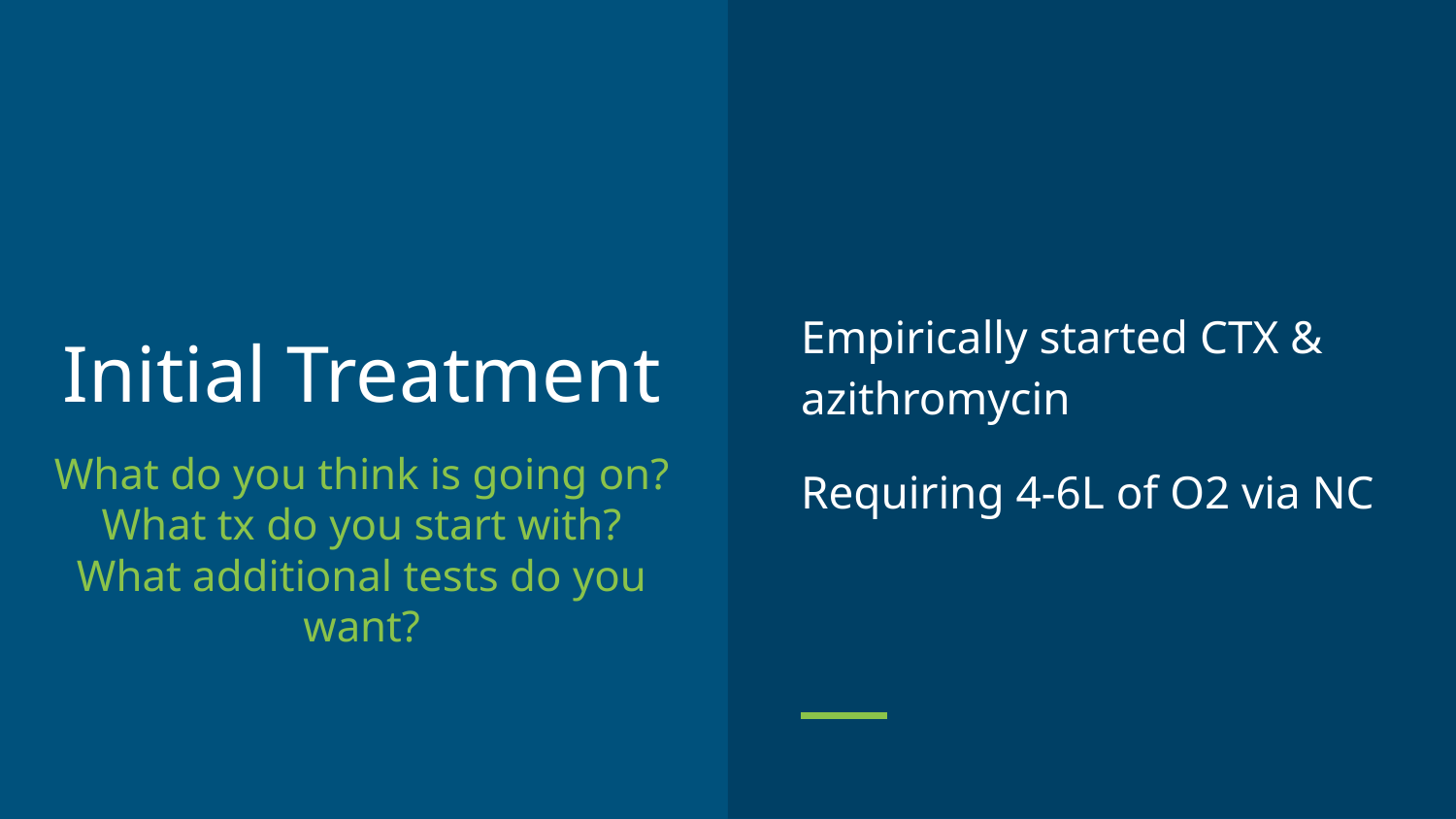

Empirically started CTX & azithromycin
Requiring 4-6L of O2 via NC
# Initial Treatment
What do you think is going on?
What tx do you start with?
What additional tests do you want?

## Slide 16
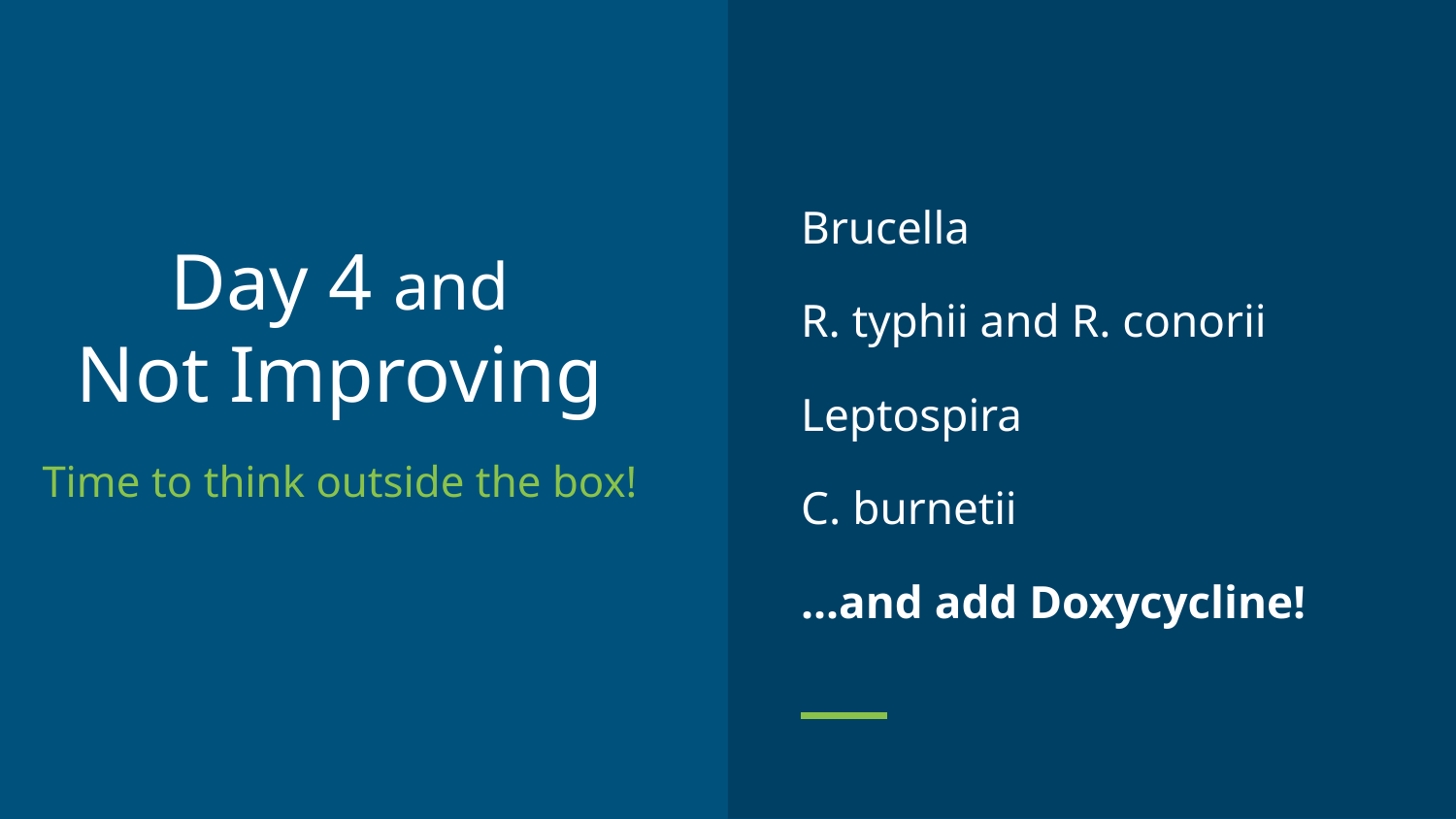

Brucella
R. typhii and R. conorii
Leptospira
C. burnetii
...and add Doxycycline!
# Day 4 and Not Improving
Time to think outside the box!

## Slide 17
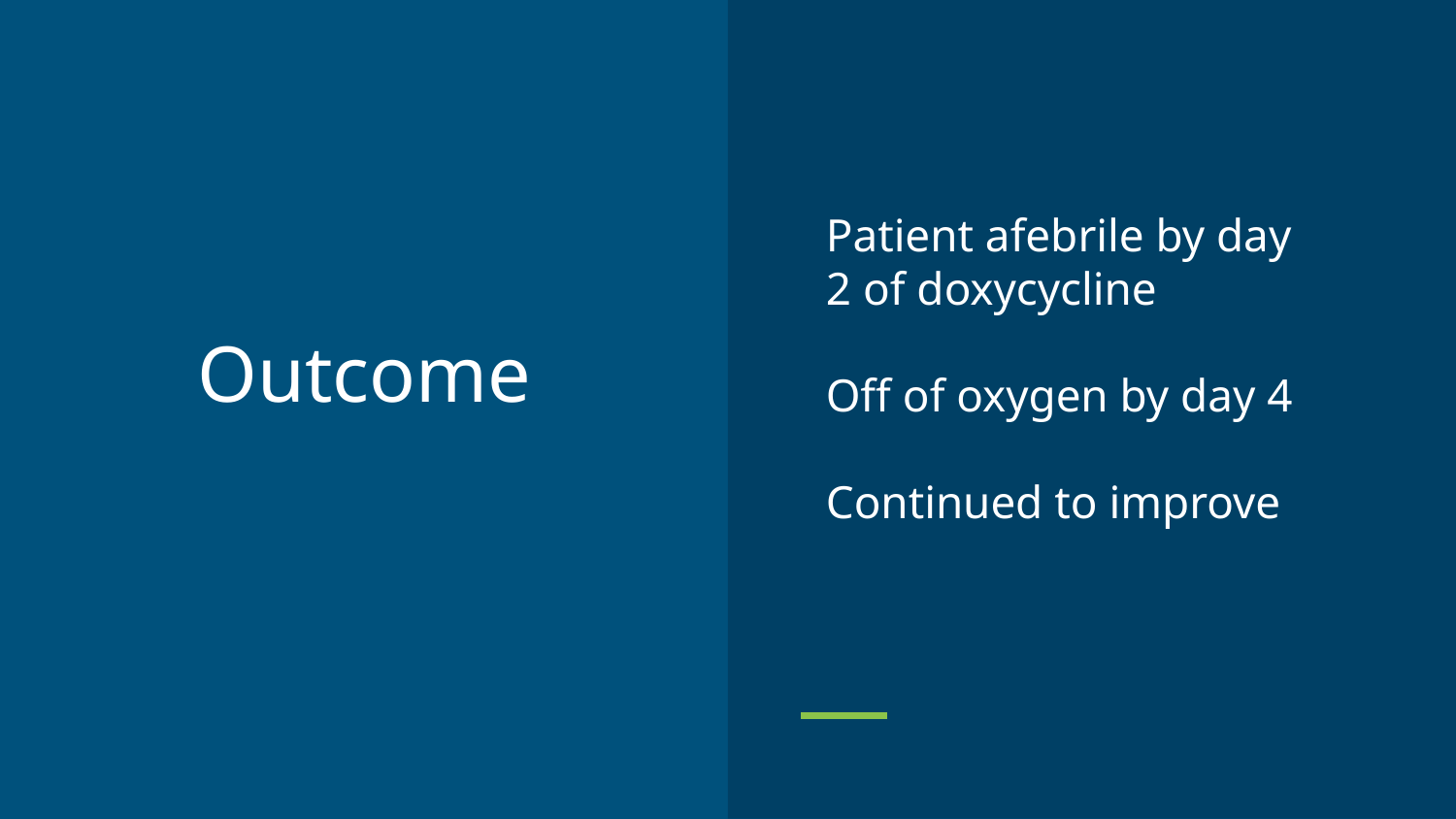

# Outcome
Patient afebrile by day 2 of doxycycline
Off of oxygen by day 4
Continued to improve

## Slide 18
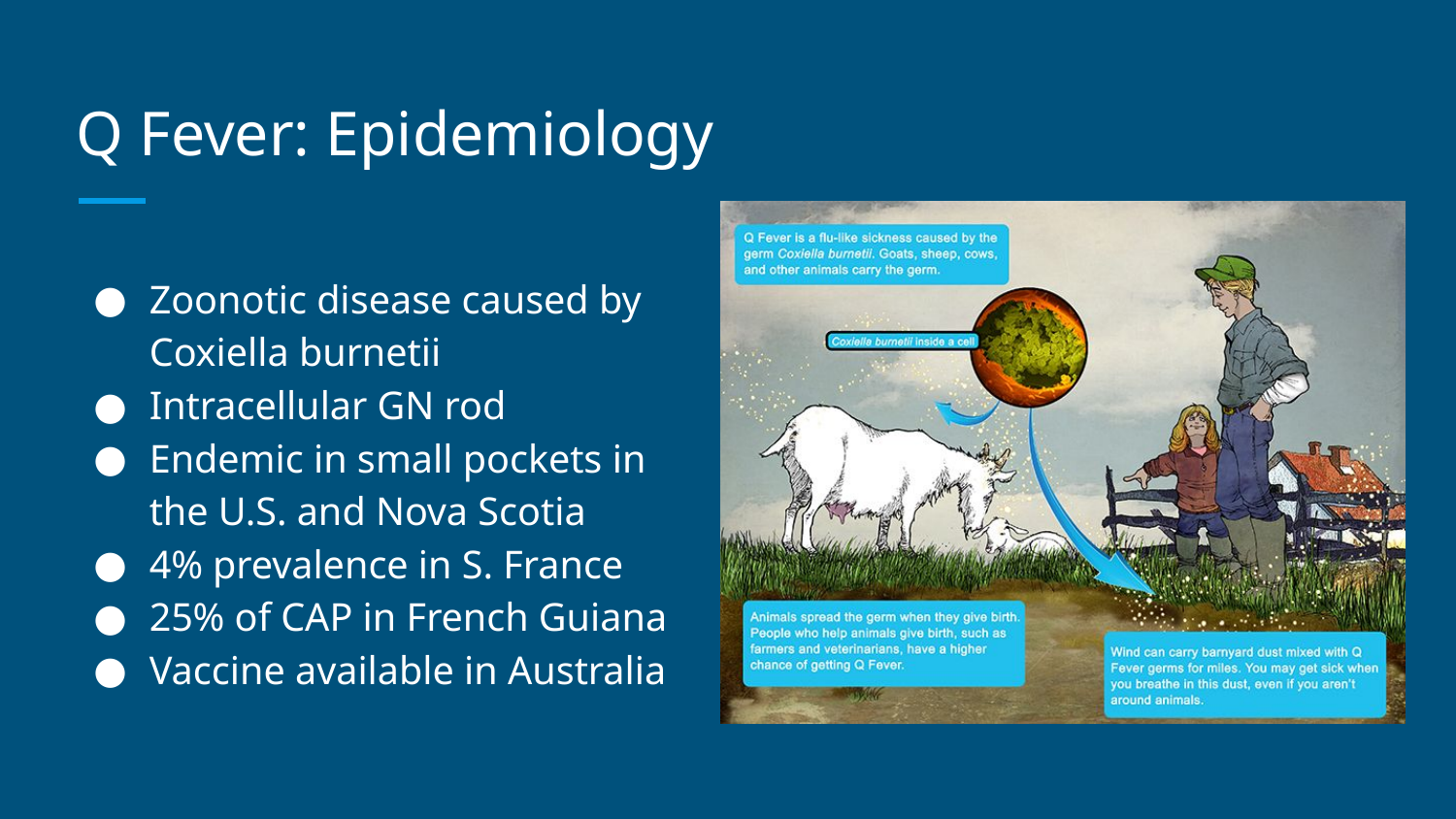

# Q Fever: Epidemiology
Zoonotic disease caused by Coxiella burnetii
Intracellular GN rod
Endemic in small pockets in the U.S. and Nova Scotia
4% prevalence in S. France
25% of CAP in French Guiana
Vaccine available in Australia

## Slide 19
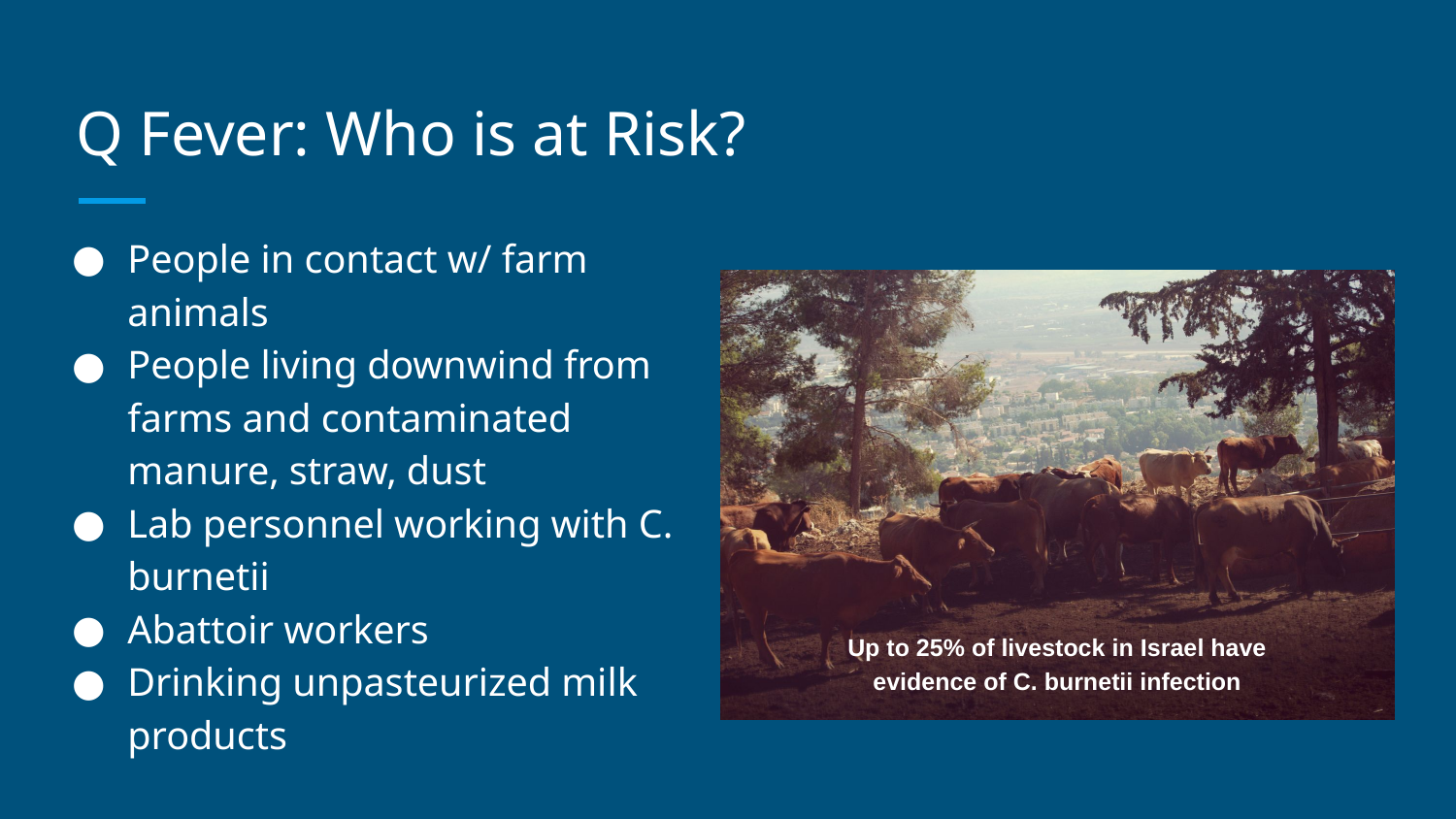

# Q Fever: Who is at Risk?
People in contact w/ farm animals
People living downwind from farms and contaminated manure, straw, dust
Lab personnel working with C. burnetii
Abattoir workers
Drinking unpasteurized milk products
Up to 25% of livestock in Israel have evidence of C. burnetii infection

## Slide 20
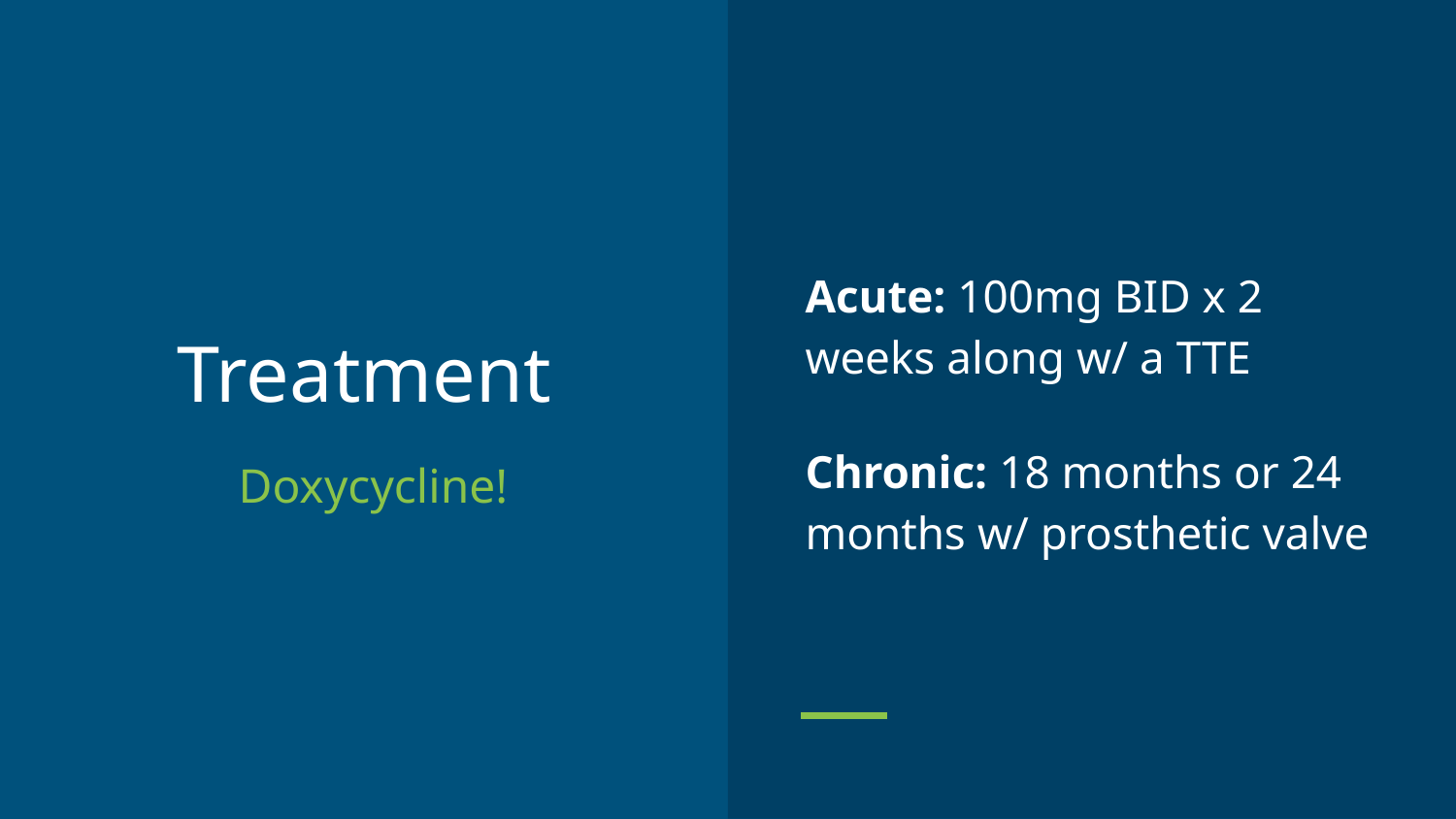

Acute: 100mg BID x 2 weeks along w/ a TTE
Chronic: 18 months or 24 months w/ prosthetic valve
# Treatment
Doxycycline!

## Slide 21
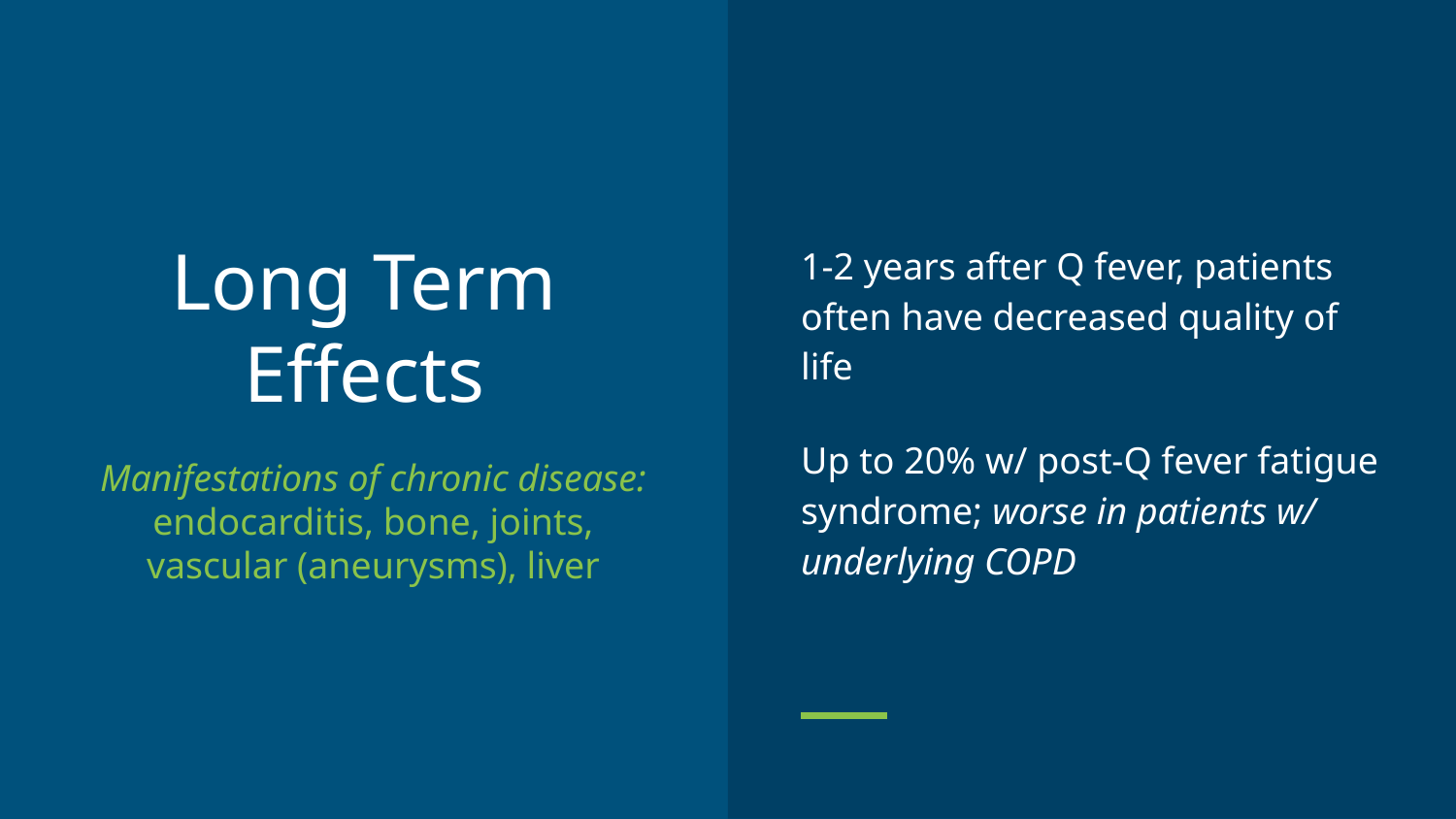

1-2 years after Q fever, patients often have decreased quality of life
Up to 20% w/ post-Q fever fatigue syndrome; worse in patients w/ underlying COPD
# Long Term Effects
Manifestations of chronic disease:
endocarditis, bone, joints,
vascular (aneurysms), liver

## Slide 22
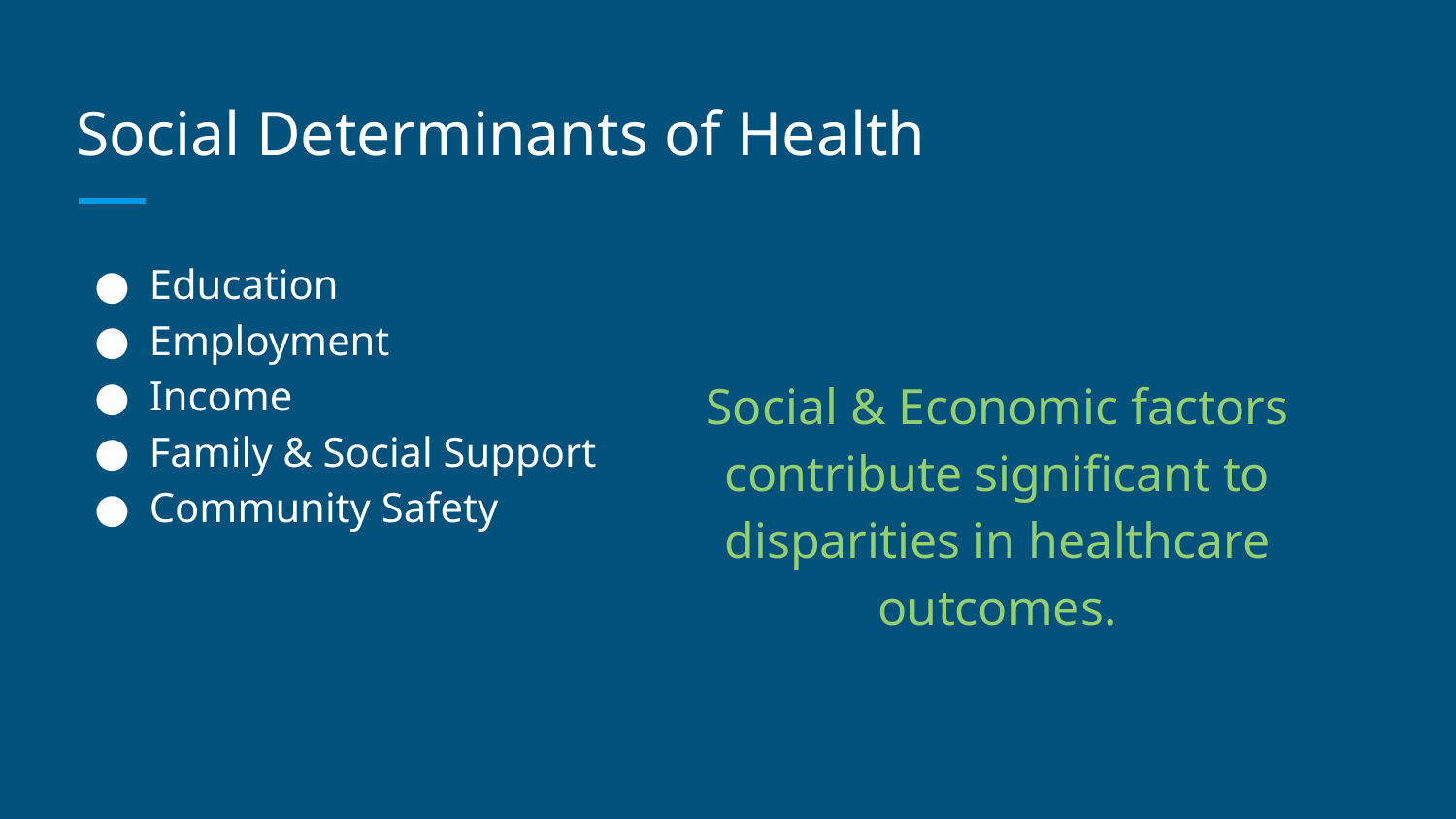

# Social Determinants of Health
Education
Employment
Income
Family & Social Support
Community Safety
Social & Economic factors contribute significant to disparities in healthcare outcomes.

## Slide 23
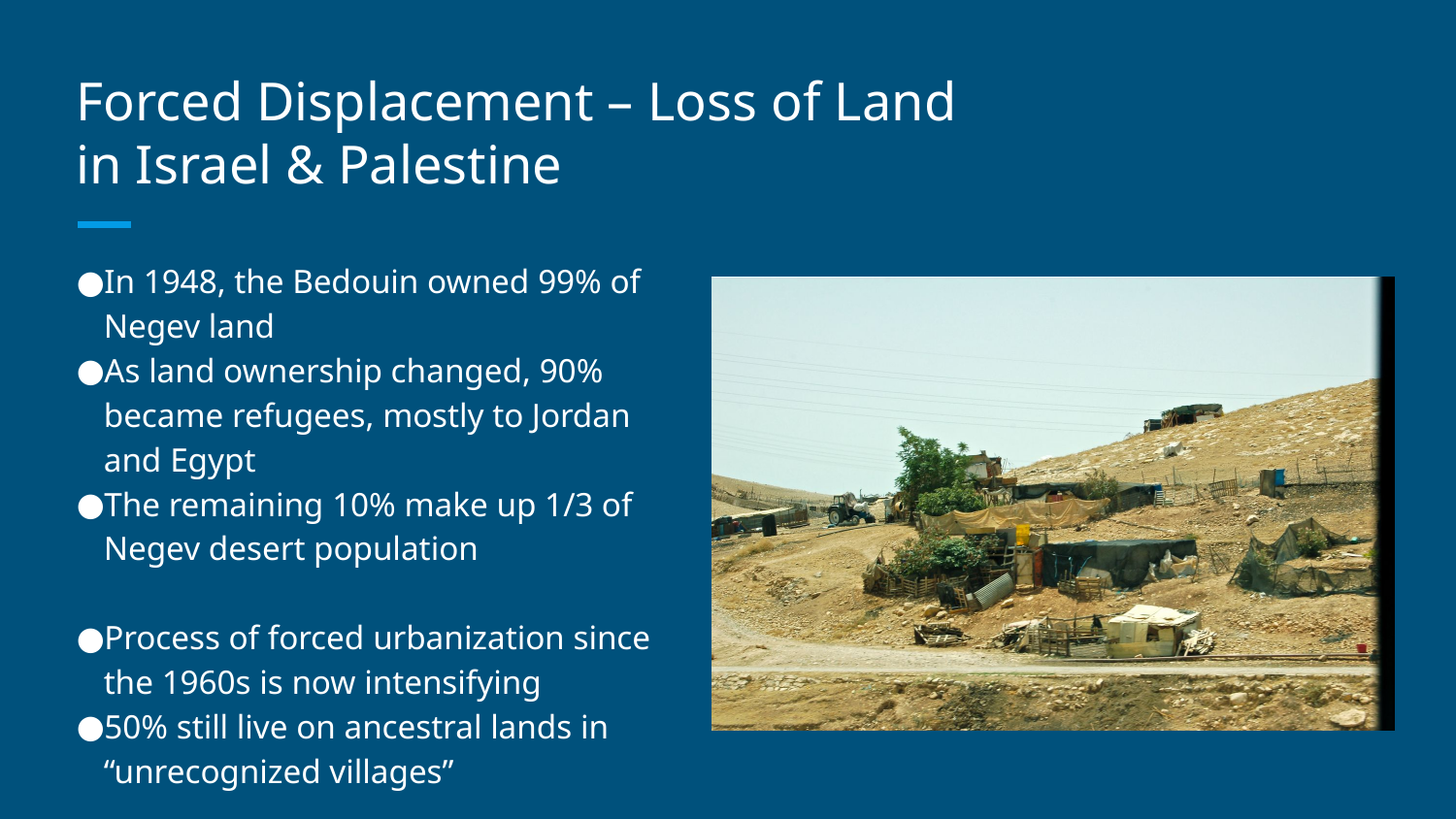

# Forced Displacement – Loss of Land in Israel & Palestine
In 1948, the Bedouin owned 99% of Negev land
As land ownership changed, 90% became refugees, mostly to Jordan and Egypt
The remaining 10% make up 1/3 of Negev desert population
Process of forced urbanization since the 1960s is now intensifying
50% still live on ancestral lands in “unrecognized villages”

## Slide 24
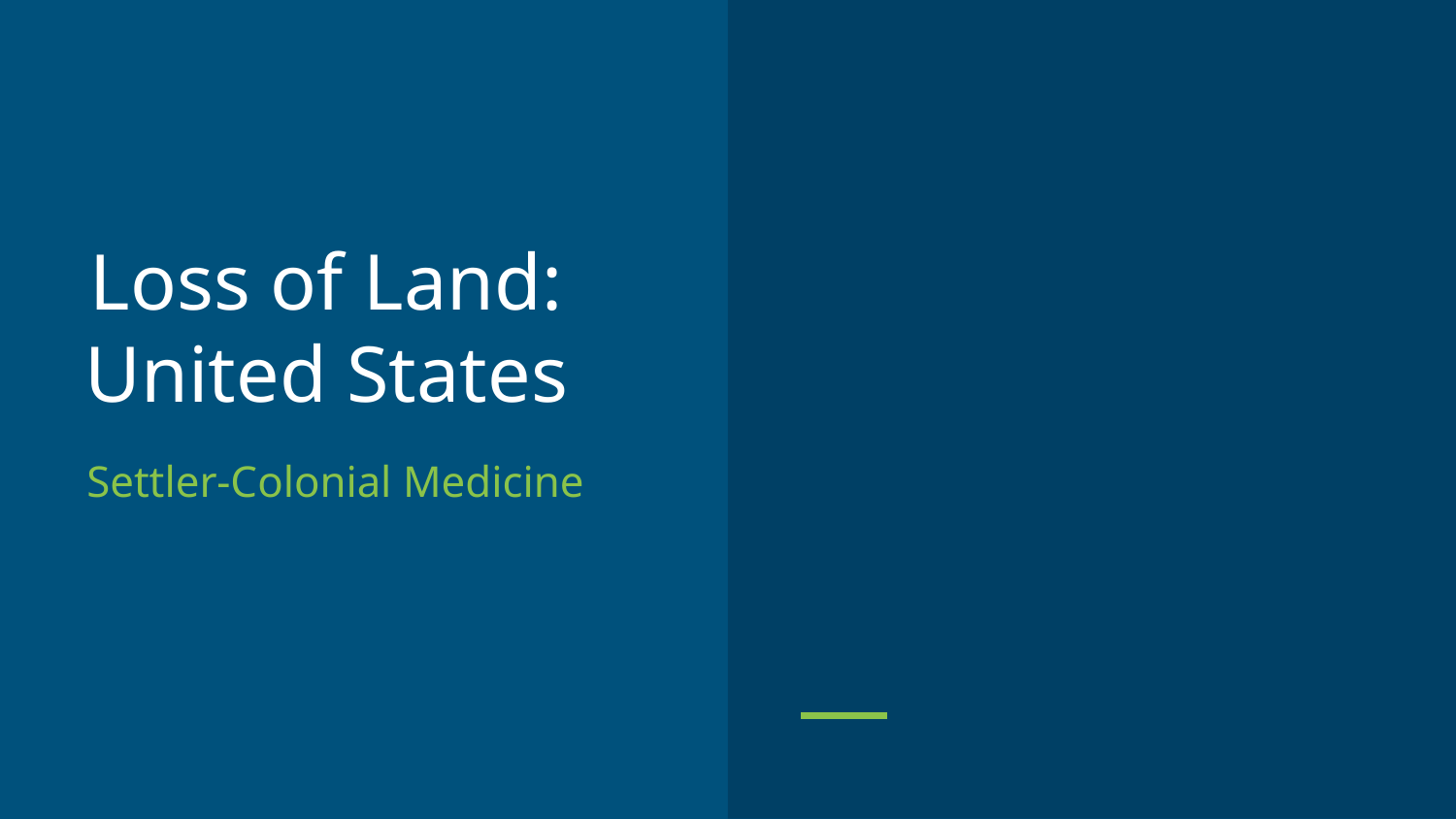

# Loss of Land: United States
Settler-Colonial Medicine

## Slide 25
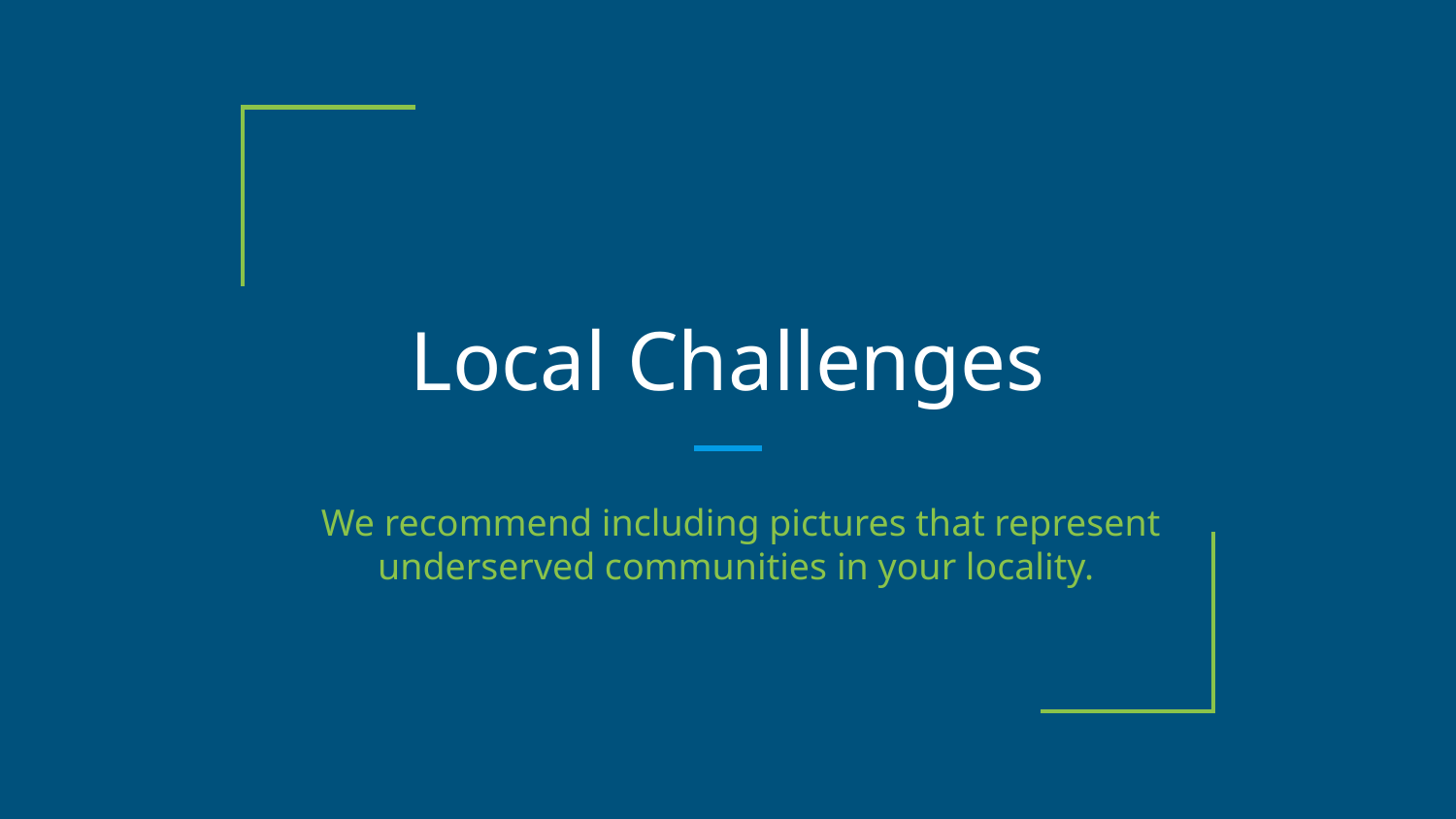

# Local Challenges
We recommend including pictures that represent underserved communities in your locality.

## Slide 26
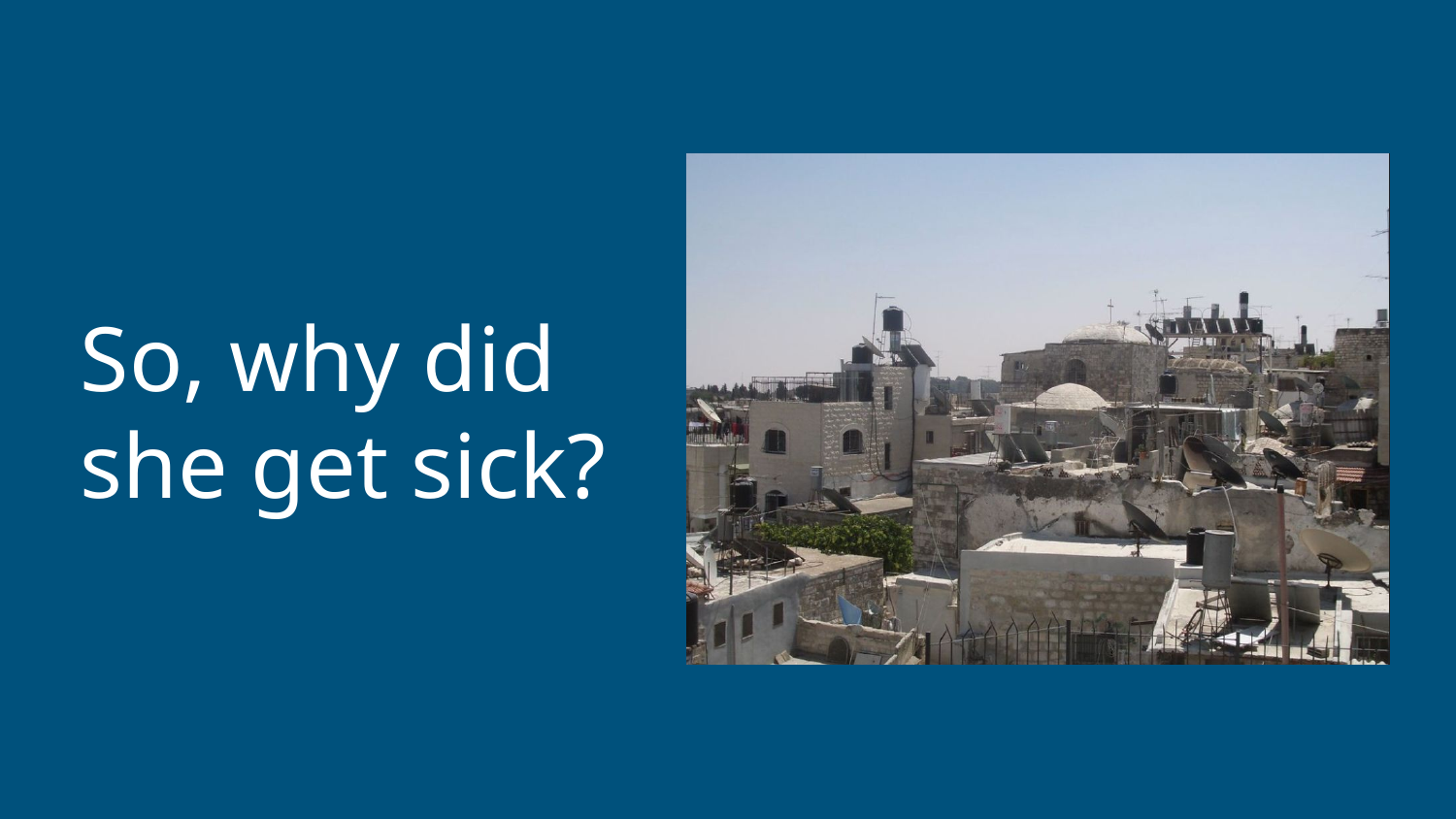

# So, why did she get sick?

## Slide 27
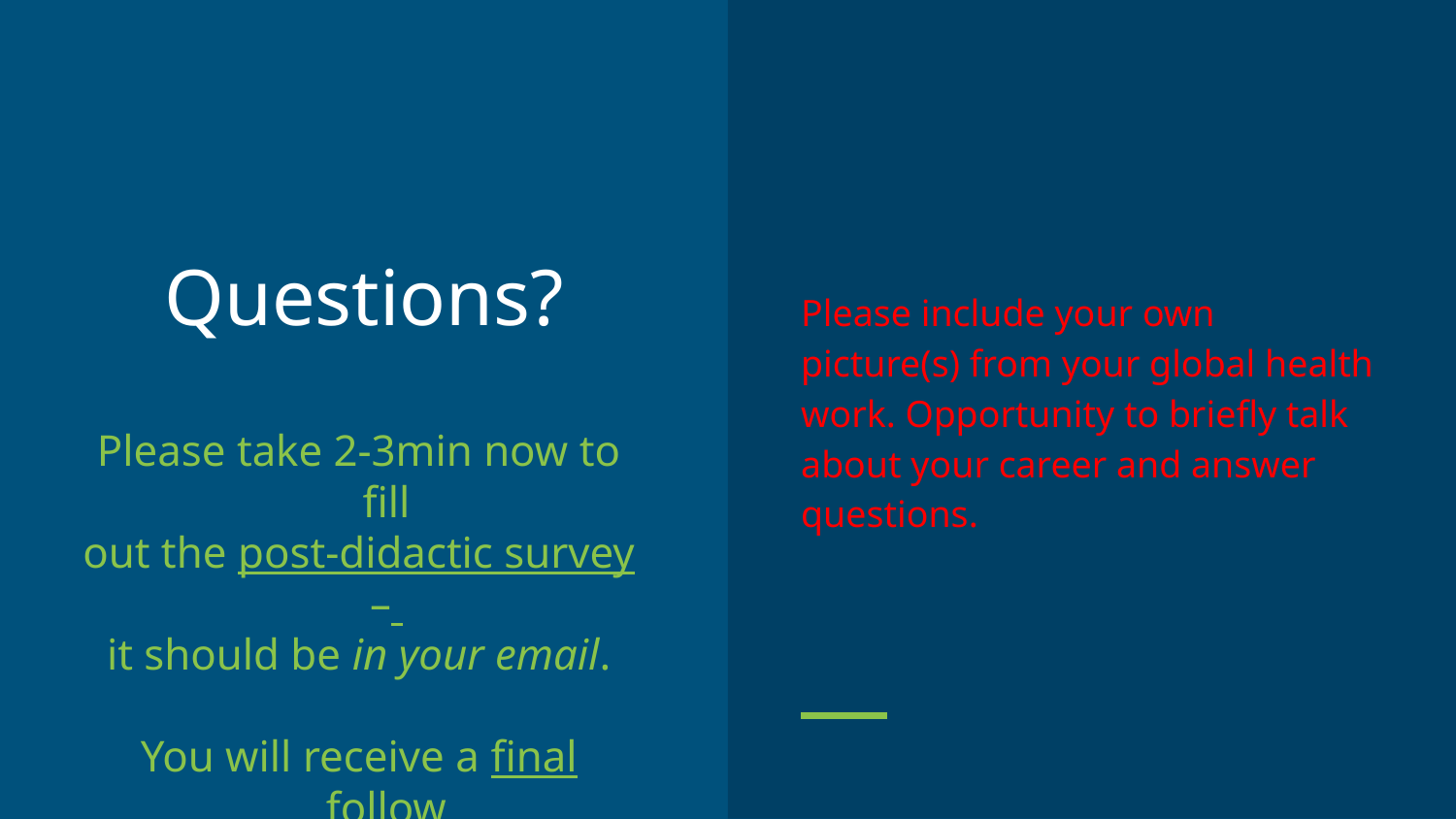

# Questions?
Please include your own picture(s) from your global health work. Opportunity to briefly talk about your career and answer questions.
Please take 2-3min now to fill
out the post-didactic survey –
it should be in your email.
You will receive a final follow
up survey in 2 weeks.
