# Supplementary material for: A Longitudinal Case-Based Global Health Curriculum for the Medical Student Clerkship Year
Source: MedEdPORTAL. 2020 Dec 8;16:11038. doi: 10.15766/mep_2374-8265.11038 (PMC7732136; doi:10.15766/mep_2374-8265.11038)
Supplement: Supplementary file 1 — Clerkship Director Proposal.pptxProject Description.docxPediatrics GH Didactic.pptxSurgery GH Didactic.pptxMedicine GH Didactic.pptxFacilitator Notes.docxPredidactic Survey.docxPostdidactic Survey.docxFollow-up Survey.docx [file mep_2374-8265.11038-s001.zip › F. Facilitator Notes.docx]

**Facilitator Notes for Didactic Sessions**

Pediatrics & Global Health

1. **Slide 1**
   1. None
2. **Slide 2**
   1. This slide includes a description of the setting (Syrian refugee camp), including living conditions and available healthcare resources. This helps frame the way students will approach the case throughout the session.
3. **Slide 3**
   1. This is a good point to pause and ask the students in the room about their prior experiences in global health and general clinical care:
      1. Has anyone done global health work before?
      2. Have you taken care of patients with diarrhea? Either here or elsewhere?
      3. What happened in that case?
4. **Slide 4**
   1. This is an opportunity to help students build on their history and observation skills. You can ask students the following:
      1. Does anything in particular from the history stand out?
      2. What physical exam would you want to do based on this history?
5. **Slide 5**
   1. This is an opportunity to help students build on their physical exam skills. You can ask students the following:
      1. What aspects of this physical exam are concerning?
      2. What do you (students) think overall is going on?
   2. You can also discuss that children have increased insensible losses and higher metabolic rates, both of which make them more susceptible to dehydration.
6. **Slide 6**
   1. This is an opportunity to discuss growth curves, including normal growth curves versus malnutrition. You can start by posing the questions above to allow students the opportunity to think through growth curves and what to expect. For reference, the average weight for a 4 year old boy is 35 lbs. The boy in this case is only 20 lbs.
7. **Slide 7**
   1. Here you can discuss a physical exam technique (MUAC) that students don’t often use in high-income countries, but is critical in assessing malnutrition status in children. You can start off by asking students the following, before then discussing what MUAC is.
      1. Have any of you heard of Mid-Upper Arm Circumference before?
      2. Have any of you ever used it on a patient?
   2. More about MUAC:
      1. It is often used in settings such as refugee camps to classify severity of malnutrition (a quick triage tool). It is:
      2. Easy to perform: limp arm, take midpoint between tip of shoulder to tip of elbow, wrap MUAC tape around midpoint, and read number
      3. Can be done by community health workers
      4. Best predictor of child mortality in children 1yo-5yo
      5. MUAC <11.0cm = acute severe malnutrition and associated with high mortality
   3. We recommend bringing pre-cut string that is 11cm long (ideally one for each student) so that students can better understand what severe malnutrition really looks like. This exercise was mentioned in feedback as being very impactful on the students.
8. **Slide 8**
   1. This slide is meant to show the severe impact of malnutrition, which is implicated in 50% of under 5 mortality.
9. **Slide 9**
   1. This slide is meant to engage students in a discussion on malnutrition and how to classify different forms (Kwashiorkor vs. Marasmus). You can start by asking students the following:
      1. What leads to malnutrition?
      2. What are the structural causes?
      3. Have you seen here in the U.S.?
      4. What are the differences between Kwashiorkor & Marasmus?
      5. How does malnutrition influence treatment of diarrheal illness?
10. **Slide 10**
    1. This slide is an opportunity to discuss what labs students would normally want to order in a high-resource setting for a pediatric patient with significant diarrhea. Start off by posing the question above to the students and allowing them to think through this. If there is time, you can push students to explain why they would want certain labs. We have included sample lab values below to write on a white board or just to share out loud prior to moving on from this slide. We have also included why students may expect these particular labs, to help facilitate their clinical learning. *This is also an important place to discuss what labs would or wouldn’t be available in a low-resource setting, and how you would approach this case differently because of that.*
       1. **CBC:** Hgb 10, Hct 30, WBC 13K, Plt 300K
          **BMP:** Na 132, K 3.6, Cl 98, Bicarb 16, BUN 63, Cr 1.3
          → Dehydration due to significant volume loss
          → Non anion gap metabolic acidosis
          → Hypovolemic hyponatremia + hypokalemia
          → slight hemoconcentration
11. **Slide 11**
    1. This slide is to encourage students to think about the differential for the patient’s diarrhea. The above classification aligns with the WHO classification, which can be found here: <https://www.who.int/news-room/fact-sheets/detail/diarrhoeal-disease>
12. **Slide 12**
    1. This slide is to encourage students to think about Rotavirus specifically, what it is, how it spreads, and its effects. Based on how much time you have left in the session, you can spend more or less time on this slide.
13. **Slide 13**
    1. Oral Rehydration Solution (ORS) is an important component of treatment for diarrheal disease around the world, though is underutilized in high-resource settings like the U.S. This is an important opportunity to both discuss ORS use clinically, but also its importance as a cost-effective way to address dehydration, morbidity, and mortality from diarrheal disease. This slide includes information about how to make ORS and how to administer it. It also includes when you may decide IV fluids are necessary. Below are some additional points to include, and also “other treatments” to briefly discuss with students
    2. Additional points to make:
       1. Underused in low-income countries, where it could prevent deaths from diseases such as rotavirus, cholera, and other acute watery diarrheal disease
       2. Underused in high-income countries, where it could reduce healthcare costs
    3. Other treatments to discuss:
       1. ABX – only in following situations:
          1. co-morbid conditions, e.g. malnourished, immune compromised
          2. high suspicion of cholera (best rx is single dose azithro, lots resistance to tetracyclines and cipro)
          3. concern for systemic infection (e.g. Shigella rx’d w/ 3d cipro or 5d azithro, Salmonella, amoebic dysentery metronidazole 7-10d)
          4. chronic infection (e.g. giardiasis)
       2. No anti-diarrheal medications
       3. Early refeeding (1-2tsp veg oil per feed, feed up to 6xs daily)
       4. Vitamins and minerals – zinc (10mg/d for kids <6mo, 20mg for >6mos for 10d) for 10-14 d can reduce quantity of diarrhea
14. **Slide 14**
    1. This slide is an opportunity to discuss the typical outcomes in cases of rotavirus in low-resource settings. It’s also important to provide closure to the case, including details of what the typical course would be prior to any final outcome. It is important to address that unfortunately, many children die from diarrheal disease around the world, but especially when resources are limited. Here is one way to approach discussing the remainder of the case:
       1. Normally rotavirus resolves within days with appropriate resuscitation.
       2. The virus lasted longer in this patient due to malnutrition → poor mucosal healing.
       3. The patient became severely dehydrated, but no clean IV equipment for fluids.
       4. They attempted to resuscitate with continued ORS.
       5. Unfortunately he passes away the following day.
15. **Slide 15**
    1. This slide is where you can begin discussing global health more broadly, with a focus on how diarrheal disease affects children worldwide and how that compares to other causes of pediatric mortality. After discussing the above statistics, you can then ask students the following, to help them begin thinking about why these statistics exist and what factors led to the outcome of this case:
       1. Why did this particular patient get rotavirus?
       2. What outcome did you expect?
       3. How does this play out in a high-income setting?
    2. “An estimated 215,000 children died of rotavirus infections in 2013, accounting for 37% of diarrhea-related deaths worldwide, 92% of which occurred in low and lower-middle income countries.” <https://www.ncbi.nlm.nih.gov/pmc/articles/PMC6183203/>
16. **Slide 16**
    1. This is an important slide discussing that pediatric mortality from diarrheal disease and malnutrition is **not** limited to low-income countries. This provides an opportunity to discuss challenges faced in the U.S. with food insecurity and how social determinants of health play a role in patient outcomes. You can also discuss ways in which food insecurity is being addressed in the U.S., if there is time. Here are a few talking points to consider:
       1. The U.S. is not exempt from pediatric mortality from diarrheal illness.
       2. Mortality from diarrheal illness is linked to malnutrition and food insecurity.
       3. This disproportionately affects underserved communities.
       4. This also exacerbates disparities in race, socioeconomic status, education, and more.
17. **Slide 17**
    1. This is an additional image that helps students understand levels of food insecurity and how food insecurity impacts individuals’ health. You can pause to discuss these images a bit further and ask students what additional ways they think food insecurity might impact their patients.
    2. Health impacts of food insecurity include:
       1. Inadequate intake of nutrients
       2. Risk for chronic disease
       3. Risk for negative pregnancy outcomes
       4. Long-term neurologic deficits
       5. Risk for negative mental health outcomes
18. **Slide 18**
    1. This is an important opportunity to discuss the Rotavirus vaccine. You can discuss practically how it is administered and who receives it. We have also included information on the slide regarding rates of vaccination and its effectiveness. If there is time, you can also discuss the policy issues behind implementing the Rotavirus vaccine, including changes when the side effect of intussusception was first discovered. We have included additional information below, should you want to expand on the rotavirus vaccine more.
       1. Fantastic brief article on Rotavirus in LMICs: <https://www.thelancet.com/journals/laninf/article/PIIS1473-3099(19)30263-4/fulltext>
       2. Another review of RCTs on Rotavirus: <https://www.thelancet.com/journals/laninf/article/PIIS1473-3099(19)30126-4/fulltext>
       3. Cost-effectiveness study: <https://www.ncbi.nlm.nih.gov/pubmed/17403839>
19. **Slide 19**
    1. This slide is an opportunity to discuss prevention strategies for malnutrition and diarrheal disease. You can take this opportunity to discuss why some of these strategies are difficult in low-resource settings (such as hand-washing when there isn’t access to running water).
20. **Slide 20**
    1. This is a great opportunity to ask students if they have additional questions and also to talk about your career. We recommend including picture(s) from your global health work or anything else from you career that you think would be helpful for the students.
    2. This is also a time to encourage students to fill out the post-didactic survey, which will help gather data on how successful the session was and will gather feedback on how to improve future sessions.

Surgery & Global Health (Global Surgery)

1. **Slide 1**
   1. None
2. **Slide 2**
   1. This slide includes a description of the setting (rural Rwanda), including living conditions and available healthcare resources. This helps frame the way students will approach the case throughout the session.
3. **Slide 3**
   1. This is a good point to pause and ask the students in the room about their prior experiences in global health and general clinical care:
      1. Has anyone done global health work before?
      2. Have you taken care of patients with burns? Either here or elsewhere?
      3. What happened in that case?
4. **Slide 4**
   1. This is an opportunity to help students build on their history skills. You can ask students the following:
      1. Does anything in particular from the history stand out?
5. **Slide 5**
   1. This slide includes photos of the girl mentioned in this case. It includes the initial stages of when the patient first arrived at the tertiary hospital in Rwanda. This is an opportunity to help students build on their observation skills. You can ask students the following:
      1. What do you notice in these pictures?
      2. What physical exam would you want to do based on the patient’s history and these pictures?
6. **Slide 6**
   1. This is an opportunity to help students build on their physical exam skills. You can ask students the following:
      1. What aspects of this physical exam are concerning?
      2. What do you think overall is going on?
   2. You can also discuss that patients with significant burns can have mixed hypovolemic / distributive shock picture. Thus, it is important to carefully monitor: heart rate, blood pressure, distal pulses, cap refill, and color/turgor of uninjured skin every hour for 24 hours.
7. **Slide 7**
   1. This slide is a great opportunity to work on students’ understanding clinical management of burn patients.
   2. This is also a great opportunity to discuss the ABCs of trauma care. Here are some guidelines for what you can talk students through:
      1. Airway - Do we need to intubate?
      2. Breathing - CO poisoning? Inhalation injury?
      3. Circulation - Severity of burns? Fluid requirement?
         1. Start fluids
         2. CVC + Arterial line
      4. Other
         1. Analgesia
         2. Make NPO & place NGT
         3. Foley catheter to measure UOP
         4. Nebulizers for smoke inhalation injury
         5. Tetanus & ulcer prophylaxis
8. **Slide 8**
   1. This slide is an opportunity to discuss what labs students would normally want to order in a high-resource setting for a patient with burns. Start off by posing the question above to the students and allowing them to think through this. If there is time, you can push students to explain why they would want certain labs and what they would expect as possible lab values (or ranges). *This is also an important place to discuss what labs would or wouldn’t be available in a low-resource setting, and how you would approach this case differently because of that.*
   2. **CBC, BMP, lactate, ABG, PT/INR, CK, UA**
9. **Slide 9**
   1. This slide is for discussing the Parkland formula for administering IV fluids to burn patients. You can start off by posing the the two questions on the slide and eliciting what students already may or may not know about the Parkland formula. You can then touch on the following points:
      1. Can discuss how the Parkland formula is used as a “starting point” for fluid management, but that a more sophisticated approach, including monitoring urine output, is important (see next slide)
      2. Maintenance fluids are typically administered after the first 24 hours, if the patient is appropriately responding to resuscitation
      3. Different types of IV fluids (normal saline, Lactated Ringers, D5W, 1/2NS, etc.) and what type is preferred.
10. **Slide 10**
    1. This slide is an opportunity to build on students clinical knowledge to discuss the other considerations when administering IV fluids to burn patients. The slide includes goals based on urine output, as well as other interventions and testing. Additional factors to think about when caring for these patients include: age, severity of burns, associated injuries (i.e. inhalational), and comorbidities.
11. **Slide 11**
    1. This slide also focuses on clinical knowledge to wrap up the discussion on fluid administration and why it is important to appropriately, and not excessively, give IV fluids to burn patients. You should start by posing the question above and eliciting answers from the students.
12. **Slide 12**
    1. This is an opportunity to pause and discuss how fluid resuscitation happens in low-resource settings, and how this is often limited due to lack of access to IV fluids, unlike many of the academic centers that students often work in.
13. **Slide 13**
    1. This slide is meant to show the impact of burns on children worldwide, as it represents the 5^th^ most common cause of non-fatal childhood injuries worldwide.
14. **Slide 14**
    1. This is an image representing the mortality rate from burns in young children. This image is meant to reflect the significant disparities between those in high-income vs. low-income countries. Additional facts on burn epidemiology include the following:
       1. 2.5 children per 100,000 die from burns each year
       2. The largest burden of burns and mortality from burns is in Sub-Saharan Africa
       3. There is a huge disparity in pediatric burns between high-income and low-income countries
15. **Slide 15**
    1. This slide is meant to focus on surgical approaches to burns and the importance of early excision & grafting in preventing future contractures. Depending on how much time you have in the session, you can go into a detailed discussion of debridement (and why it is done) as well as grafting approaches. It is important to discuss the limited availability of surgical care in low-resource settings and the lack of access to early excision & grafting for patients. This is in part due to lack of available equipment, operating rooms, and surgical providers. In addition, when grafting isn’t possible due to limited intact skin, low-resource settings do not often have alternative methods, such as cadaveric specimens or artificial grafts listed above.
16. **Slide 16**
    1. slide brings students back to the case, discussing what happened with initial management (debridement outside of the operating room setting) and what the patient wasn’t able to receive (skin grafts). It also touches on additional challenges the patient faced due to underlying conditions (malnutrition) from living in a low-income country. Because this patient was able to receive skin grafts in a timely manner, she was then put at risk for weight loss, poor immune response, and infections. You can touch on these additional points below:
       1. Important to speak to lack of resources in LMICs and lack of access to burn surgery, leading to extensive hospitalization prior to appropriate treatment.
       2. In patients like these, you worry about weight loss, electrolyte imbalance, chronic tachycardia, poor immune system, and edema while in hospital.
17. **Slide 17**
    1. This is an opportunity to discuss the role of innovation in low-resource settings to provide care for patients. The Meek Technique was an innovative approach used by the Rwandan surgeons to provide grafts for this patient, given that she had very little remaining healthy skin. Essentially, instead of mesh split-thickness skin graft, which is stretched over a large area, you do the following (as depicted in the images above):
       1. Take small pieces of skin (4x4cm), cut into 196 mini squares (3x3mm) using a hand-pressed machine w/ blades
       2. Spray glue on outer side, press into gauze, and then pull out to expand
       3. This increases the surface area of coverage by 1400% since the edges are where epithelialization occurs
18. **Slide 18**
    1. These are additional pictures of the team that cared for this patient. They started with an anterior approach to address half of her burns, which amounts to 20% TBSA.
19. **Slide 19**
    1. These are additional pictures showing the patient’s continuous improvement after surgery. This was an incredible outcome for this patient, but it is important to emphasize that many burn cases do not have positive results. (See next slide)
20. **Slide 20**
    1. Building off of the points in the previous slide, this slide provides an opportunity to discuss outcomes for patients who ultimately are not able to access care (such as burn contractures), including downstream effects (such as difficulty attending school). You can also discuss the “delays to care” model from the Lancet Commission on Global Surgery 2030 report: <https://www.thelancet.com/pdfs/journals/lancet/PIIS0140-6736(15)60160-X.pdf>
21. **Slide 21**
    1. This slide goes into broad aspects of global surgery as a field. In particular, this slide includes key facts from the Lancet Commission on Global Surgery 2030 report (<https://www.thelancet.com/pdfs/journals/lancet/PIIS0140-6736(15)60160-X.pdf>) that are important for students to learn about.
22. **Slide 22**
    1. This slide builds on the previous slides, discussing current interventions to help build surgical systems in low-resource settings. In particular, this slide focuses on National Surgical Obstetric and Anesthesia Plans (NSOAPs) and the value of capacity-building through partnerships with Ministries of Health (MoH). Additional information on NSOAPs can be found here: <https://www.thelancet.com/pdfs/journals/lancet/PIIS0140-6736(15)60160-X.pdf>
23. **Slide 23**
    1. This slide is an opportunity to discuss burn epidemiology. Pertinent points are listed in the slide above.
24. **Slide 24**
    1. This is an important opportunity to pause and ask students why they felt this patient was at risk for burns and what led to their prolonged hospitalization and outcome. You can pose the following questions:
       1. Why did this particular patient get burns? What put them at higher risk?
       2. What would have happened if she developed contractures?
       3. How do you think a case like this plays out in a high-income setting?
    2. Some potential answers that students may give include:
       1. Child living in rural area
       2. Parents working in the field (with her alone at home)
       3. Cooking over fire outdoors (not safe)
       4. Lack of access to surgical care
       5. Didn’t get treatment right away (in the hospital for 16 months until able to get the procedure)
25. **Slide 25**
    1. This is an important slide on the many risk factors for burns. This is an opportunity for students to think through how these risk factors affects those in low-income countries but also those in high-income countries like the United States. This will flow directly into the next slide on vulnerable populations in the U.S.
26. **Slide 26**
    1. This slide includes important facts about populations in the U.S. that are particularly vulnerable to burns. You may consider starting by sharing any examples of burn patients in your locality to help engage students who are less interested in global health. This slide really emphasizes that students will encounter social determinants of health regardless of the setting they work in.
    2. You can also emphasize the need for increased housing stability, mental health services, addiction services, and opportunities for homeless individuals to reintegrate into society – structural approaches to decrease risk of burns for these individuals.
27. **Slide 27**
    1. This slide is focused on “next steps” to help students think about awareness, prevention, and strengthening surgical systems. You can build on this based on any of your prior work, or if you are running out of time, you can move to the final slide to conclude the session.
28. **Slide 28**
    1. This is a great opportunity to ask students if they have additional questions and also to talk about your career. We recommend including picture(s) from your global health work or anything else from you career that you think would be helpful for the students.
    2. This is also a time to encourage students to fill out the post-didactic survey, which will help gather data on how successful the session was and will gather feedback on how to improve future sessions.

Medicine & Global Health

1. **Slide 1**
   1. None
2. **Slide 2**
   1. This slide and the notes below include a description of the setting (Soroka Medical Center, Negev Desert in Israel), including living conditions and available healthcare resources. This helps frame the way students will approach the case throughout the session.
   2. Here are additional points about the setting to help frame the case:
      1. Overall, Israel is a middle-income country with $36K GDP/capita; however, there are significant disparities in care across the country, including in rural areas.
      2. The Negev desert is the largest but least populated area of Israel. The patient in this case is from the Negev desert.
      3. Soroka Medical Center serves the entire region of the Negev desert.
3. **Slide 3**
   1. This is a good point to pause and ask the students in the room about their prior experiences in global health and general clinical care:
      1. Has anyone done global health work before?
      2. Have you taken care of patients with shortness of breath and a fever? Either here or elsewhere?
      3. What happened in those cases?
4. **Slide 4**
   1. This is an opportunity to help students build on their history skills. You can ask students the following:
      1. Does anything in particular from the history stand out?
5. **Slide 5**
   1. This is an opportunity to help students build on their physical exam skills. You can ask students the following:
      1. What aspects of this physical exam are concerning?
      2. What do you think overall is going on?
6. **Slide 6**
   1. This is an opportunity to focus in on the pulmonary exam to emphasize clinical skills for the students. You can emphasize the following points:
      1. The pulmonary exam is particularly important in low-resource settings and rural clinics when imaging isn’t available.
      2. Egophony is the main physical exam maneuver that can be particularly helpful in diagnosing pneumonia.
   2. *You may consider pausing here and having students practice egophony on each other!
7. **Slide 7**
   1. This slide is an opportunity to discuss what labs students would normally want to order for a patient with shortness of breath and fever. Start off by posing the question above to the students and allowing them to think through this. If there is time, you can push students to explain why they would want certain labs and what they would expect as possible lab values (or ranges). *This is also an important place to discuss what labs would or wouldn’t be available in a low-resource setting, and how you would approach this case (or other cases) differently because of that.*
   2. *The next slide shows real lab values from this case.
8. **Slide 8**
   1. Building on the previous slide, you can ask students the following:
      1. What do you notice in these labs?
      2. Is there anything concerning that stands out? Why?
9. **Slide 9**
   1. This slide is a great opportunity to go over how to read a chest Xray with the students. You can also discuss typical vs atypical pneumonia, what they might look like on a CXR, and what type of symptoms / disease course to expect. At Soroka Medical Center, CXRs are available, but you may want to also discuss that in rural clinics and other low-resource health centers, you may not have CXRs as an option for diagnosis.
10. **Slide 10**
    1. This slide is to encourage students to think about the differential for the patient’s shortness of breath and fever. After they form a comprehensive differential, it is helpful to follow-up and ask students what they think is most likely in this case.
11. **Slide 11**
    1. This slide is meant to show the severe impact of air pollution, which is the 5^th^ highest risk factor for mortality globally. This transitions well from the clinical discussion in the previous slides to social determinants of health (in this case, pollution) and their affects on health. This will set up later slides in the case that discuss why this particular patient became sick.
12. **Slide 12**
    1. This is an opportunity to further discuss mortality from air pollution and why. You can emphasize the increased risk for pneumonia, stroke, ischemic heart disease, COPD, cancer, and more. Indoor air pollution accounts for up to 5 million deaths per year worldwide. Low-income countries are associated with having increased pollution.
    2. This is also an opportunity to build further on this case with additional history on the patient. The patient in this case was later found to have COPD and some bronchiectasis thought to be due to cooking indoors with solid fuels. This may have predisposed her to pneumonia.
13. **Slide 13**
    1. This slide is an opportunity to tie in the “global is local” concept by discussing the effects of pollution here in the U.S. In the U.S., people with lower socioeconomic status are more likely to live in areas with worse air pollution (i.e. near power plants, refineries, diesel engines). Examples include increased rates of asthma in people of color and difficulty controlling chronic medical issues in those living in impoverished communities.
14. **Slide 14**
    1. If time allows, this is an opportunity to touch on how air pollution contributes to climate change, which then disproportionately affects underserved communities worldwide.
15. **Slide 15**
    1. Here you can transition back to the case. You can start by asking students the questions posed in the slide:
       1. What do you think is going on?
       2. What treatment do you start with?
       3. What additional tests do you want to run and why?
    2. In this case, the patient had access to and was started on ceftriaxone and azithromycin. If there is time, you can discuss antibiotic choice. The patient also required significant oxygen supplementation.
    3. Students may consider the following additional tests:
       1. Blood culture, sputum culture, urine culture
       2. Influenza PCR
       3. QuantiFERON TB gold
    4. It is important to comment, again, that while you may order these tests in the U.S. or at this tertiary care facility in Israel, you would not necessarily be able to do so at rural healthcare centers in rural or low-resource settings. It is also important to note that initial treatments of antibiotics and even oxygen are often not possible in low-resource settings.
16. **Slide 16**
    1. After 4 days of the treatment in the previous slide, the patient is not improving. This slide is an opportunity to encourage students to broaden their differential. You may start off by asking what other pathogens may not have been accounted for in the initial differential. It is important to comment on how knowledge of local epidemiology can help inform you when culture data isn’t available, and can help guide additional treatment modalities.
17. **Slide 17**
    1. This patient improved on doxycycline. You can use this slide as an opportunity to discuss the feasibility for patients to travel to a referral hospital such as this patient who traveled from within the Negev desert to get to a tertiary care center. You can also discuss the challenges that many patients face in paying for their healthcare in scenarios such as this.
18. **Slide 18**
    1. This slide discusses Q fever in more detail. This is not an infectious pathogen that medical students commonly come across, so you can briefly talk about the pathogen itself and its epidemiology.
19. **Slide 19**
    1. This builds on the previous slide by discussing why certain patients may be at higher risk of C. burnetii (Q fever) infection than others and why rates of Q fever are higher in rural areas of Israel, such as where this patient came from.
    2. The above picture is a typical scene in the Negev desert area. It has been shown that 25% of livestock in Israel have evidence of C. burnetii infection.
20. **Slide 20**
    1. This slide focuses on clinical knowledge, emphasizing that doxycycline is the appropriate treatment for Q fever infection. It includes information on dosing, length of treatment, and other necessary tests for these patients. This is a great opportunity to point out that while in many high-resource settings, you would not usually treat CAP with doxycycline, empiric coverage can be different in different settings. In southern Israel, you may want to start CAP patients with doxycycline, knowing the prevalence of infections such as Q fever that would be treated with that regimen.
21. **Slide 21**
    1. This slide continues the discussion on clinical manifestations of Q Fever, now with a focus on long term health effects. As mentioned in Slide 13, the patient had COPD and bronchiectasis from solid fuel inhalation from indoor cooking. This is especially common in women who live in low-resource settings, which already put this patient at risk. Now with the potential for additional long-term health effects from Q Fever, her quality of life may be significantly decreased in the future. It is important to discuss that while not all outcomes are immediately negative in these patients, there may be long-term effects that impact their ability to work or contribute to their family’s needs, leading to future negative outcomes overall.
22. **Slide 22**
    1. This is a good opportunity to take a deep dive into Social Determinants of Health. You may consider posing the following question to students:
       1. Why did this patient get sick rather than someone else? What puts someone at risk?
23. **Slide 23**
    1. *****Note:** We encourage those teaching this case to take an apolitical approach to this topic and to focus on the challenges faced by populations displaced in any capacity. We recommend less emphasis on why this population was displaced, but instead to focus on the difficulties encountered both during and after such a process. If you have questions about how to approach this discussion, please reach out to one of the co-authors: **Dr. Bram Wispelwey** at **bwispelw@gmail.com**.
    2. Forced Displacement is defined as coerced movement from one’s home or homeland; when one ethnic group is displaced by a more powerful ethnic group. This slide is an opportunity to discuss how loss of land, migration, and displacement can affect people’s health. In particular, for this patient, who represents the 1/3^rd^ of the Negev desert population that was displaced in the 1950s and 1960s. (See next slide for additional image.)
    3. Initially, much of Southern Israel was inhabited by a Bedouin population. That land later was reclaimed by the state of Israel, leaving many of those who stayed to be considered “unrecognized groups.” This population has had a rapidly increasing burden of diabetes mellitus, hypertension, and other chronic conditions, and have also been exposed to high levels of poverty, unemployment and crime. Ambulances often will not come into unrecognized villages, posing significant challenges for patients, such as the one in this case, to access care.
24. **Slide 24**
    1. This is an opportunity to connect the case to an important issue here in the U.S.: Native American healthcare. In particular, the above image focuses on loss of Native American land throughout the 1800s and 1900s. You may consider asking students the following questions to help them further reflect on the implications of this:
       1. What is the legacy of medicine in the community in which you practice?
       2. What about the legacy of medicine for the black community in the U.S.? For indigenous people?
25. **Slide 25**
    1. This slide is an opportunity to talk about challenges faced by displaced and impoverished communities in your own locality. We recommend adapting this slide based on your local context to allow students to connect the concepts from this session with issues they are more familiar with and that are directly relevant to their patients.
26. **Slide 26**
    1. This slide is an opportunity for a “final wrap-up” to encourage students to reflect on their learning throughout the case. You can start by broadly posing the question of “Why did this patient get sick?” While some students may say “exposure to livestock” or “air pollution,” it is important to probe further and encourage students to consider the social factors that led to this patient’s illness. Ideally, students will make the connection between forced displacement & historical factors, forced changes in lifestyle, poverty, lack of self-determination, and resulting exposures / risk factors.
27. **Slide 27**
    1. This is a great opportunity to ask students if they have additional questions and also to talk about your career. We recommend including picture(s) from your global health work or anything else from you career that you think would be helpful for the students.
    2. This is also a time to encourage students to fill out the post-didactic survey, which will help gather data on how successful the session was and will gather feedback on how to improve future sessions.
