# Supplementary material for: A Longitudinal Case-Based Global Health Curriculum for the Medical Student Clerkship Year
Source: MedEdPORTAL. 2020 Dec 8;16:11038. doi: 10.15766/mep_2374-8265.11038 (PMC7732136; doi:10.15766/mep_2374-8265.11038)
Supplement: Supplementary file 1 — Clerkship Director Proposal.pptxProject Description.docxPediatrics GH Didactic.pptxSurgery GH Didactic.pptxMedicine GH Didactic.pptxFacilitator Notes.docxPredidactic Survey.docxPostdidactic Survey.docxFollow-up Survey.docx [file mep_2374-8265.11038-s001.zip › G. Predidactic Survey.docx]

**[Pre-Survey] Clerkship Global Health Curriculum**

1. What is your gender?
   1. Female
   2. Male
   3. Non-binary
   4. Other: _________
   5. Prefer not to say
2. Program
   1. [customize to institution]
3. Year
   1. MS1
   2. MS2
   3. MS3
   4. MS4
4. What field(s) are you broadly interested in?
   1. Internal Medicine
   2. Surgery
   3. Ob/Gyn
   4. Pediatrics
   5. Neurology
   6. Psychiatry
   7. Radiology
   8. Other: _________
5. Have you done global health work or research in the past?
   1. Yes
   2. No
6. If so, please briefly describe your prior global health experience.
   1. ______________________________________________
7. How interested are you in global health?
   1. Extremely interested
   2. Very interested
   3. Moderately interested
   4. Slightly interested
   5. Not at all interested
8. How likely are you to seek out a global health experience during **medical school?**
   1. Extremely likely
   2. Somewhat likely
   3. Neither likely nor unlikely
   4. Somewhat unlikely
   5. Extremely unlikely
9. How likely are you to seek out a global health experience during **residency?**
   1. Extremely likely
   2. Somewhat likely
   3. Neither likely nor unlikely
   4. Somewhat unlikely
   5. Extremely unlikely
10. How likely are you to seek out a global health experience during your **future medical career?**
    1. Extremely likely
    2. Somewhat likely
    3. Neither likely nor unlikely
    4. Somewhat unlikely
    5. Extremely unlikely
