# Supplementary material for: A Longitudinal Case-Based Global Health Curriculum for the Medical Student Clerkship Year
Source: MedEdPORTAL. 2020 Dec 8;16:11038. doi: 10.15766/mep_2374-8265.11038 (PMC7732136; doi:10.15766/mep_2374-8265.11038)
Supplement: Supplementary file 1 — Clerkship Director Proposal.pptxProject Description.docxPediatrics GH Didactic.pptxSurgery GH Didactic.pptxMedicine GH Didactic.pptxFacilitator Notes.docxPredidactic Survey.docxPostdidactic Survey.docxFollow-up Survey.docx [file mep_2374-8265.11038-s001.zip › H. Postdidactic Survey.docx]

**[Post-Survey] Clerkship Global Health Curriculum**

1. How interested are you in global health?
   1. Extremely interested
   2. Very interested
   3. Moderately interested
   4. Slightly interested
   5. Not at all interested
2. How likely are you to seek out a global health experience during **medical school?**
   1. Extremely likely
   2. Somewhat likely
   3. Neither likely nor unlikely
   4. Somewhat unlikely
   5. Extremely unlikely
3. How likely are you to seek out a global health experience during **residency?**
   1. Extremely likely
   2. Somewhat likely
   3. Neither likely nor unlikely
   4. Somewhat unlikely
   5. Extremely unlikely
4. How likely are you to seek out a global health experience during your **future medical career?**
   1. Extremely likely
   2. Somewhat likely
   3. Neither likely nor unlikely
   4. Somewhat unlikely
   5. Extremely unlikely
5. How interested are you in having more global health didactics in the **clerkships?**
   1. Extremely interested
   2. Very interested
   3. Moderately interested
   4. Slightly interested
   5. Not at all interested
6. How interested are you in having more global health didactics in the **overall medical school curriculum?**
   1. Extremely interested
   2. Very interested
   3. Moderately interested
   4. Slightly interested
   5. Not at all interested
7. Please name at least one aspect of this global health didactic session that you found helpful.
   1. ________________________________________________
8. Please name at least one aspect of this global health didactic session that you would improve.
   1. ________________________________________________
9. If you have additional feedback, please feel free to include below:
   1. ________________________________________________
