# Supplementary material for: A Longitudinal Case-Based Global Health Curriculum for the Medical Student Clerkship Year
Source: MedEdPORTAL. 2020 Dec 8;16:11038. doi: 10.15766/mep_2374-8265.11038 (PMC7732136; doi:10.15766/mep_2374-8265.11038)
Supplement: Supplementary file 1 — Clerkship Director Proposal.pptxProject Description.docxPediatrics GH Didactic.pptxSurgery GH Didactic.pptxMedicine GH Didactic.pptxFacilitator Notes.docxPredidactic Survey.docxPostdidactic Survey.docxFollow-up Survey.docx [file mep_2374-8265.11038-s001.zip › I. Followup Survey.docx]

**[Follow-Up] Clerkship Global Health Curriculum**

1. How did the global health didactic session influence your view of taking care of patients here?
   1. __________________________________________________________________
2. What similarities did you notice between issues faced by the patient in the global health didactic session and challenges your patients here have faced?
   1. __________________________________________________________________
3. Do you have any other reflections on this clerkship global health curriculum initiative or the global health curriculum offerings at [your institution]? If so, please include them below:
   1. __________________________________________________________________
